# A survey of transformer-based architectures in medical image analysis: models, applications, and challenges

**DOI:** 10.3389/frai.2026.1873938

**Published:** 2026-08-06

**Authors:** Sam Ansari, Nastaran Faraji, Luke Topham, Wasiq Khan, Dina Shehada, Soliman Mahmoud, Hissam Tawfik, Abir J. Hussain

**Affiliations:** 1 Research Institute of Sciences and Engineering, University of Sharjah, Sharjah, United Arab Emirates; 2 Department of Electrical Engineering, College of Engineering, University of Sharjah, Sharjah, United Arab Emirates; 3 School of Computer Science and Mathematics, Faculty of Engineering, Liverpool John Moores University, Liverpool, United Kingdom; 4 College of Engineering & IT, University of Dubai, Dubai, United Arab Emirates

**Keywords:** deep learning in medical imaging, image segmentation and classification, medical image analysis, transformer architectures in medical imaging, Vision Transformers

## Abstract

Transformer-based architectures have become central to medical image analysis, yet their practical value remains difficult to assess because studies vary widely in tasks, datasets, validation protocols, baselines, and reporting quality. This survey critically reviews recent transformer-based, hybrid, foundation, and transformer-alternative models across segmentation, classification, reconstruction, and image registration. A total of 128 studies published between 2021 and 2026 are organized using a task-, modality-, and architecture-aware taxonomy, with reported performance synthesized alongside baseline comparisons, reproducibility, computational cost, and clinical-readiness evidence. The findings indicate that the most convincing gains arise from task-adapted hybrid designs that combine local feature extraction with global context modeling, rather than from an unconditional superiority of transformers over convolutional networks. Persistent gaps include non-standardized benchmarks, limited external validation, incomplete code and weight availability, inconsistent efficiency reporting, weak uncertainty analysis, and insufficient clinical evaluation. Progress will require transparent reporting, multicenter validation, and clinically grounded assessment.

## 1 Introduction

Medical image analysis plays a central role in modern healthcare (Wang J. et al., 2025), enabling the detection, diagnosis, and monitoring of a wide range of diseases through imaging modalities such as magnetic resonance imaging (MRI), computed tomography (CT), positron emission tomography (PET), and ultrasound (Wang et al., 2021). With the growing volume and complexity of medical imaging data, automated interpretation methods have become essential for improving diagnostic accuracy and reducing clinical workload. Traditional machine learning and early deep learning approaches, particularly convolutional neural networks (CNNs), have significantly advanced image-based medical tasks (Takahashi et al., 2024). In medical image analysis, the transition from conventional CNNs to transformer-based architectures has opened new directions for research. CNNs are inherently limited in modeling long-range dependencies due to their local receptive fields, which can be a constraint when analyzing complex medical images that often require global context understanding (Azad et al., 2024; Li et al., 2023). Transformer-based models, with their global receptive fields and scalable architectures, offer improved performance in various medical imaging tasks, such as segmentation, classification, detection, and registration (Khan et al., 2025).

Transformers have emerged as a powerful class of deep learning models, initially designed for natural language processing (NLP) tasks due to their ability to model sequential data through attention mechanisms (Wolf et al., 2020). Unlike recurrent neural networks (RNNs), long short-term memory (LSTM), and CNNs, transformers process input in parallel and emphasize relevant information using self-attention, allowing them to capture global contextual relationships more effectively. This capability has led to their rapid adoption beyond NLP, including significant progress in computer vision and, more recently, in medical image analysis (Thirunavukarasu and Kotei, 2024).

The core mechanism enabling the performance of transformer models is the attention mechanism, which assigns weights to input elements based on their relevance to each other. In the original Transformer architecture, input sequences are processed through encoder and decoder modules, each composed of multiple layers of multi-head attention and feed-forward networks. This structure eliminates the need for recurrence by introducing positional encodings to preserve the order of input data. While initially tailored for text, this architecture has since been adapted to handle spatial data in images by treating image patches as tokens and applying similar attention-based operations. Vision Transformers (ViTs), Swin Transformers (ST), and related variants have demonstrated competitive or superior performance compared to CNNs on medical datasets including chest X-rays, retinal fundus images, brain MRIs, and histopathological slides (Aburass et al., 2025; Li et al., 2023). Moreover, hybrid models combining CNNs and transformers are being explored to leverage the strengths of both local feature extraction and global attention. These developments suggest that transformers hold significant potential in advancing the state of the art in medical image analysis (Wu et al., 2023; Kim et al., 2025; Gao et al., 2025). Despite these advances, several challenges remain. Medical imaging data is often high-dimensional and lacks sufficient annotated samples for training large transformer models, which are typically data-intensive. Furthermore, the clinical deployment of these models demands interpretability, robustness, and computational efficiency, factors that must be carefully addressed to ensure real-world applicability.

Several review articles have examined Transformer-based medical image analysis from different perspectives, including comprehensive surveys, disease-specific applications, segmentation, and hybrid architectures (Li et al., 2023; Kim et al., 2025; Azad et al., 2024; Takahashi et al., 2024; Aburass et al., 2025; Khan et al., 2025). While these reviews provide valuable insights, they differ in their scope, literature coverage, and research focus. To better position the contribution of the present survey, Table 1 compares representative review articles and highlights how this survey extends prior work through broader task coverage, updated literature coverage, and a descriptive quantitative synthesis.

**Table 1:** Comparison of representative review articles highlighting the scope and contributions of the present survey.

Survey	Year	Coverage	Papers	Scope	Focus	Key contribution
(Li et al. 2023)	2023	Up to 2022	114	General	Transformer architectures	Comprehensive review of Transformer architectures and applications in medical image analysis.
(Azad et al. 2024)	2024	Up to 2023	NR	General	Vision Transformers	Comprehensive review of Vision Transformer methods for medical image analysis.
(Takahashi et al. 2024)	2024	2021–2023	36	General	CNN vs. Vision Transformers	Systematic comparison of Vision Transformers and CNNs in medical image analysis.
(Aburass et al. 2025)	2025	Up to 2024	NR	Disease-specific	Vision Transformers	Review of Vision Transformer applications across multiple diseases.
(Khan et al. 2025)	2025	Up to 2024	NR	Segmentation	Vision Transformers	Focused review of Vision Transformer-based medical image segmentation.
(Kim et al. 2025)	2025	2020–September 2024	34	Radiology	Hybrid CNN–Vision Transformers	Systematic review of hybrid CNN–Vision Transformer architectures for radiological image analysis.
This survey	**2026**	**2021–June 2026**	**128**	**Segmentation, classification, reconstruction, registration**	**Transformer-based medical image analysis**	**Comprehensive review of 128 studies (2021–2026) covering segmentation, classification, reconstruction, and registration, with a descriptive quantitative synthesis and discussion of current challenges and future research directions**.

NR, Not explicitly reported by the authors.

This survey aims to provide a structured and comprehensive review of transformer-based architectures in medical image analysis, offering critical insights into their design, performance, and limitations. The discussion includes an overview of foundational principles, architectural adaptations for vision tasks, and applications across different imaging modalities and clinical tasks. A taxonomy is presented to categorize the surveyed methods, followed by an analysis of key challenges such as data scarcity, model complexity, and deployment constraints. By synthesizing existing work and identifying current trends, this survey contributes to a clearer understanding of how transformers are shaping the future of medical image analysis and highlights potential directions for further investigation.

Accordingly, this review focuses on models in which self-attention mechanisms, Transformer architectures, or Transformer-centered hybrid designs play a central role in feature extraction, contextual modeling, or performance improvement in medical image analysis. The review examines four major application areas: segmentation, classification, reconstruction, and image registration. This order is adopted to better reflect clinically grounded medical imaging workflows, in which lesion, organ, or anatomical delineation often provides the spatial foundation for downstream diagnostic classification, quantitative assessment, treatment planning, or predictive modeling. Segmentation is therefore discussed before classification, followed by reconstruction and registration, which address image recovery and spatial alignment tasks with distinct methodological and clinical requirements.

### 1.1 Scope of review and study selection criteria

The aim of this review is to provide a structured summary of the role of transformer-based architectures in medical image analysis, rather than to provide a completely independent and comprehensive review of each of the sub-domains of segmentation, classification, reconstruction, or image registration. Therefore, the scope of the article is limited to studies in which transformers, vision transformers, hierarchical transformers, self-attention-based models, or hybrid frameworks with a transformer component played a major role in the design of the method. The four application domains of segmentation, classification, reconstruction, and image registration are chosen because they cover the most reported applications of transformers in medical image analysis and allow for the examination of the functional, architectural, and clinical differences of this family of models across different tasks.

The studies included in this review are selected based on several main criteria: first, the study must be directly related to medical image analysis and must have examined clinical or biomedical imaging data, including CT, MRI, ultrasound, X-ray images, retinal images, dermoscopic images, or histological images. Second, the proposed or evaluated method must include a transformer architecture, a transformer-based hybrid model, or an explicit attentional component with a central role in feature extraction, spatial dependency modeling, or final prediction. Third, the study must have a reportable empirical evaluation, including metrics such as accuracy, sensitivity, specificity, Dice score, Jaccard index, mean error, execution time, or other indicators relevant to the task under study. Fourth, preference is given to more recent studies, studies with well-known datasets, and articles that have made a significant contribution to understanding current trends in the field in terms of architectural design, task type, imaging modality, or clinical outcome.

In contrast, studies that used only convolutional models or classical machine learning methods, unless reported as a baseline for comparison with transformers, are excluded from the main scope of the review. Articles lacking sufficient information about the dataset, evaluation criteria, or method design, studies unrelated to medical imaging, and works that only superficially or marginally mentioned transformers are also excluded from the main analysis. This delimitation is done to avoid excessive fragmentation, increase paper coherence, and provide a clear framework for comparing studies. Given the interdisciplinary and rapidly evolving nature of this field, rather than focusing on a single modality or task, this article attempts to present a coherent picture of the common patterns, architectural advantages, methodological limitations, and clinical translation challenges of transformer models in medical image analysis. Therefore, the comparisons made in the following sections should be interpreted as a descriptive and structured synthesis of the evidence reported in different studies, rather than as a direct and homogeneous comparison between all models, datasets, and assessment protocols. To clarify the boundaries of the review and to ensure methodological transparency, Table 2 summarizes the inclusion and exclusion criteria used to delimit the scope of the surveyed studies. This delimitation indicates that the aim of the paper is not to provide an unlimited coverage of all medical image analysis methods, but rather to focus on a structured review of studies in which transformer architectures or transformer-based hybrid models play a central role in method design and performance analysis.

**Table 2:** Scope delimitation and study selection criteria.

Delimitation axis	Included in the review	Excluded from the review
Model category	Transformer-based architectures, Vision Transformers, Swin Transformers, DETR-based models, transformer-centered hybrid models, and approaches in which attention mechanisms constitute a core methodological component.	Purely convolutional models, classical machine learning methods, and approaches in which attention or transformer modules are only marginally mentioned or not central to the proposed method.
Data type	Medical and biomedical imaging data, including CT, MRI, ultrasound, X-ray, retinal imaging, dermoscopy, histopathology, and related clinical imaging modalities.	Non-imaging data, purely textual data, biomedical signals without an imaging component, and general-purpose natural images outside the medical or biomedical domain.
Application task	Segmentation, classification, detection/localization, reconstruction, and registration tasks within medical image analysis.	Tasks outside medical image analysis or studies in which medical imaging is not the primary analytical target.
Performance reporting	Studies reporting empirical evaluation using task-relevant metrics such as accuracy, sensitivity, specificity, precision, F1-score, AUC, Dice score, Jaccard index, registration error, reconstruction quality measures, runtime, or computational cost.	Studies without sufficient quantitative evaluation, studies lacking clear dataset information, and papers with incomplete or non-interpretable experimental reporting.
Role in synthesis	Studies that contribute to understanding architectural trends, modality-specific applications, task-dependent performance, clinical relevance, methodological limitations, or emerging challenges of transformer-based models in medical imaging.	Duplicated studies, purely descriptive papers without methodological or empirical contribution, and studies lacking sufficient information for structured comparison or critical synthesis.
Purpose of comparison	Descriptive and structured synthesis of reported evidence across heterogeneous medical imaging tasks, datasets, and evaluation protocols.	Direct statistical ranking of heterogeneous studies as if they shared identical datasets, validation strategies, task definitions, and evaluation conditions.

The remainder of this paper is organized as follows. Section 2 presents the literature search strategy and study selection methodology, including the search sources, inclusion and exclusion criteria, screening process, data extraction strategy, and methodological considerations used to structure the review. Section 3 introduces the foundational principles of Transformer architectures, including self-attention, encoder–decoder structures, positional encoding, and their relevance to medical imaging. Section 4 reviews major vision-oriented Transformer architectures, including DETR, ViT, DeiT, and Swin Transformer, and discusses their architectural characteristics and suitability for medical image analysis. Section 5 presents the application-level synthesis of Transformer-based and related models across medical image segmentation, classification, reconstruction, and registration. This order is adopted to provide a clinically grounded progression from dense anatomical or lesion delineation to image-level predictive modeling, followed by image reconstruction and spatial alignment. This organization reflects the fact that segmentation frequently serves as an upstream step in clinical imaging workflows while preserving the methodological distinction among dense prediction, classification, reconstruction, and registration tasks. Section 6 provides a descriptive quantitative synthesis and critical discussion of the reviewed literature, including task-specific performance patterns, same-study comparisons with non-transformer baselines, reproducibility and open-source availability, computational-cost reporting, clinical-readiness considerations, limitations, and future research directions. Finally, Section 7 concludes the paper by summarizing the main findings and outlining the broader implications of Transformer-based architectures for reliable and clinically relevant medical image analysis.

## 2 Literature search strategy and study selection methodology

This article is designed as a structured, descriptive review of transformer-based architectures in medical image analysis. The aim of this review is to provide an analytical map of architectural trends, application areas, reported performance, methodological limitations, and challenges to clinical translation of transformer models. Therefore, this study is not a formal statistical meta-analysis, and the quantitative results reported in different studies are interpreted as descriptive syntheses of published evidence, rather than as pooled estimates of effect or direct ranking of heterogeneous studies.

To identify relevant studies, a literature search is conducted in PubMed/MEDLINE, IEEE Xplore, ScienceDirect, SpringerLink, Web of Science, Scopus, arXiv, and Google Scholar. The main search period is considered from January 2021 to June 2026, because the widespread use of vision transformers in medical image analysis increased mainly after the introduction and development of ViT-based models, Swin Transformer, and transformer-based hybrid models. However, some basic sources from before this period, including the main Transformer architecture paper, DETR, ViT, and Swin Transformer, are retained to explain the theoretical foundations and architecture.

The search strategy is based on a combination of three groups of keywords: first, keywords related to architecture, such as “Transformer,” “Vision Transformer,” “ViT,” “Swin Transformer,” “DETR,” “self-attention,” “cross-attention,” “hybrid transformer,” and “medical transformer”; second, keywords related to the field of medical imaging, such as “medical imaging,” “medical image analysis,” “radiology,” “MRI,” “CT,” “ultrasound,” “X-ray,” “histopathology,” “retinal imaging,” “dermoscopy,” and “PET”; and third, keywords related to analytical tasks, such as “segmentation,” “classification,” “detection,” “localization,” “registration,” “reconstruction,” and “super-resolution”. Various combinations of these keywords with logical operators are used to cover the main areas of the article. An example of a general search phrase is: combining terms related to transformers with terms related to medical imaging and then restricting the results to tasks such as segmentation, classification, detection, reconstruction, or image registration.

The Scopus search query is formulated as follows:


TITLE-ABS-KEY (
   (
   transformer* OR “vision transformer*” OR ViT OR “Swin
Transformer*” OR DETR
   OR “self-attention” OR “self attention” OR
“cross-attention”
   OR “cross attention” OR “hybrid transformer*” OR “medical transformer*” OR “Diffusion
Transformer*”
   OR “Transformer-GAN” OR “Perceiver IO” OR “foundation model*”
   OR “Segment Anything” OR SAM OR MedSAM OR “state space model*”
   OR SSM OR Mamba OR “Vision Mamba” OR “U-Mamba”
   OR KAN OR “Kolmogorov-Arnold Network*”
   )
   AND
   (
   “medical imaging” OR “medical image analysis” OR radiology OR MRI
   OR “magnetic resonance imaging” OR CT OR “computed tomography” OR
CBCT
   OR “cone-beam CT” OR ultrasound OR “X-ray” OR histopathology
   OR pathology OR “whole-slide image*” OR WSI OR microscopy
   OR “retinal imaging” OR fundus OR OCT OR dermoscopy
   OR PET OR “positron emission tomography”
   )
   AND
   (
   segmentation OR classification OR detection OR localization
   OR registration OR reconstruction OR “super-resolution”
   OR “super resolution” OR restoration OR denoising
   OR “artifact removal” OR synthesis OR generation
   OR “image translation” OR “motion compensation”
   )
)
AND PUBYEAR > 2020 AND PUBYEAR < 2027


Identified studies are first screened based on title and abstract. Then, the full text of potentially relevant studies is reviewed to determine whether they met the review scope and inclusion and exclusion criteria. The inclusion criteria are direct relevance to medical or biomedical image analysis; primary use of Transformer architectures, Vision Transformers, attention-based hierarchical models, or Transformer-based hybrid frameworks; empirical evaluation on a medical dataset; and reporting of extractable quantitative metrics such as accuracy, sensitivity, specificity, F1-score, AUC, Dice score, Jaccard index, registration error, PSNR, SSIM, runtime, number of parameters, or other task-relevant metrics.

In contrast, studies outside the field of medical imaging, articles that used only convolutional or classical machine learning methods, studies in which transformers did not play a central role, articles lacking adequate reporting on the dataset or evaluation protocol, duplicate studies, articles without full text available, and works that only superficially mentioned transformers are excluded from the main analysis. Non-transformer models are only included when they are used as a baseline, comparator, or contextual framework for interpreting transformer performance.

For each included study, key information is extracted, including architecture type, Transformer or attentional component, imaging modality, task, dataset, validation strategy, performance measures, comparative baselines, availability of code or trained weights, reported computational cost, and evidence relevant to clinical application.

**Figure 1:**
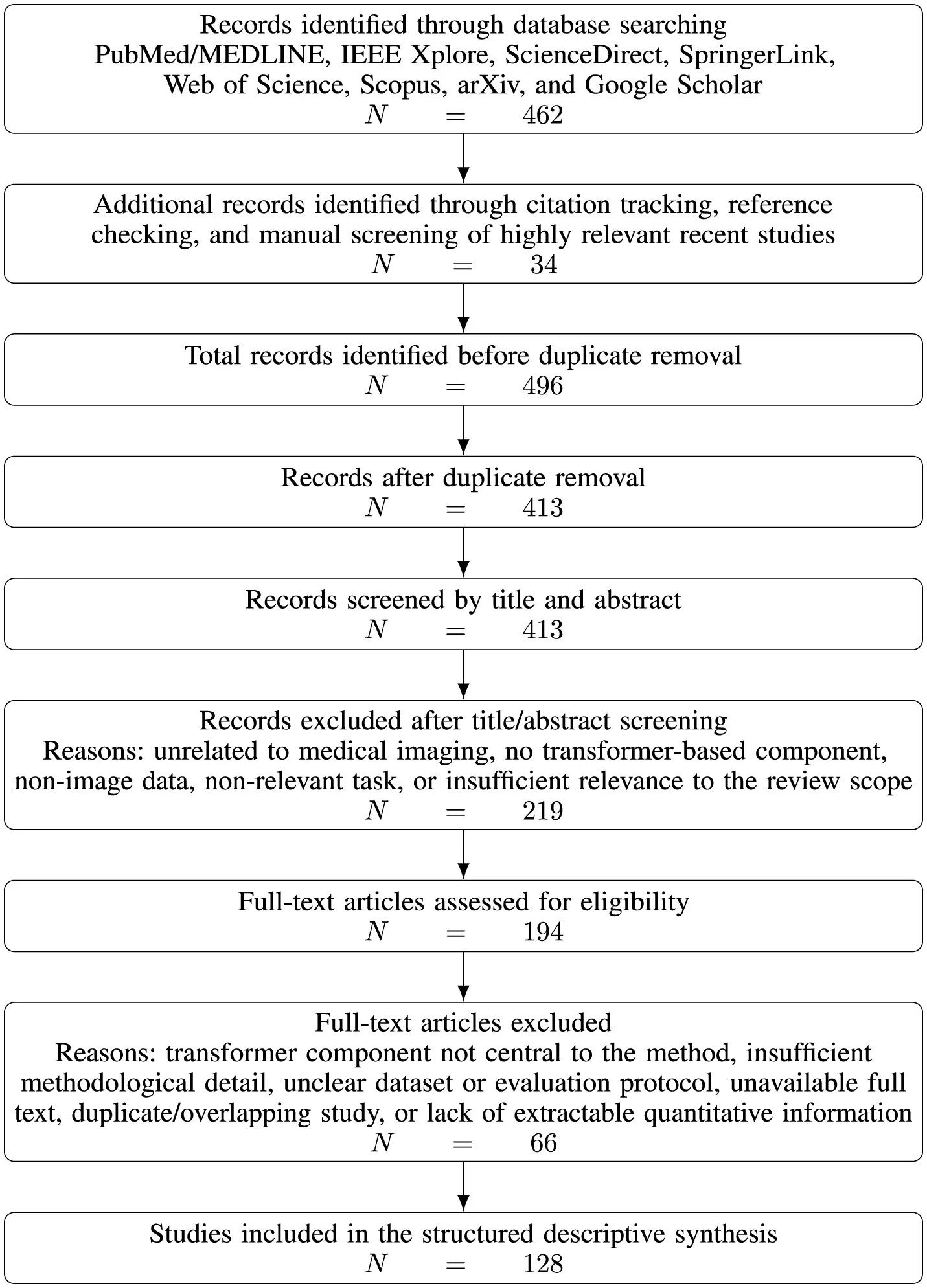
Flowchart of the literature identification, screening, eligibility assessment, and inclusion process.

The quality of studies is assessed based on a methodological checklist that included several main axes: transparency in the description of the dataset and data source, appropriate separation of training and test data, control of the possibility of data leakage, adequate reporting of evaluation criteria, existence of appropriate baselines, transparency in the validation protocol, reporting of computational cost or implementation limitations, availability of code or model weights, and relevance of results to clinical application. This assessment is not used to mechanically exclude studies, but rather to interpret results more cautiously, identify limitations of the available evidence, and avoid over-concluding about the overall superiority of transformers. The literature search strategy is summarized in Table 3, and the overall process of study identification, screening, eligibility assessment, and inclusion is illustrated in Figure 1.

**Table 3:** Summary of the literature search strategy.

Search component	Description
Review type	Structured descriptive review of transformer-based and transformer-centered hybrid architectures in medical image analysis.
Databases searched	PubMed/MEDLINE, IEEE Xplore, ScienceDirect, SpringerLink, Web of Science, Scopus, arXiv, and Google Scholar.
Search period	January 2021 to June 2026. Foundational works published before 2021 are retained when necessary to explain core architectural concepts, including the original Transformer, DETR, Vision Transformer, and Swin Transformer.
Architecture-related terms	*Transformer, Vision Transformer, ViT, Swin Transformer, DETR, self-attention, cross-attention, hybrid transformer, medical transformer, Diffusion Transformer, Transformer-GAN, Perceiver IO, foundation model, Segment Anything, MedSAM, state space model, Mamba, Vision Mamba, U-Mamba*, and *KAN*.
Medical-imaging terms	*medical imaging, medical image analysis, radiology, MRI, magnetic resonance imaging, CT, computed tomography, CBCT, cone-beam CT, ultrasound, X-ray, histopathology, pathology, whole-slide image, WSI, microscopy, retinal imaging, fundus, OCT, dermoscopy, PET*, and *positron emission tomography*.
Task-related terms	*classification, segmentation, detection, localization, registration, reconstruction, super-resolution, restoration, denoising, artifact removal, synthesis, generation, image translation*, and *motion compensation*.
Example Scopus query	TITLE-ABS-KEY((transformer* OR ~vision transformer*~ OR ViT OR ~Swin Transformer*~ OR DETR OR ~self-attention~ OR ~cross-attention~ OR ~hybrid transformer*~ OR ~medical transformer*~ OR ~Diffusion Transformer*~ OR ~Transformer-GAN~ OR ~Perceiver IO~ OR ~foundation model*~ OR ~Segment Anything~ OR SAM OR MedSAM OR ~state space model*~ OR SSM OR Mamba OR ~Vision Mamba~ OR ~U-Mamba~ OR KAN OR ~Kolmogorov-Arnold Network*~) AND (~medical imaging~ OR ~medical image analysis~ OR radiology OR MRI OR ~magnetic resonance imaging~ OR CT OR ~computed tomography~ OR CBCT OR ~cone-beam CT~ OR ultrasound OR ~X-ray~ OR histopathology OR pathology OR ~whole-slide image*~ OR WSI OR microscopy OR ~retinal imaging~ OR fundus OR OCT OR dermoscopy OR PET OR ~positron emission tomography~) AND (classification OR segmentation OR detection OR localization OR registration OR reconstruction OR ~super-resolution~ OR ~super resolution~ OR restoration OR denoising OR ~artifact removal~ OR synthesis OR generation OR ~image translation~ OR ~motion compensation~)) AND PUBYEAR > 2020 AND PUBYEAR < 2027
Screening process	Records are first screened by title and abstract. Potentially relevant studies are then assessed through full-text review according to the predefined inclusion and exclusion criteria.
Data extracted	Architecture type, transformer component, imaging modality, task, dataset, validation strategy, performance metrics, baseline comparisons, computational cost, code availability, and clinical-readiness indicators.
Quality assessment criteria	Dataset transparency, clarity of train/validation/test separation, risk of data leakage, appropriateness of evaluation metrics, availability of baselines, validation rigor, reproducibility, computational reporting, and clinical relevance.

## 3 Foundations of transformer architectures in vision and medical imaging

The Transformer model, introduced by Vaswani et al. (2017), marked a significant advancement in sequence transduction by departing from the reliance on recurrent and convolutional architectures. The primary innovation of the framework is the exclusive employment of attention mechanisms to model dependencies between sequence elements, eliminating the need for recurrence or convolutions. The design enables full parallelization during training and inference, significantly improving computational efficiency, particularly for long sequences. By attending to all positions within the input simultaneously, the Transformer effectively captures long-range dependencies, which are critical in tasks such as machine translation.

The Transformer architecture has emerged as a foundational model in deep learning, originally introduced for sequence transduction tasks in NLP. The inherent ability to model long-range dependencies through self-attention has enabled significant advancements in learning contextual relationships. These architectural features have proven valuable beyond the linguistic domain, particularly in computer vision and medical imaging (He et al., 2023), where the need for global context modeling and flexible feature extraction is critical. Section 3 provides an overview of the fundamental components of Transformer models, including attention mechanisms, encoder-decoder structures, and positional encoding strategies. In addition, the section outlines how these components have been adapted for image-based applications, establishing the technical groundwork for their use in medical image analysis.

**Figure 2:**
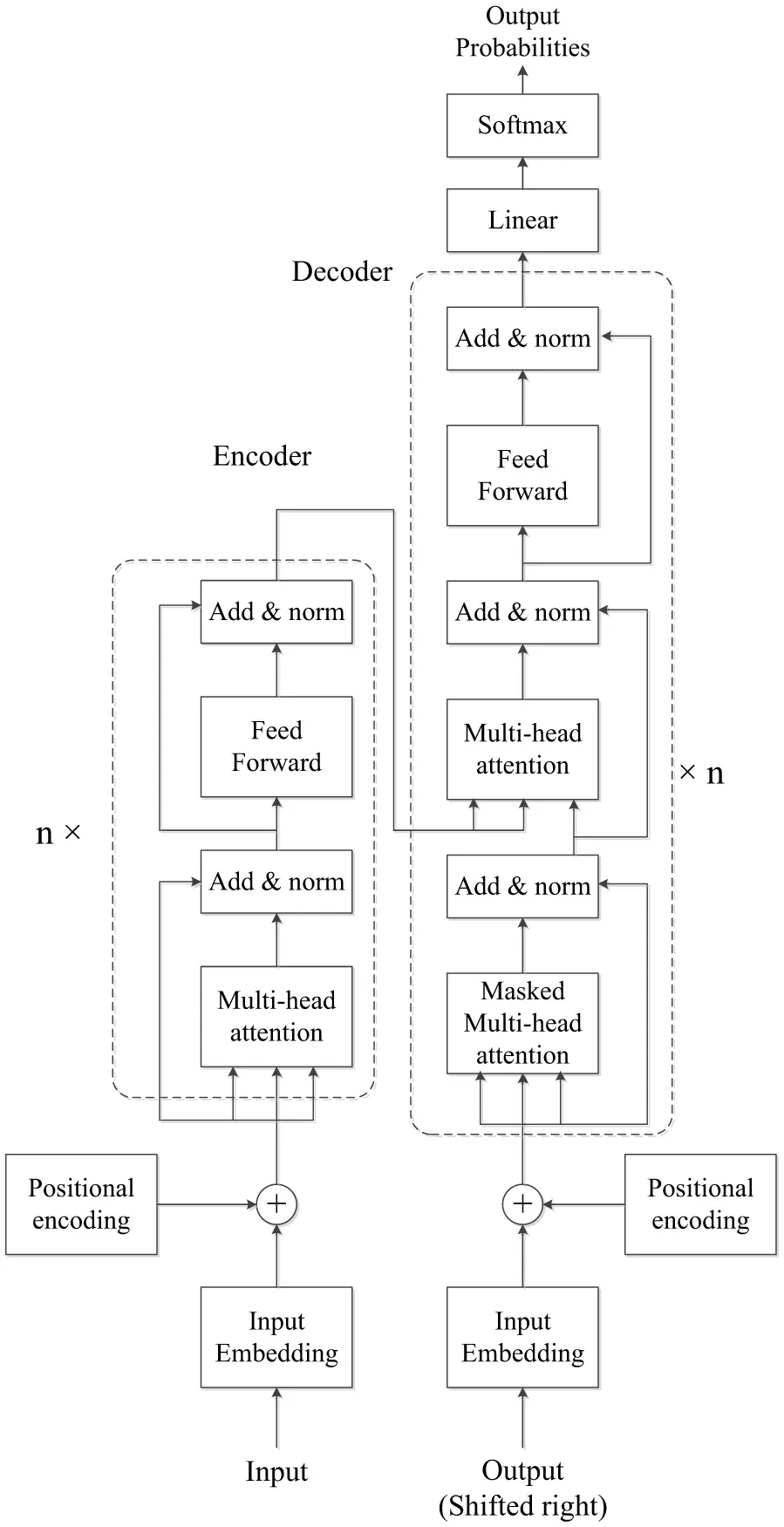
Overview of the Transformer architecture, consisting of an encoder and a decoder.

Figure 2 illustrates the standard Transformer architecture, highlighting the parallel encoder-decoder structure and the flow of information through multi-head attention and feed-forward components. The architecture follows an encoder-decoder framework in which the encoder transforms the input sequence into a continuous latent representation, while the decoder generates the corresponding output sequence. Unlike earlier encoder-decoder models that employ recurrent units, the Transformer applies multi-head self-attention within both encoder and decoder layers. Self-attention mechanisms assign contextual importance to individual input tokens, enabling the encoder to process input representations and the decoder to focus on relevant encoder outputs during generation. The architecture supports the modeling of complex relationships within sequences while maintaining high parallelizability, leading to substantial performance improvements across a range of sequence modeling tasks (Vaswani et al., 2017).

### 3.1 The self-attention mechanism in transformer models

Self-attention is the central mechanism that underpins the functionality of Transformer models (Vaswani et al., 2017). Unlike RNNs, which process sequences sequentially, self-attention allows a model to consider all positions in the input simultaneously. This mechanism computes a weighted representation of each input element by attending to all other elements in the sequence, enabling the model to capture both local and global dependencies (Ansari et al., 2025).

The computation of self-attention is based on three trainable vectors for each input: the query (**Q**), the key (**K**), and the value (**V**) (Vaswani et al., 2017). For a given input sequence, these vectors are derived through linear projections. The attention score between each pair of input tokens is calculated by taking the scaled dot product of their query and key vectors. These scores are normalized using the softmax function to produce attention weights, which are then applied to the value vectors to generate the final output representation (He et al., 2023; Vaswani et al., 2017):


$$\begin{array}{l} \text{Attention}\left(\text{Q},\text{K},\text{V}\right)=\text{softmax}\left(\frac{\text{Q}{\text{K}}^{⊤}}{\sqrt{d_{k}}}\right)\text{V}, \end{array}$$

where *d*_*k*_ denotes the dimensionality of the key vectors, and $\sqrt{d_{k}}$ is used as a scaling factor to prevent gradient instability. This formulation allows the model to assign higher importance to relevant input components and suppress less informative ones during representation learning (Ansari et al., 2025).

To increase the representational capacity, Transformer models typically employ *multi-head attention*. This involves running multiple self-attention mechanisms in parallel, each with distinct learned projections, followed by a concatenation of the outputs. Multi-head attention enables the model to attend to different aspects of the input data simultaneously, enriching its contextual understanding (Zhu and Wang, 2023). The self-attention mechanism not only facilitates parallel computation but also allows the model to dynamically adjust the receptive field based on task-specific requirements. These properties make it highly suitable for complex data types such as text, images, and increasingly, medical imaging modalities where capturing long-range dependencies is essential for accurate interpretation.

### 3.2 Transformer encoder

The encoder in the Transformer architecture, as depicted in Figure 2, consists of a stack of identical layers, each designed to capture contextual relationships across the input sequence. In the base model, six such layers are employed. Each layer comprises two primary components: a multi-head self-attention mechanism and a position-wise feed-forward network. The self-attention mechanism enables the encoder to assess the relevance of different tokens within the sequence when computing each token's representation, facilitating the modeling of both local and global dependencies. To support stable optimization, residual connections are applied around each sub-layer, followed by layer normalization. This design mitigates issues such as vanishing gradients and supports the training of deep architectures. Furthermore, all sub-layer outputs are constrained to a consistent dimensionality—512 in the base configuration—to ensure compatibility with residual operations. The feed-forward component consists of two fully connected layers separated by a Rectified Linear Unit (ReLU) activation function. This transformation is applied independently to each position in the sequence, using the same parameters across positions but distinct weights for each encoder layer. This structure allows the encoder to refine token-level representations while maintaining positional independence during intermediate transformations.

### 3.3 Transformer decoder

As illustrated in Figure 2, the decoder mirrors the structure of the encoder and is composed of six identical layers in the base model. Like the encoder, each decoder layer includes a multi-head self-attention mechanism and a feed-forward network. However, Transformer decoder also introduces a third sub-layer that performs multi-head attention over the encoder's output. The additional layer allows the decoder to align its output generation with the encoded input, effectively integrating source context during sequence prediction. To preserve autoregressive properties during generation, masked self-attention is employed in the decoder's initial sub-layer. This mechanism prevents positions from attending to future tokens, ensuring that each output token is generated based solely on preceding tokens. The decoder also incorporates residual connections and layer normalization after each sub-layer, following the same strategy as the encoder. Similar to the encoder, the decoder adds positional encodings to input embeddings to encode token order. Given that the Transformer architecture lacks inherent recurrence or convolution, these positional encodings are essential for enabling the model to capture sequential structure within the data.

## 4 Vision transformer architectures for image analysis

The success of Transformer architectures in natural language processing has catalyzed extensive research into their adaptation for computer vision tasks. Unlike traditional convolutional models that rely on local receptive fields, ViTs utilize self-attention mechanisms to capture global dependencies across image regions. This paradigm shift has led to the development of several Transformer-based models tailored for visual representation learning, including the detection Transformer (DETR), the ViT, the data-efficient image Transformer (DeiT), and the ST. These architectures vary in their design choices and computational strategies but share a common goal of enhancing visual understanding through attention-driven modeling.

### 4.1 Detection transformer (DETR): end-to-end object detection with attention

DETR represents a foundational shift in object detection frameworks by eliminating the need for complex post-processing components traditionally used in region-based convolutional approaches such as Faster R-CNN (Ren et al., 2015). Introduced by Carion et al. (2020), DETR formulates object detection as a direct set prediction problem, enabling end-to-end optimization without the use of hand-crafted components like region proposal networks or non-maximum suppression.

The architecture integrates a convolutional backbone, typically a ResNet variant, to extract spatial feature maps from input images. These features are then projected into a fixed-length sequence and passed to a standard Transformer encoder-decoder structure. The decoder operates on a fixed set of learned object queries, each of which interacts with the encoded image features through cross-attention layers. The output is a set of predictions corresponding to object class labels and bounding box coordinates (Shehzadi et al., 2025).

A distinguishing component of DETR is its use of bipartite matching for object prediction alignment. During training, the model minimizes a Hungarian loss, which combines classification and bounding box regression losses to ensure a one-to-one correspondence between predicted and ground truth objects. This matching approach resolves common issues in dense detectors such as duplicate predictions and heuristic filtering (Liu F. et al., 2025).

DETR's reliance on global self-attention allows it to model long-range spatial dependencies, which is particularly advantageous in scenarios where contextual information improves detection performance. However, the model is known to require longer training times and exhibits slower convergence compared to conventional methods, especially in detecting small objects (Qi et al., 2022; He et al., 2023).

In the context of medical image analysis, DETR's attention-based formulation offers potential benefits for tasks involving lesion detection, anatomical structure localization, and other spatially sensitive predictions. Its capacity to perform holistic reasoning across an image without region-based heuristics makes it a promising candidate for future adaptations in medical imaging applications.

### 4.2 Vision transformer: patch-based modeling for image recognition

The ViT, introduced by Dosovitskiy et al. (2021), extends the original Transformer architecture to image classification tasks by representing images as sequences of patches rather than pixels or regions processed by convolutional layers. The model takes an input image and divides it into fixed-size non-overlapping patches, each of which is flattened into a one-dimensional vector and linearly projected into an embedding space. These patch embeddings serve as input tokens to the Transformer encoder, allowing the model to process visual data similarly to textual sequences.

ViTs extend the Transformer architecture to image analysis tasks by replacing traditional convolutional operations with self-attention-based processing. Instead of using convolutional layers for hierarchical feature extraction, ViTs divide input images into fixed-size non-overlapping patches, which are treated as a sequence of input tokens. These tokens are then passed through a multi-layer Transformer encoder that leverages self-attention mechanisms to model global relationships across image regions. This approach supports parallel computation and facilitates the learning of long-range dependencies, which are critical for complex visual tasks. To retain spatial information, learnable positional embeddings are added to each patch embedding. Additionally, a special classification token is prepended to the sequence, enabling the model to aggregate global information from all patches during training. The encoded representation of this token is used for final classification (Wu et al., 2024; Elharrouss et al., 2025).

ViT employs the standard Transformer encoder, consisting of multi-head self-attention mechanisms and position-wise feed-forward networks, with residual connections and layer normalization applied after each sub-layer. Unlike CNNs that incorporate inductive biases such as locality and translation equivariance, ViT relies entirely on data-driven learning to capture spatial dependencies. This design allows the model to build flexible representations, but it also increases dependence on large-scale datasets and computational resources for effective training. Experimental results demonstrated that ViT can achieve competitive or superior performance compared to convolutional architectures when trained on large datasets such as ImageNet-21k or JFT-300M. However, its performance tends to decline on smaller datasets, indicating lower data efficiency in scenarios with limited labeled samples (Wu et al., 2024; Elharrouss et al., 2025).

In medical image analysis, ViT shows potential in classification tasks that require modeling of complex spatial relationships across entire images. Applications include disease detection in chest radiographs, lesion classification in dermoscopy images, and cancer diagnosis from histopathology slides. The ability of ViT to model long-range dependencies and global patterns without relying on convolutional structures makes it a valuable foundation for further research in vision-based medical tasks. The ViT architecture is primarily composed of three key components: a patch embedding layer, a Transformer encoder, and a multilayer perceptron (MLP) head for prediction tasks such as classification or regression (Wu et al., 2024; Elharrouss et al., 2025; Jiang et al., 2024).

#### 4.2.1 Patch embedding layer

The patch embedding layer prepares the image for processing by the Transformer encoder. The input image is divided into non-overlapping patches, and each patch is flattened into a one-dimensional vector (Chauhan et al., 2025). These vectors are projected linearly into a lower-dimensional embedding space to form the patch embeddings. This procedure is analogous to word embeddings in natural language processing. To preserve spatial structure, learnable positional embeddings are added to each patch embedding. These embeddings encode the relative or absolute position of each patch within the image, allowing the model to maintain spatial awareness throughout the sequence. The combination of patch and position embeddings forms the input to the encoder (Venkatraman et al., 2025).

#### 4.2.2 Transformer encoder module

Following the embedding stage, the token sequence is passed into the Transformer encoder. This encoder consists of *M* stacked Transformer blocks, where each block includes a multi-head self-attention (MSA) (Alamri and Dutta, 2021) mechanism and a multilayer perceptron (MLP), along with layer normalization and residual connections. The MLP modules typically comprise two fully connected layers with GELU activation functions and maintain a consistent *D*-dimensional output across layers to support residual learning. The encoder is responsible for capturing global contextual dependencies through self-attention and refining feature representations via the MLP. As information flows through successive layers, feature vectors are incrementally enriched, enabling the model to build robust representations of the input. The output corresponding to the prepended classification token (often denoted ${\text{z}}_{0}^{M}$) is extracted as a summary representation of the entire image and passed to the final classification head (Wu et al., 2024; Elharrouss et al., 2025; Jiang et al., 2024).

#### 4.2.3 MLP head module

The MLP head functions as the final prediction component of the ViT architecture (Tolstikhin et al., 2021). It operates on the encoded class token output from the Transformer encoder. First, layer normalization is applied to stabilize the input. A fully connected layer then maps the normalized vector to a space corresponding to the number of output classes. Finally, a softmax activation converts these logits into class probabilities. This module enables the transformation of high-level feature representations into interpretable outputs suitable for classification tasks (Wu et al., 2024; Elharrouss et al., 2025; Jiang et al., 2024).

### 4.3 Data-efficient image transformer: knowledge distillation for resource-constrained learning

The DeiT builds upon the ViT architecture by addressing one of its primary limitations: the need for large-scale datasets and substantial computational resources (Touvron et al., 2021; Singh et al., 2025). While ViT performs competitively when trained on massive datasets, its data inefficiency limits its practical adoption in domains where annotated data is scarce. DeiT introduces a training strategy based on knowledge distillation to improve performance in such scenarios without altering the core architectural structure.

DeiT incorporates a distillation token alongside the standard class token in the Transformer input sequence. This additional token is designed to interact with both the image patches and a teacher model during training. The model is trained with two supervision signals: one from the ground truth labels and another from the teacher model's soft predictions. This dual-guidance approach allows the student model to absorb both explicit and implicit knowledge, improving generalization while maintaining architectural simplicity (Deng et al., 2025).

Unlike traditional CNNs that often rely on inductive biases for generalization under limited data, DeiT leverages the attention mechanism to learn global dependencies directly from smaller datasets (Pacal and Kunduracioglu, 2024). This makes it well-suited for tasks such as image segmentation and classification in data-constrained environments. Additionally, DeiT retains the core advantages of Transformer-based models, including parallel processing and scalability, while substantially reducing the dependence on extensive pre-training.

Through its efficient distillation framework and minimal architectural modifications, DeiT demonstrates that Transformer-based vision models can be trained effectively without access to large-scale datasets. This approach is particularly relevant in medical image analysis, where labeled data is often limited due to annotation cost, privacy constraints, and expert dependency.

### 4.4 Swin transformer: hierarchical vision modeling with shifted window attention

The ST, introduced by (Liu et al. 2021), extends the ViT framework by incorporating a hierarchical architecture and localized self-attention to improve computational efficiency and scalability. While the original ViT applies global self-attention across the entire image, ST partitions the input into non-overlapping windows and restricts attention computation to these localized regions. This approach significantly reduces computational complexity, particularly for high-resolution images (Xiao et al., 2023).

To enable information exchange between windows and capture cross-window dependencies, the framework introduces a mechanism known as shifted window multi-head self-attention (SW-MSA) (Pacal et al., 2024). In this configuration, the partitioned windows are shifted in subsequent layers, allowing overlapping coverage of neighboring regions without incurring the cost of global attention. This alternating use of standard window-based attention (W-MSA) and shifted window attention allows the model to achieve a balance between computational efficiency and representational capacity (Pacal et al., 2024).

The ST is also designed with a hierarchical structure, where feature maps are progressively downsampled across stages. This design enables the extraction of multi-scale feature representations, similar to the pyramidal feature hierarchy used in CNNs. As a result, the ST serves as a flexible backbone for a wide range of vision tasks, including classification, detection, and segmentation.

**Figure 3:**
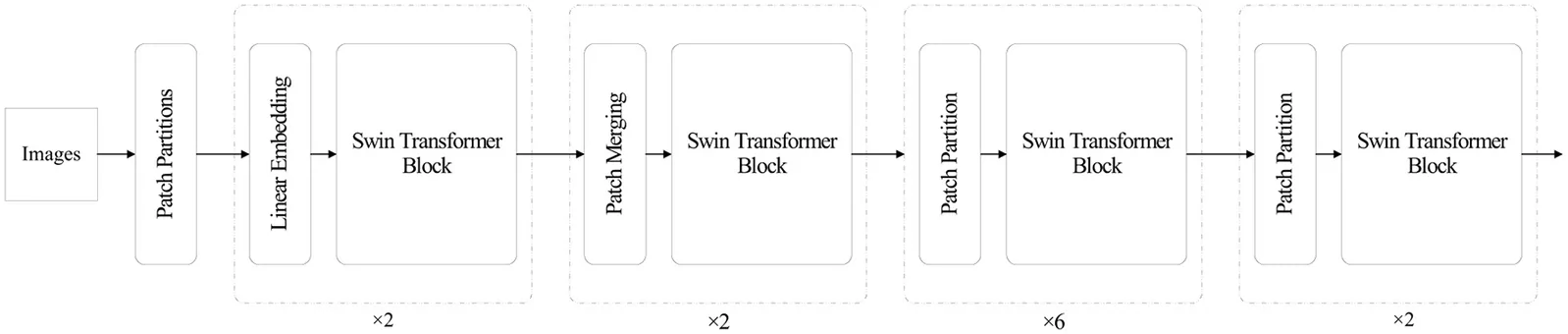
The architectural framework of the Swin Transformer.

**Figure 4:**
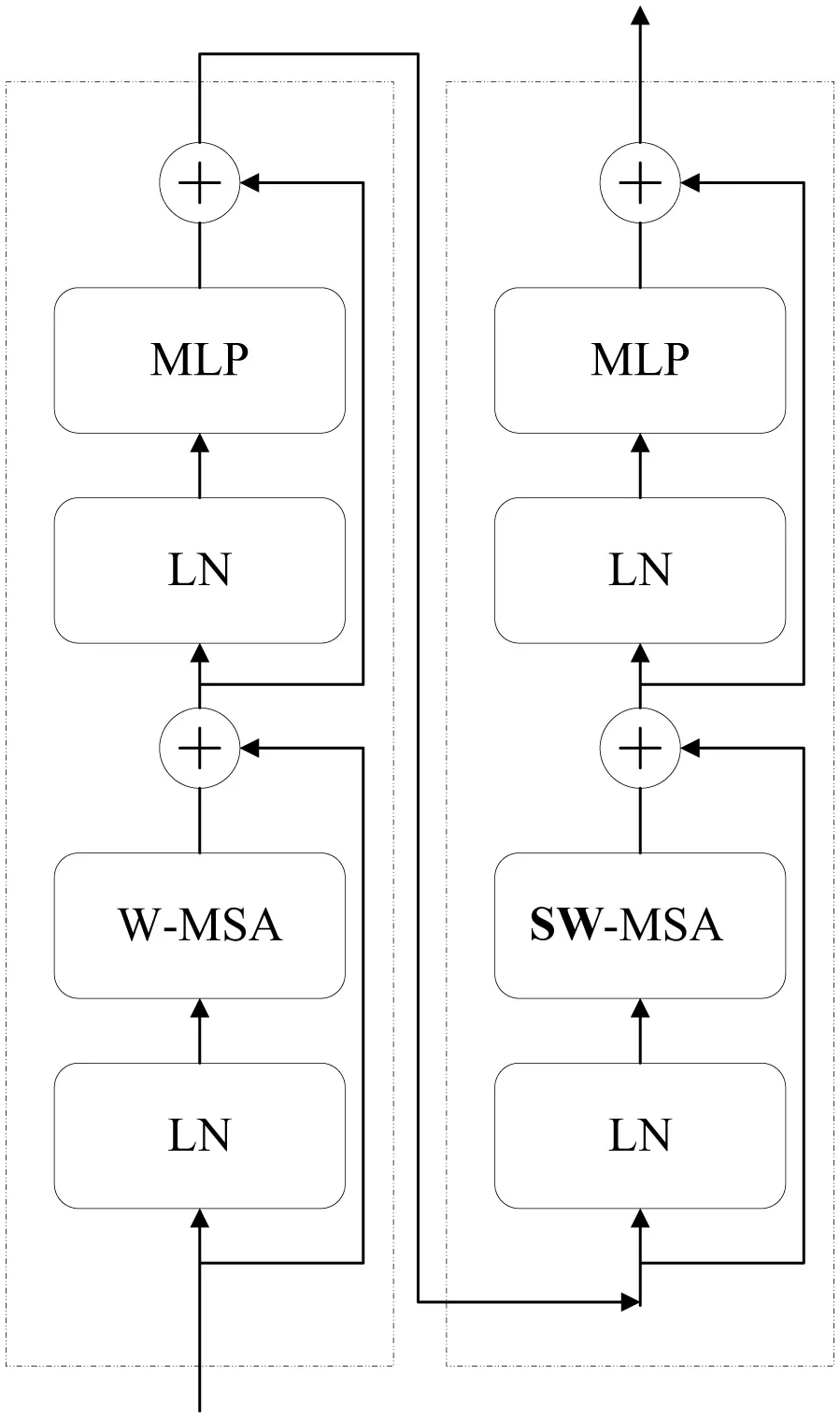
The Swin Transformer block is structured using Window Multi-Head Self-Attention (W-MSA) and Shifted Window Multi-Head Self-Attention (SW-MSA) modules.

The architectural design of ST blocks, as illustrated in Figures 3, 4 (Liu et al., 2021), integrates both local processing and global context modeling over multiple layers. This makes it particularly well-suited for dense prediction tasks, such as semantic segmentation and object localization. In medical image analysis, the ST has demonstrated effectiveness in segmentation applications, where accurate delineation of anatomical structures often requires modeling both local boundaries and global spatial context.

### 4.5 Comparative analysis of transformer architectures for image understanding

The Transformer-based models examined in Section 4.5 represent distinct yet complementary advancements in visual representation learning. While they all inherit the core self-attention mechanism from the original Transformer architecture, they diverge in their structural design, computational strategies, and suitability for specific tasks and data regimes.

DETR reformulates object detection as a set prediction problem, eliminating the need for traditional components such as region proposal networks and non-maximum suppression. Its encoder-decoder structure leverages global self-attention to enable end-to-end training and holistic reasoning across entire images. However, its computational demands and slower convergence, particularly in detecting small objects, limit its efficiency in resource-constrained settings.

ViT introduced a patch-based modeling approach that replaces convolutional operations with multi-head self-attention, enabling global context modeling from the earliest layers. While effective when trained on large-scale datasets, ViT's data inefficiency and lack of inductive biases make it less suitable for domains where labeled data is limited or costly to obtain.

DeiT addresses ViT's limitations by incorporating a knowledge distillation strategy during training. By introducing a distillation token and leveraging supervision from a pre-trained teacher model, DeiT enhances data efficiency without modifying the Transformer architecture. This makes it a viable solution for image classification tasks in settings with limited data availability.

ST builds on ViT's foundation by introducing a hierarchical architecture with window-based self-attention. Through the use of non-overlapping and shifted windows, it balances local processing efficiency with the ability to model global relationships across layers. Its scalability and multi-resolution representation make it suitable for a broad range of vision tasks, including dense prediction problems such as image segmentation.

In the context of medical image analysis, each architecture offers distinct advantages. DETR is suitable for object localization tasks where end-to-end training and interpretability are critical. ViT and DeiT provide strong baselines for image classification, with DeiT being particularly beneficial when training data is scarce. The ST's hierarchical design and efficient attention mechanism make it especially effective for high-resolution medical images and segmentation tasks requiring fine-grained spatial understanding.

## 5 Applications of transformer models in medical image analysis

The integration of Transformer-based architectures into medical image analysis has accelerated in recent years, driven by their ability to model long-range dependencies and contextual relationships across spatial dimensions. These capabilities are particularly valuable in medical domains where diagnostic accuracy often depends on capturing subtle patterns and global anatomical context. Transformer models have been adapted for a wide range of tasks, including segmentation, classification, detection, and registration, across various imaging modalities such as MRI, CT, histopathology, and ultrasound. Section 5 surveys recent applications of Transformers in medical imaging, highlighting model adaptations, performance trends, and domain-specific considerations.

The application of Transformer-based architectures in medical image analysis can be examined across several clinically and methodologically distinct task families, including segmentation, classification, detection/localization, reconstruction, and image registration. In many real-world medical imaging workflows, segmentation is a foundational step because the delineation of organs, lesions, tumors, vessels, or pathological regions often supports downstream diagnosis, classification, prognosis, treatment planning, surgical navigation, radiotherapy guidance, and longitudinal disease monitoring. For this reason, the application-level synthesis in this review discusses segmentation before classification, followed by reconstruction and registration. This organization provides a more clinically grounded flow while also preserving the methodological distinction between dense prediction, image-level prediction, image recovery, and spatial alignment tasks.

Although these tasks all benefit from the ability of transformers to model contextual relationships and spatial dependencies, their methodological requirements are not the same. In segmentation, the combination of accurate boundary information with anatomical context is more important; in classification, the main goal is to extract differential representations at the image or region level; in reconstruction, image quality, preservation of fine structures, and reduction of artifacts are the focus; and in image registration, modeling of spatial correspondences between fixed and moving images plays a central role. Therefore, in Section 5, the reviewed studies are organized not by repeating a general logic about CNNs and Transformers, but by the specific needs of each task, imaging modality, architectural design, and type of functional evidence.

### 5.1 Application of the proposed taxonomy in organizing studies

To avoid a scattered and study-by-study presentation of the sources, the studies reviewed are organized according to a three-axis taxonomy. The first axis is the imaging modality, which includes CT, MRI, ultrasound, X-ray images, retinal images, dermoscopy, histopathology, and related biomedical imaging data. The second axis is the type of analytical task, which groups the studies into groups such as segmentation, classification, detection/localization, reconstruction, and image registration. The third axis is the methodological design and model architecture, which includes pure Transformer architectures, Vision Transformers, Swin Transformers, DETR-based models, CNN–Transformer hybrid models, cross-attention-based methods, Transformer-based generative models, foundation or promptable models, and transformer-alternative or non-transformer comparator models such as Mamba, KAN, and nnU-Net. To clarify this structure, Table 4 maps the reviewed studies according to the three main axes of the proposed taxonomy, namely imaging modality, task type, and methodological design. This table serves as an organizing framework for the subsequent sections and shows how each study relates to the paper's overall synthesis of the capabilities, limitations, and trends of transformer-based architectures in medical image analysis.

**Table 4:** Mapping of reviewed studies according to the proposed three-axis taxonomy.

Task type	Imaging modality	Architecture family	Methodological design	Role in the review synthesis
Segmentation	CT, MRI, ultrasound, microscopy, endoscopy, and multi-organ datasets	U-Net–Transformer hybrids	Integration of transformer encoders or decoders into U-Net-like structures, cross-attention between CNN features and transformer tokens, and hybrid local–global segmentation learning, as exemplified by TransUNet and related models (Chen J. et al., 2024).	Used to examine how transformer modules complement convolutional encoders and decoders in dense prediction tasks requiring both boundary precision and long-range contextual modeling.
Segmentation	Brain, abdominal organs, skin lesions, vessels, tumors, and multimodal datasets	Transformer alternatives and comparison models	Mamba-based, KAN-based, and strong CNN-based baselines such as nnU-Net are treated as contextual comparators rather than core transformer models (Liu J. et al., 2025; Li C. et al., 2025).	Used to prevent overgeneralized claims of transformer superiority and to distinguish transformer-based architectures from alternative long-range modeling frameworks and non-transformer baselines.
Classification	MRI, CT, X-ray, dermoscopy, fundus imaging, histopathology	ViT-based and medical ViT variants	Patch-based image tokenization, global self-attention, self-supervised learning, token perturbation, and transformer-based feature extraction, as reported in studies such as MedViT, BOLT, DBCvT, Eff-CTM, and MedViTV2 (Nejati Manzari et al., 2023; Li Y. et al., 2024; Li J. et al., 2024; Liu S. et al., 2024; Nejati Manzari et al., 2026).	Used to analyze how transformer-based classifiers capture global image context, improve representation learning, and address data scarcity in diagnostic classification tasks.
Classification	Dermoscopy, histopathology, ultrasound, and multimodal clinical images	Swin Transformer, CrossViT, and hybrid CNN–Transformer models	Hierarchical attention, shifted-window self-attention, multi-scale encoding, feature selection, and hybrid local–global feature fusion, as illustrated by Swin-based, CrossViT-based, and optimization-assisted classification models (Kumar et al., 2025; Alqahtani et al., 2025; Dahou et al., 2025).	Used to compare transformer-centered classification models across heterogeneous datasets and to highlight task-dependent differences in reported diagnostic performance.
Detection and localization	CT, X-ray, ultrasound, renal imaging, and lesion-centered datasets	DETR-based, YOLO–Swin, and transformer-enhanced detection models	Object-query learning, transformer-based global context modeling, feature pyramid enhancement, and hybrid detection pipelines combining convolutional backbones with attention-based modules (Pan et al., 2024).	Used to assess how transformer-based attention improves lesion localization, object-level reasoning, and contextual detection in medical images.
Reconstruction and super-resolution	MRI, cardiac cine MRI, PET, CBCT, DCE-MRI, and multi-coil MRI	Diffusion Transformers, Transformer-GANs, Swin-GANs, and dual-domain transformer models	Transformer-based latent denoising, cross-domain attention, image–frequency fusion, adversarial reconstruction, and sparse-view or low-dose reconstruction strategies (Tu et al., 2025; Zhang et al., 2023; Lyu et al., 2023; Wang Y. et al., 2024; Wang et al., 2023; Chen et al., 2025; Lin et al., 2024; Yuan et al., 2025; Zhang et al., 2025a).	Used to synthesize evidence on the role of transformers in recovering global anatomical structure, reducing artifacts, and improving image quality under accelerated, low-dose, or undersampled acquisition settings.
Reconstruction, restoration, and image synthesis	Fundus imaging, magnetic particle imaging, ECT, MRI, and CT	Vision Transformer, spectral transformer, residual transformer, and hybrid reconstruction networks	Spectral reconstruction, system matrix recovery, slice-to-volume reconstruction, spatial–frequency fusion, and transformer-guided restoration (Liu J. et al., 2024; M.khair et al., 2026; Yang T. et al., 2025).	Used to show that transformer-based modeling is not limited to diagnostic classification, but also supports image restoration, modality recovery, and computational imaging tasks.
Registration	Brain MRI, CT–MRI, PET–MRI, radiotherapy CT/CBCT, and multimodal anatomical images	ViT-based, symmetric transformer, TransMorph-like, and cross-attention registration models	Long-range correspondence modeling, moving–fixed image feature interaction, affine and deformable transformation learning, topology-preserving deformation fields, and unsupervised similarity-based optimization (Ma et al., 2022).	Used to separate registration studies from segmentation and classification tasks and to emphasize that rigid, affine, and deformable registration require different evaluation metrics and should not be directly merged.
Cross-task and clinical-readiness analysis	Multiple modalities and multi-center datasets	Foundation models, hybrid models, and non-transformer comparators	Large-scale pre-training, general-purpose segmentation or registration backbones, uncertainty-aware designs, external validation, code availability, and computational reporting.	Used to connect the taxonomy to clinical translation by identifying which model families provide evidence of generalizability, reproducibility, deployment potential, or limitations under dataset shift.

Within this framework, each study is interpreted not as an isolated contribution, but according to its modality, task objective, architectural family, validation setting, and comparative context. This structure helps prevent direct over-comparison across heterogeneous datasets and evaluation protocols. For example, Swin Transformer-based models are more prominent in tasks requiring hierarchical processing and high resolution, such as segmentation and pathology image analysis, whereas ViT- and DeiT-based models are more commonly used in medical image-level classification. Cross-attention and hybrid architectures are more commonly observed in reconstruction and registration, where relationships across domains, modalities, or spatial coordinate systems are central to the task. The taxonomy therefore supports a more clinically and methodologically grounded interpretation of reported performance. This three-axis mapping also prevents a direct and simplistic comparison of heterogeneous studies and suggests that the reported performance of each method should be interpreted in the context of the modality, task, dataset, and architectural design of the same study.

### 5.2 Medical image segmentation

Medical image segmentation occupies a central position in many clinical imaging workflows because accurate delineation of organs, lesions, tumors, vessels, or pathological regions often provides the spatial foundation for downstream classification, quantification, treatment planning, radiotherapy guidance, surgical navigation, and longitudinal disease monitoring. In medical image segmentation, the main goal is to accurately determine the boundaries and regions of organs, lesions, tumors, vascular structures, or other clinically important areas. Unlike classification, which usually outputs at the image or class level, segmentation requires dense predictions at the pixel or voxel level, and therefore local accuracy, preservation of boundary details, and understanding of the anatomical context are simultaneously important. Transformer-based studies in this area have often attempted to employ encoder-decoder architectures, U-Net-like structures, attention-gated modules, Swin Transformers, and hybrid models to combine local and contextual information. In this subsection, studies are reviewed based on modality, target structure, type of architecture, and level of functional evidence.

In medical image segmentation, the proposed classification shows that the differences between studies are not limited to the type of model but also depend on the imaging modality, the nature of the target structure, and the type of architectural design. For example, segmentation of lesions, organs, tumors, or vascular structures in CT, MRI, ultrasound, and histological images requires different strategies to combine local and global context information.

Prior work in medical image segmentation has increasingly explored hybrid architectures to leverage the complementary strengths of CNNs and Transformer networks. CNNs excel at capturing local spatial features and hierarchical representations, while Transformers are adept at modeling long-range dependencies and global context within images.

#### 5.2.1 Foundation models and emerging architectures

One of the major recent trends in medical image analysis is the move from single-task, single-modality models to foundation, promptable, and multi-objective models. This trend is particularly prominent in medical image segmentation and computational pathology, as classical models are usually trained for a specific organ, modality, or dataset and suffer from performance degradation when faced with modality changes, imaging protocol changes, or domain differences. In this context, MedSAM is one of the most important efforts to adapt the Segment Anything Model to medical images. This model was developed with the aim of providing a general framework for medical segmentation and, rather than being limited to a specific organ or modality, attempts to provide promptable segmentation capabilities across a wide range of medical images. The importance of MedSAM is that it demonstrates that fundamental vision models, when trained or tuned appropriately on medical data, can move toward more general and less task-dependent segmentation. However, the use of such models still comes with challenges such as prompt type dependency, the need for external evaluation, performance differences between modalities, and the necessity of clinical validation in real-world scenarios (Ma et al., 2024a).

In addition to promptable models, state-space modeling architectures have also emerged as important alternatives to transformers in medical image segmentation. U-Mamba is a prime example of this trend, which combines the U-shaped structure and Mamba modules to provide the advantage of modeling long-range dependencies with linear computational complexity. Unlike classical transformers, where the attention computation usually becomes expensive with increasing image size or data volume, Mamba-based models can be an efficient option for volumetric data and high-resolution medical images. Therefore, U-Mamba is not classified as a pure transformer model in this review, but rather as an alternative transformer and long-range modeling model. This distinction is important, because comparing U-Mamba with models such as UNETR, Swin-UNETR, nnU-Net, or other hybrid CNN-Transformer models should be done with respect to differences in architecture, computational cost, validation protocol and data type (Ma et al., 2024b).

In addition to general segmentation, foundation models in computational pathology are also rapidly becoming an important direction in medical image analysis. As a general foundational model for pathology images, UNI focuses on learning transferable representations from whole-slide images and can be used for various tasks such as classification, retrieval, clustering, and histological analysis. On the other hand, CONCH, focusing on joint image and language modeling, attempts to strengthen the connection between histological images and text/linguistic information for tasks such as classification, captioning, and image-text retrieval. The addition of these models to the present review demonstrates that the research trend in the field of transformers and foundational medical models is not limited to improving classification or segmentation architectures, but is moving toward more general, multi-task, multimodal, and large-scale transferable models (Chen R. J. et al., 2024; Lu et al., 2024).

To further clarify the position of recent foundation models and transformer alternatives, Table 5 summarizes MedSAM, U-Mamba, UNI, and CONCH according to their model category, target domain, methodological contribution, and relevance to medical image analysis. Despite the importance of these new models, the existing evidence should be interpreted with caution. Many fundamental medical models have not yet reached full maturity in terms of multicenter evaluation, external validation, calibration, uncertainty analysis, reader evaluation, and examination of real-world impact on clinical workflow. Therefore, in this review, MedSAM, U-Mamba, UNI, and CONCH are analyzed not as evidence for the definitive superiority of one architectural family, but as examples of emerging trends in generalizable, promptable, multimodal, and scalable models.

**Table 5:** Recent foundation models and transformer alternatives relevant to medical image analysis.

Model/study	Model category	Primary domain	Methodological contribution	Relevance to medical image analysis	Main limitation
MedSAM (Ma et al., 2024a)	Medical foundation model for promptable segmentation	Multimodal medical image segmentation	Adapts the Segment Anything Model to medical imaging through large-scale medical segmentation data and prompt-based segmentation using box or point guidance.	Provides a general-purpose segmentation framework across diverse medical modalities and reduces dependence on task-specific segmentation model design.	Performance remains prompt-dependent and requires further external, multi-center, and clinically oriented validation.
U-Mamba (Ma et al., 2024b)	Transformer alternative based on state-space modeling	2D and 3D biomedical image segmentation	Combines a U-shaped architecture with Mamba state-space modules to capture local features and long-range dependencies with linear computational complexity.	Offers an efficient alternative to attention-based Transformers for high-resolution and volumetric medical segmentation tasks.	It is not a pure Transformer model and should be interpreted as a long-range modeling alternative rather than a transformer-based architecture.
UNI (Chen R. J. et al., 2024)	Pathology foundation model	Computational pathology and whole-slide image analysis	Learns transferable histopathology representations from large-scale H&E-stained whole-slide images using self-supervised ViT-based representation learning.	Supports multiple downstream pathology tasks, including tissue classification, retrieval, region-level analysis, and representation transfer.	Not primarily designed as a segmentation-specific model; downstream segmentation requires adaptation or coupling with task-specific heads.
CONCH (Lu et al., 2024)	Vision–language pathology foundation model	Computational pathology and image–text learning	Uses contrastive image–text pre-training to align histopathology image representations with biomedical language.	Enables retrieval, classification, captioning, and more interpretable pathology analysis through vision–language alignment.	Not a standalone segmentation model; pixel-level or region-level segmentation requires additional adaptation.

The reviewed segmentation approaches are organized into four architectural categories: transformer-based segmentation models, hybrid CNN–Transformer models, transformer alternatives, and non-transformer comparative models.

#### 5.2.2 Transformer models

The challenges of manual liver tumor segmentation, labor-intensive and prone to variability, was tackled in Sivanagaraju et al. (2025). Sivanagaraju et al. emphasized the necessity for automated approaches, especially given the difficulty in segmenting liver lesions in MRI images due to variations in appearance. The authors proposed a framework employing Transformer network. The presented framework initially starts by enhancing images quality leveraging a probabilistic fused Wiener filter (PFWF). Afterwards, robust features with a convolutional Res2Net Model (CRes2Net) are extracted. The main part of the framework relies on a multi-level dual aspect self-attention guided transformer (MDA-SGT), which performs the liver tumor segmentation. By utilizing multi-level dual aspect self-attention, the MDA-SGT effectively captures long-range dependencies and leverages rich semantic information from high-level feature maps, which are crucial for accurate segmentation. The model's performance was evaluated on the LiverHCC dataset. It is asserted that the technique achieves a DSC of 97.28%, along with impressive IoU of 94.7%, and specificity of 99.83% scores, demonstrating a significant advancement in automated liver tumor segmentation from MRI images.

The work in Cai et al. (2025) aimed to address several challenges in deep learning-based medical image segmentation. The challenges include difficulties with heterogeneous objects, neglecting frequency-domain features, and limited generalization across diverse datasets. The authors proposed a medical segmentation network based on a dual-domain deformable transformer and a multi-task adaptive learner. The model uses a hybrid architecture comprising a spatial domain transformer, a frequency domain analyzer transformer, and a multi-task adaptive learning module for efficient processing of diverse medical imaging modalities. Their key contributions include an innovative dual-domain deformable attention mechanism that combines deformable attention with a frequency domain Transformer in order to better learn complex heterogeneous objects. In addition, a dynamic frequency analyzer using the discrete Fourier transform (DFT) is proposed to selectively retain crucial low-frequency or high-frequency information while mitigating feature loss. Eventually, a multi-task adaptive learner, integrating multi-scale information and spatial attention, is introduced to improve the adaptability and generalization of the framework across different datasets. Based on the experimental studies and evaluation of the presented algorithm on 13 datasets across five modalities, it is reported that the method outperforms existing state-of-the-art approaches.

In response to the lack of comprehensive foundational models for medical 3D volume segmentation, the SegVol in Du et al. (2024) was introduced which presents a 3D foundation model for medical volumetric image segmentation. The model is capable of segmenting over 200 anatomical structures using spatial and semantic prompts and is trained on 90,000 unlabeled CT images and 6,000 labeled images. The SegVol architecture consists of four main modules: a 3D ViT image encoder, a CLIP-based text encoder, a command encoder, and a mask decoder. It significantly improves the accuracy of the region of interest (ROI) by using an innovative Zoom-out–Zoom-in mechanism, while reducing computational cost. In experiments on 22 volume segmentation tasks from 25 public databases (including AMOS22, BTCV, SegTHOR, and ULS23), the SegVol model outperforms competitors in 19 cases and achieves an average Dice score of 0.8593, which is an improvement of 19.25% and 4.28% over the SAM-MED2D and SAM-MED3D models, respectively. In terms of performance, SegVol, with 181M parameters and 6.7 × 10^11^ MACs, has an inference time of 0.32 seconds per volume, while its accuracy is higher than all SAM-like models. Furthermore, the results of the ablation studies show that combining semantic and spatial commands (such as BBox + Text) increases the average accuracy by 5.85% in Dice, and the Zoom-out–Zoom-in mechanism increases the computation speed by up to 17 times compared to the Sliding Window algorithm. Qualitatively, SegVol's ability to resolve semantic ambiguity using Text Prompt is outstanding; for example, the text command “liver” accurately distinguishes between “liver tumor,” “liver vessels” and “liver tissue.” On the other hand, the model without specific training on MRI has also shown the ability to generalize to other imaging modalities. SegVol is the first comprehensive foundational model for interactive and general 3D segmentation in medicine and provides a template for the next generation of multi-command models based on language and space in volumetric image analysis.

The work in Wu J. et al. (2025) proposed the medical segment anything model adapter (Med-SA), aiming to present an innovative approach to adapt the segment anything model (SAM) model to the field of medical image segmentation. While the SAM model has been proposed as a powerful base model in computer vision, its performance on medical data suffers significantly due to domain differences and limitations in learning 3D volumetric features. Med-SA addresses this challenge by designing a lightweight, adapter-based tuning mechanism that updates only about 2% of the SAM parameters (approximately 13 million parameters), while the backbone, ViT, of the original model remains unchanged. The architecture consists of two key components: first, the SD-Trans (Space–Depth Transpose) module, which provides the ability to process 3D images (CT and MRI) by reconfiguring the spatial and depth dimensions in the 2D SAM model, thereby effectively modeling inter-slice dependency; second, the HyP-Adpt (Hyper-Prompting Adapter) module, which transforms the model into an interactive and adaptive model by learning weights conditioned by prompts (such as clicks, boxes, or initial masks). This design allows Med-SA to adjust the system behavior to different imaging modalities and structures without the need for complete retraining. In comprehensive evaluations on 17 datasets across modalities including CT, MRI, ultrasound, fundus, and dermoscopy, Med-SA has demonstrated superior performance over reference models including MedSAM, nnU-Net, Swin-UNETR, TransUNet, and CoTr. For example, on the BTCV dataset (abdominal organs in 3D CT), the model achieved Dice = 89.8% and HD95 = 2.18 mm, representing a 2.9% improvement in Dice and a 9–11 mm reduction in the marginal error (HD95) over Swin-UNETR. Cross-modality testing results also demonstrate high stability and generalizability across retinal, thyroid, skin lesions, and brain tumor data. The authors mentioned that the Med-SA, by providing a 3D, interactive, parameter-driven optimization solution, demonstrates that general-purpose models such as SAM can be adapted to specialized medical applications with minimal tuning costs. This framework is an effective step toward developing cost-effective, multi-domain models for accurate and interpretable medical image segmentation.

#### 5.2.3 Hybrid CNN-transformer models

Despite transformers' remarkable success in medical image segmentation, convolutional network-based models such as U-Net face limitations in modeling long-range dependencies and simultaneously extracting local and global contexts. Therefore, recent researches have focused on emlpoying Transformer-based structures, especially in the form of encoder-decoder architectures. One of the prominent studies in this field is TransUNet, which was first introduced in 2021 and was able to add the transformer self-attention mechanism to the core of the U-Net architecture. The framework introduced in Chen J. et al. (2024), the versatile framework of TransUNet, by designing transformer encoder and decoder modules, is responsible for extracting global context from CNN feature maps and improving proposed regions through cross-attention between proposed features and U-Net, respectively. The main innovation of this model lies in its high flexibility; in such a way that the aforementioned modules can be integrated independently or in combination into the U-Net structure (including encoder-only, decoder-only, and encoder+decoder modes). The experimental results of this research show that the transformer encoder performs significantly in modeling the interactions between multiple abdominal organs, while the decoder is efficient in improving the detection of small targets such as tumors. The study reported that the performance of TransUNet in segmentation of multi-organ, pancreatic tumor and hepatic vessels, with an average Dice index increase of more than 4.3% compared to nn-UNet, is a testament to the effectiveness of the synergistic approach of CNN and transformer in the field of medical vision. In addition, the publication of 2D and 3D versions of the model paves the way for future research toward the localization of attention-based architectures in medical image data analysis.

The increasing prominence of transformer-based models in CT image segmentation is highlighted in Lv et al. (2025), noting the ST's limitations in capturing fine-grained details and its tendency to focus on local features over global context. In order to address these shortcomings, Lv et al. introduced the innovative U-shaped hybrid self-guided Transformer network (SG-UNet). The algorithm refines the self-attention mechanism by integrating hybrid attention, which employes adaptive fine-grained global self-attention to capture low-level details and guide token assignment, with self-guided attention. The technique dynamically reallocates tokens to prioritize target regions and reduce computation on non-target areas. This synergy pave the way for autonomous refinement of saliency maps and token reassignment based on regional importance. The authors further mentioned that the empirical results on the Synapse and lung datasets demonstrate the superior segmentation performance of SG-UNet. It is also claime that the model achieve impressive DSC and HD scores compared to existing methods, underscoring the effectiveness of the algorithm in CT image segmentation tasks.

A transformer-based framework for tooth semantic segmentation in dental cone beam computed tomography (CBCT) images namely Trans-VNet is presented in Wang C. et al. (2024). The model aims to overcome challenges like noise and sparse data. The Trans-VNet employs the V-Net architecture alongside transformer modules. A key innovation is a patch-based region of interest extraction strategy to focus on teeth and reduce metal artifact influence, unlike Unetr++, V-Net, and nn-UNet. Additionally, a cross-attention module is incorporated to enhance the capture of comprehensive contextual information, overcoming uneven data distribution. By presenting experimental results on a dataset demonstrate, the authors highlighted the Trans-VNet's performance and claimed that the model achieves a Dice similarity coefficient (DSC) of 96.44% and an Intersection over Union (IoU) of 95.17% compared to existing techniques. The authors reported that the framework illustrates significant potential for advancing tooth segmentation and they suggested that future work to focus on handling metal artifacts in datasets and optimizing for broader dental applications.

The ResTransUNet model, as introduced by Anwar et al. (2025), builds upon this trend by proposing a novel combination of a ResNet50 encoder, a standalone Transformer block, and a UNet-style decoder. This integration aims to overcome the limitations of purely convolutional or transformer-based approaches in accurately segmenting complex medical structures like liver tumors. By incorporating a Transformer block within the UNet architecture, ResTransUNet seeks to enhance the model's ability to understand global relationships within the CT and liver images, thereby improving segmentation accuracy and robustness, particularly in challenging cases with subtle tumor boundaries or variations in organ shape and appearance. The authors claimed that the reported performance on benchmark datasets like LiTS and 3D-IRCADb-01 underscores the potential of their hybrid designs in advancing the state-of-the-art in medical image segmentation.

The study in Zhang et al. (2025b) addressed the challenges faced by CNNs in segmenting breast ultrasound images. These challenge in particular are faced in capturing long-range dependencies for lesions with similar intensities, irregular shapes, and blurred boundaries. To overcome these limitations, the authors proposed FET-UNet, a hybrid model integrating CNNs equipped with ResNet34 blocks and STs within a UNet-like architecture. FET-UNet employs parallel branches for feature extraction, which are then fused exploiting an advanced feature aggregation module (AFAM) to effectively combine local details and global context. The FET-UNet leverages the complementary strengths of CNNs for local feature extraction and Transformers for global context modeling through parallel processing and a novel feature aggregation strategy, leading to improved breast ultrasound image segmentation. A multi-scale upsampling mechanism in the decoder ensures precise segmentation outputs. It is asserted that evaluations on the BUSI, UDIAT, and BLUI datasets depict FET-UNet's good performance compared to state-of-the-art architectures, achieving high Dice coefficients across all datasets.

Guo et al. (2024) presented the uncertainty-guided CNN-Transformer network (UCTNet) framework introducing a new generation of hybrid CNN and Transformer networks for medical image segmentation, with a primary focus on reducing the functional overlap between the convolutional and transformer modules. The authors argue that in many previous hybrid models, such as TransUNet, nnFormer, and PHTrans, both CNN and Transformer parts operate without task differentiation, leading to feature duplication, functional overlap, and loss of convergence. To address this problem, UCTNet provides an innovative mechanism called uncertainty-guided vision Transformer (UgViT), which focuses only on regions with high uncertainty in the CNN output and, through uncertainty maps and fuzzy boundary removal, identifies patches that require global dependencies and performs self-attention only on them. The UCTNet architecture consists of a pure convolutional encoder and a hybrid decoder consisting of UCTBlock blocks, in which in each block, the CNN output is first processed to extract high-uncertainty regions, and then these regions are fed into the UgViT module to form long-range dependencies in limited and targeted spaces. With this design, the model simultaneously benefits from the spatial accuracy of CNN and the abstraction ability of Transformer, and also reduces its computational complexity from quadratic to linear. Experimental results on three reference datasets Synapse (multi-organ CT), ACDC (cardiac MRI), and ISIC2018 (skin images) show that UCTNet achieves Dice = 89.44%, 92.91%, and 91.15%, respectively, outperforming advanced models such as nnU-Net, D-Former, H2Former, and ConvFormer in all three cases. Specifically, in Synapse, the model achieved a 2.45% improvement over nnU-Net and a 0.51% increase in average Dice in ACDC. In addition, UgViT was tested as a plug-and-play module in other models and in both cases (CE-Net and H2Former) it increased the accuracy and reduced the Hausdorff distance.

Hua et al. (2024) addressed the challenge of high parameter counts in 3D CNN-Transformer hybrid models for medical image segmentation, which hinders training and deployment. They propose a lightweight 3D multi-target semantic segmentation model designed to capture complex features efficiently. To enhance contextual texture connections and detailed feature expression, they introduce a multi-scale and multi-angle feature interaction (MMI) module. To mitigate attention collapse in Transformers, they use local features as dynamic parameters to interact with global features, enabling dynamic grouping and learning of critical global features, thus improving detailed feature learning. Their model achieves a parameter count of only 9.63M. Extensive experiments on ACDC, Brats2018, and a private temporal bone CT dataset demonstrate the competitiveness of their lightweight model compared to state-of-the-art 3D medical image segmentation techniques. The architecture involves initial feature extraction, an encoder with convolutional modules and downsampling, the MMI module at skip connections, a bottleneck with globallocal feature interaction, and a decoder with residual structures leading to the segmentation output.

The study in Chen H. et al. (2024) presented CTFF-Net, a network for segmenting Parkinson's disease (PD)-related deep gray matter (DGM) nuclei in brain MRI. The model is claimed to address challenges posed by degenerative changes and low contrast. The encoder utilizes alternating CNN and Transformer layers to capture both local and global features, which are then fused by a cascaded channel-spatial-wise fusion (CCSF) block for precise segmentation. The decoder incorporates a symmetrical boundary attention module (SymBA), utilizing signed distance maps to refine boundaries based on the brain's symmetry. A dynamic adaptive weighted Dice loss further enhances sensitivity to small structures. The authors declared that the model demonstrates excellent performance on multi-center and public datasets and shows good generalizability. Volumetric analysis of the results indicates significant differences between healthy controls and PD patients. CTFF-Net holds promise for aiding in the accurate and rapid diagnosis of PD by providing precise DGM nuclei segmentation.

In Dai et al. (2024), the I^2^U-Net network was introduced as an advanced two-path architecture for medical image segmentation. Aiming to overcome the limitations of classical U-Nets in incomplete use of hierarchical information, this model designs two parallel paths: the first path to extract image features, and the second path to store and reuse hidden states as learnable matrices with zero initial value, which creates a mechanism similar to an open RNN. As a result, the I^2^U-Net is able to model temporal relationships between layers and reproduce and re-examine historical information throughout the depth of the network. Two key modules play a fundamental role in this architecture: first, the multi-functional information interaction (MFII) module, which models three types of cross-interactions, between paths, between layers, and between path-and-layer, in a unified design; second, the holistic information fusion and augmentation (HIFA) module, which combines spatial pyramid pooling and multiscale Etruscan convolutions in a non-local block to simultaneously extract low-frequency (overall structure) and high-frequency (edge and texture) features. In terms of performance, I^2^U-Net has shown significant superiority over state-of-the-art methods such as H-Net, Swin-UNet, and 3D TransFuse in four challenging tasks, including skin lesion, polyp, brain tumor, and abdominal organ segmentation. For example, on the ISIC2018 dataset, the IoU index increased from 81.23% in U-Net to 83.66% and from 88.23% to 90.10% in Dice, and in the off-domain evaluation on PH2, a 2.22% improvement in IoU was reported over the best available two-path model. In the 3D version, the 3D I^2^U-Net achieved average Dice of 72.30% and 83.98% on the MSD and Synapse datasets, respectively, which is 1.0%–1.5% higher than TransBTS and TransFuse, while having a moderate computational cost of 80 M parameters and 175 GFLOPs. Qualitatively, I^2^U-Net detects irregularly shaped, low-contrast lesion boundaries with higher resolution and is trained with a faster convergence rate (70 epochs vs. 250) than competing models. These results suggest that hierarchical interaction and intra-model information reuse can be an effective alternative to increasing depth or adding attention-heavy blocks. I^2^U-Net has taken an important step toward designing efficient two-way architectures for 2D and 3D medical image segmentation by balancing local detail reproduction and global semantic abstraction.

Liu H. et al. (2024) aimed to address the limitations of existing 2D and 3D methods in lung tumor segmentation from CT scans, particularly their inability to effectively combine local convolutional patterns with global Transformer dependencies and their neglect of crucial boundary information. To tackle these issues, they propose BGHNet, a 3D boundary-guided hybrid network. BGHNet introduces a hybrid local-global context aggregation (HLGCA) module in the encoder, featuring parallel convolution (VPConvNeXt) and Transformer (VCSwin-Transformer) branches to capture both local and global contexts. In the decoder, a boundary-guided feature refinement (BGFR) module explicitly uses boundary information to refine multistage decoding features. The BGFR module employs an auxiliary boundary detection task and a position-aware feature fusion (PAFF) method to integrate boundary features with region features. Extensive experiments on the HUSTLung and MSD-Lung datasets demonstrate that BGHNet outperforms state-of-the-art methods and exhibits strong generalization across different CT scan types. The architecture strategically uses initial convolutional layers for efficient feature extraction followed by HLGCA blocks. Deep supervision with multi-scale loss is also incorporated in both segmentation and boundary decoders to handle tumor size variations.

In a work presented in Iqbal et al. (2025), the TBConvL-Net model is introduced as a hybrid deep learning framework for medical image segmentation. By combining the capabilities of convolutional networks, i.e., CNN, in extracting local features and visual transformers (ViT/Swin) in modeling long-range dependencies, as well as utilizing bidirectional ConvLSTM (BConvLSTM) to understand temporal and spatial dependencies, this model has been able to provide performance beyond previous models. The key feature of TBConvL-Net is the design of intelligent mid-level connections (skip connections), in which ST and BConvLSTM blocks are combined to exchange information between the encoder and decoder paths in a multi-scale and semantic manner. Depthwise Separable Conv layers are also used to reduce the computational load and a combined loss function (Dice + Jaccard + Boundary) is used to improve the boundary accuracy and stability of training. In terms of experimental results, the model was tested on ten public datasets from seven types of medical imaging modalities (including MRI, X-ray, Fundus, Ultrasound, and Microscopy) and outperformed the leading methods such as U-Net, UNet++, Swin-UNet, ARU-GD, BCDU-Net, and nnU-Net in all of them. For example, in the ISIC 2018 data, the Jaccard index increased from 84.5% in the best existing model to 91.6% in TBConvL-Net; in the BUSI data, an improvement of more than 24% in the Jaccard Index was also reported. In addition, the model with 9.6 million parameters, 15.5 GFLOPs and an estimation time of 19.1 ms is up to 2.8 times lighter and faster than Swin-UNet (27.3 M parameters and 34.8 ms estimation time), while also having higher segmentation accuracy. Overall, TBConvL-Net, relying on the combined CNN-Transformer-BConvLSTM architecture, has been able to create an unprecedented balance between reducing computational complexity and increasing segmentation accuracy. However, challenges such as the need for a long training time and the dependence on accurate labeling of medical data are still its main limitations.

The asymmetric adaptive heterogeneous network (AAHN) model is introduced in Zheng et al. (2025) as an advanced approach for multimodal medical image segmentation. Unlike traditional symmetric networks, the AAHN is designed with an asymmetric, two-path structure to process the leading modality as the dominant source and the other modalities as auxiliary. Its architecture consists of two complementary branches: a CNN branch for extracting local features and a Transformer branch for modeling global dependencies, which are adaptively combined through the Transformer–CNN feature alignment and fusion (T-CFAF) module. At higher levels, the cross-modality heterogeneous graph fusion (CMHGF) module uses GNN to model heterogeneous relationships between modalities in graph space, leading to more accurate information fusion. In the evaluation on six important datasets including BraTS2023, Prostate158, Hecktor21, and CHAOS, the AAHN model outperformed all competing methods (including nnU-Net, TransUNet, Swin-UNet, and MissFormer) on average. In BraTS2023, it achieved Dice = 0.9371 and HD95 = 2.78 mm, which is a 9% increase in accuracy and a 4.6 mm reduction in marginal error compared to the best previous models. Also, in CHAOS, Dice accuracy reached 0.9206 and 0.9350 for liver and kidney, respectively, and 0.7833 and 0.8921 for PZ and TZ regions in Prostate158. Ablation analysis showed that the T-CFAF module improved the average Dice by 2% and the CMHGF module by 2.1%. Overall, AAHN, by utilizing an asymmetric structure and intelligent combination of CNN, Transformer, and GNN, is an effective step toward increasing the accuracy, stability, and generalizability of multimodal segmentation and provides a new paradigm for integrating heterogeneous data in medical vision.

The HiDiff, i.e., hybrid diffusion framework model, introduced in Chen T. et al. (2024), offers a hybrid approach to medical image segmentation that combines the capabilities of discriminative models (such as U-Net and TransUNet) with the reconstruction and generalization power of diffusion-based generative models (Diffusion Models) in a single framework. The model consists of two main components: the discriminative segmentor, which generates the initial labeling map, and the Diffusion Refiner, which is designed based on the Bernoulli binary diffusion model (BBDM) and refines the output in successive stages. The key innovation of HiDiff is the use of the Bernoulli diffusion process to model binary data instead of the conventional Gaussian noise and the use of the cross Transformer (X-Former) module for the two-way interaction between the discriminative and generative features. This combination results in a model that has improved both spatial accuracy and stability. The reported results indicate that in evaluations conducted on four reference datasets—including Synapse for abdominal organs, BraTS-2021 for brain tumors, Kvasir-SEG, and CVC-ClinicDB for endoscopic polyps, and DRIVE and CHASE_DB1 for retinal images—HiDiff consistently outperformed state-of-the-art models such as nnU-Net, Swin-UNet, MedSegDiff-V2, and PD-DDPM. For example, on the Synapse dataset, it achieved significant improvements in Dice and HD95 metrics over MERIT and BerDiff, and on BraTS-2021, the model was able to reconstruct tumor boundaries with higher accuracy than other methods. Furthermore, cross-set tests demonstrated that HiDiff has higher generalizability and sensitivity in detecting fine structures. In terms of performance, the binary version of the model performs about 10 to 22 times faster than heavier diffusion models while maintaining the same level of accuracy, and has significantly lower memory consumption. HiDiff, by integrating discriminative and generative learning, using a binary diffusion filter, and designing an alternating collaboration mechanism, is a step toward developing accurate, fast, and interactive models for multimodal medical image segmentation.

#### 5.2.4 Transformer alternatives

Transformer alternatives employ novel architectures to model long-range dependencies with lower computational complexity than standard self-attention. Vision state space models (SSMs) have recently emerged as efficient alternatives to transformers by modeling long-range dependencies with linear computational complexity.

The Lightweight Mamba U-Net (LightM-UNet), introduced in Liao et al. (2024), provides an approach for medical image segmentation in resource-constrained environments. Aiming to replace the heavy CNN and Transformer structures in the U-Net architecture, this model leveraged the Mamba-type SSM to model long-range spatial dependencies with linear complexity and very low computational consumption. The proposed architecture includes the innovative residual vision Mamba (RVM) Layer that, by utilizing residual connections and adaptive regularization coefficients, enhances the model's ability to extract deep features and model global dependencies, without adding significant new parameters. In evaluations conducted on two standard datasets, LiTS (for liver and tumor CT) and Montgomery & Shenzhen (for lung X-ray), LightM-UNet outperforms leading models such as nnU-Net, SwinUNETR, UNETR, and U-Mamba with only 1 to 1.87 million parameters and 267 to 457 gigaflops. Specifically, on the 3D LiTS dataset, this model achieves an average Dice = 84.58% and mIoU = 77.48%, which is a 2.5% improvement in mIoU over nnU-Net, with a 47x reduction in parameters and a 15x reduction in computational cost. Also, in 2D X-ray data, LightM-UNet with Dice = 96.17% and mIoU = 92.74% showed about 0.5% higher accuracy and 99% reduction in parameters compared to the U-Mamba model. The results of analytical experiments showed that removing residual connections or tuning coefficients causes a drop of about 0.7% in mIoU, confirming the critical role of these components in maintaining the accuracy and stability of the network.

The selective and multi-scale fusion Mamba U-Net (SMM-UNet) architecture, proposed in Li G. et al. (2025), is one of the advanced Mamba SSM-based architectures for medical image segmentation. The model was designed to simultaneously improve the accuracy of local detail resolution and the understanding of global structures, two factors that cannot be achieved in a balanced manner in many CNN and Transformer models. In the proposed architecture, two innovative modules are introduced: selective fusion Mamba (SF-Mamba) and multi-scale fusion Mamba (MF-Mamba). The SF-Mamba module allows for dynamic selection between spatial and textural information by adaptively assigning weights to local and global features, while MF-Mamba enables the model to detect changes in lesion size and shape with higher accuracy by learning long-range dependencies between feature maps at different scales. SMM-UNet has a similar U-shaped structure to U-Net, but by replacing the classic convolution blocks with Mamba-based layers, it reduces the computational complexity to a linear level and simultaneously integrates multi-scale features effectively. In experiments conducted on the public datasets ISIC2017, ISIC2018 (skin lesion images) and BUSI (breast ultrasound images), this model with only 0.038 million parameters has been able to outperform heavy models such as UNet (4.096M), VM-UNet (27.42M), VM-UNet V2 (22.77M), and even the lightweight version UltraLight VM-UNet (0.038M). Specifically, SMM-UNet achieved IoU = 84.69% and DSC = 91.57% on the ISIC2017 dataset, which is a 2%–3% improvement over the baseline models, and on the BUSI dataset with high noise, it achieved IoU = 68.80% and DSC = 81.12%, which is a 2%–8% improvement over the competing models. The results of the ablation study showed that both SF-Mamba and MF-Mamba components significantly improved the performance, while the increase in the number of parameters was almost negligible. The use of Dice Loss and BCE Loss in the training process also helped to improve the accuracy of the segmentation boundaries.

In line with the utilization of vision foundation models in medical image segmentation, the pioneering research Swin-UMamba† (Liu J. et al., 2025) took an innovative step in combining the computational efficiency of the Mamba structure with the generalizability of transformer-based models. By maintaining the linear complexity of Mamba and the modular design based on Swin, the model managed to significantly reduce the number of parameters and computational resources, while providing performance beyond seven state-of-the-art methods. According to the authors, Swin-UMamba† showed more than 3.9% improvement in Dice index and 4.0% increase in normalized surface distance (NSD) compared to the U-Mamba model, and achieved up to 13% higher accuracy than the comparative methods on the AbdomenMRI, Endoscopy, and Microscopy datasets. In terms of efficiency, this model requires only 100 training epochs, while other methods require more than 1,000 training epochs to converge. Another key innovation of the research is the introduction of a self-supervised model adaptation scheme to reduce the domain gap between natural and medical data, which improves stability and reduces overfitting on smaller datasets. Overall, the results show that Swin-UMamba†, while maintaining the computational efficiency of Mamba, is able to benefit from the pre-trained knowledge of the base models, providing an efficient model for the development of the next generation of low-cost and accurate models in medical vision.

In line with the development of state space models (SSMs) in medical vision, the vision Mamba UNet (VM-UNet) research (Ruan et al., 2025) has introduced the first fully SSM-based architecture for medical image segmentation. Inspired by the VMamba structure, this model is designed in a U-shaped architecture and fully utilizes vision state space (VSS) blocks in both the encoder and decoder sections. Its asymmetric structure (strong encoder - lightweight decoder) in addition to reducing the computational volume by 0.24 GFLOPs, has maintained high accuracy with limited parameters (~27 M). According to the reported results, VM-UNet achieved Dice scores of 89.03%, 89.71%, and 81.08% on the ISIC17, ISIC18, and Synapse datasets, respectively, which is an improvement of about 2% in DSC and 2.34 mm in HD95 compared to state-of-the-art models such as Swin-UNet and TransFuse. In addition, the analytical results show that the use of pre-trained weights from VMamba-S improves mean intersection over union (mIoU) by 2.67% and DSC by 1.65%. Another key feature of VM-UNet is the use of the 2D-selective-scan (SS2D) selective scan module for multi-directional feature extraction, which allows modeling long-range dependencies with linear complexity. By introducing this model, the researchers have established a baseline for evaluating pure SSM models in the field of medical segmentation and paved the way for future research toward efficient, low-cost models with deeper contextual understanding.

The high-order vision Mamba U-Net (H-vmunet) model is introduced in Wu R. et al. (2025) as a SSM-based framework developed from the vision Mamba architecture for medical image segmentation. The aim of this research is to improve the balance between global understanding, local accuracy, and computational efficiency. In this regard, two key components are introduced: High-order 2D-selective-scan (H-SS2D) to model high-order interactions in 2D sequences and Local-SS2D to enhance local details. Combining these two modules in the form of the H-VSS (High-order Visual State Space) block in the U-Net structure enables simultaneous learning of textual and spatial information. In the feature traversal process, the U-Net jump paths are enhanced with the channel attention bridge (CAB) and spatial attention bridge (SAB) modules to perform multi-scale information fusion with greater accuracy. Evaluations on three datasets, ISIC2017, Spleen, and CVC-ClinicDB, have shown that H-vmunet has a significant advantage over state-of-the-art models such as VM-UNet and MHorUNet, such that the average Dice has increased from 0.907 in VM-UNet to 0.917 and from 0.852 to 0.909 in colonoscopy data. In terms of efficiency, the model has an 82% reduction in computational complexity and a 67% reduction in parameters compared to VM-UNet while maintaining high accuracy. In conclusion, H-vmunet, by utilizing high-order interactions in the state space and lightweight attention mechanisms, is an effective step toward designing more accurate, faster, and more generalizable models in the field of medical image segmentation.

#### 5.2.5 Non-transformer comparators

Non-transformer comparative models provide important benchmark architectures and emerging alternatives for assessing the effectiveness of transformer-based segmentation methods.

The study in Isensee et al. (2024) mentioned that the introduction of the nnU-Net framework was a milestone in 3D medical image segmentation, as it demonstrated that by optimally configuring the U-Net architecture, results on par with state-of-the-art models could be achieved without the need for more complex designs. However, a wave of subsequent research focused on the development of new architectures, claiming superior performance over U-Net; claims that in many cases lacked scientific rigor due to poor evaluation, insufficient data, and lack of fair comparison with reliable benchmarks. By providing a comprehensive and systematic evaluation of CNN-based approaches, transformers, and novel structures such as Mamba, this study demonstrated that combining classic U-Net architectures with updated versions such as ResNet and ConvNeXt and implementing them within the nnU-Net framework remains the most effective path to achieving advanced performance. The results of this study, while emphasizing the research community's bias toward “architectural innovation,” emphasize the need to improve standards of evaluation and empirical validation in future research in order to achieve real progress in the field of medical image segmentation.

In response to the limitations of conventional U-Net and Transformer models in modeling nonlinear relationships and poor explainability, the U-KAN, i.e., U-Net-Kolmogorov–Arnold Networks, presented in Li C. et al. (2025) proposed a framework based on Kolmogorov–Arnold Networks (KAN). By combining the classical U-Net structure and tokenized KAN layers, this model has succeeded in providing more accurate, efficient, and interpretable performance in medical image segmentation and generation. In evaluations conducted on three datasets, BUSI, GlaS, and CVC-ClinicDB, U-KAN achieved an average accuracy of 87.22% in F1 and 78.69% in IoU, respectively, while consuming only 6.35M parameters and 14 GFLOPs of computational power—much lower than more complex models such as U-Mamba or U-NeXt. In addition to the resolution part, the extended version of diffusion U-KAN has also been used as a noise predictor in diffusion-based generative models and has outperformed the classic U-Net versions in terms of e Fréchet inception distance (FID) and d inception score (IS) indices. Qualitative and quantitative analyses show that replacing MLP layers with KAN layers has significantly increased the model's ability to identify accurate texture boundaries and reduce over- and under-resolution errors. Overall, U-KAN is an effective step toward combining explainable mathematical theories with medical vision neural networks and opens a new path for designing interpretable and efficient architectures in both image segmentation and generation.

Zhu et al. (2024) introduced the LMIS which consists of a lightweight medical image segmentation model as a lightweight, multi-scale network for medical image segmentation. This model is designed to reduce the need for computational resources in hardware-constrained clinical environments and is based on two key components: (1) lightweight backbone feature extraction (LBFE) for efficient extraction of semantic features from images using a combination of transition convolution and MobileViT modules, and (2) multi-scale feature interaction guidance (MFIG) framework that facilitates multi-scale information exchange using the spatial and channel feature fusion module (SCFM). The LMIS model has been able to strike a good balance between speed, accuracy, and efficiency. In the evaluation on the ISIC2017 and ISIC2018 datasets, the ultra-lightweight version of LMIS-xxs achieved an accuracy of IoU = 0.8233 and Dice = 0.8962 on ISIC2017 and IoU = 0.8323 and Dice = 0.9085 on ISIC2018 with only 0.524 million parameters and 0.197 gigaflops. In addition, the model inference speed reached 71.26 fps on GPU (RTX 3090), making it one of the fastest segmentation networks in the medical domain. In terms of performance, LMIS achieved a 98% reduction in the number of parameters and a more than 97% reduction in computational cost compared to reference models such as U-Net and MSCA-Net, respectively, while offering higher accuracy. In addition to skin data, the model also outperformed state-of-the-art models such as PraNet and MSNet on intestinal lesion (CVC-ClinicDB) and cell nuclei (DSB2018) segmentation tasks, achieving an average Dice of 92.28%. Overall, it is claimed that the paper introduced a multi-scale and efficient framework for learning spatial and semantic features, providing a new path in developing lightweight, fast, and accurate models for diagnostic applications on edge and mobile devices, and can play an effective role in democratizing medical image analysis.

Table 6 indexes a comprehensive benchmark of transformer-based medical image segmentation methods and their alternative approaches, in which all the references provided are considered and the methods are systematically and integratedly compared and analyzed from the perspective of architectural designs, application areas, performance evaluation criteria, and distinctive features. Furthermore, Table 7 highlights a quantitative benchmark of medical image segmentation methods, in which the performance evaluation criteria are compiled exactly according to the values reported in the original sources and presented in a coherent manner for a transparent and reliable comparison. Table 8 shows peer-reviewed comparisons between transformer-based models or related architectures and non-transformer baselines in medical image segmentation tasks.

**Table 6:** Comprehensive benchmark of transformer/transformer-alternative medical image segmentation methods.

References	Application/ task	Dataset(s)	Transformer type/ architecture	Key methodology	Evaluation metrics	Best reported results	Strengths	Limitations
Guo et al. (2024) (UCTNet)	Multi-organ medical image segmentation	Synapse, ACDC, ISIC2018	CNN–Transformer hybrid; Uncertainty-guided ViT	Transformers operate only on uncertain CNN regions using uncertainty maps; masked self-attention minimizes functional overlap	Dice, HD95	Dice: 89.44% (Synapse), 92.91% (ACDC), 91.15% (ISIC2018)	Reduces CNN–Transformer redundancy; better convergence; strong on hard regions; efficient global modeling	Requires reliable uncertainty estimation; added pipeline complexity; not purely end-to-end transformer
Ou et al. (2024) (ResTrans- UNet)	Liver segmentation (CT)	LiTS2017, 3Dircadb, CHAOS, SLIVER07	Dual-path CNN + Swin Transformer	Parallel CNN and Transformer encoders; feature enhancement unit (FEU) transfers global context into CNN features	Dice, VOE, RVD	Dice: 95.35% (LiTS), VOE: 0.0804, RVD: −0.0007	Strong liver boundary handling; effective fusion; excellent volumetric accuracy	Organ-specific design; heavy architecture; transformer mainly in encoder
Liu H. et al. (2024) (BGHNet)	Lung tumor segmentation (3D CT)	HUST-Lung, MSD-Lung	3D hybrid Transformer (VCSwin) + CNN (VPConvNeXt)	Hybrid local–global context aggregation + boundary-guided refinement decoder; volumetric Swin Transformer	Dice, IoU, Sensitivity	Dice (MSD-Lung): 92.4% (reported SOTA); superior cross-domain generalization	Strong boundary modeling; true 3D global context; excellent on indistinct tumors	Computationally heavy; requires 3D annotation; complex training
Zhu et al. (2024) (LMIS)	Skin lesion and polyp segmentation	ISIC2017, ISIC2018, CVC-ClinicDB	Lightweight CNN + MobileViT	Lightweight backbone + transformer blocks; multi-scale feature-guided fusion; real-time design	mIoU, Accuracy, FPS	mIoU: 82.33% (ISIC2017), 88.34% (CVC-ClinicDB); 71 FPS; 0.524M params	Extremely lightweight; edge/real-time ready; strong efficiency–accuracy trade-off	Lower accuracy than heavy transformers; limited global modeling
Anwar et al. (2025) (ResTrans- UNet enhanced)	Liver and tumor segmentation	LiTS, 3D-IRCADb-01	ResNet50 + standalone Transformer blocks	Pretrained CNN encoder + Transformer bottleneck; hybrid skip-connected decoder	Dice, Sensitivity, Specificity	Liver Dice: 98.4%, Tumor Dice: 94.7% (LiTS)	Very high accuracy; transfer learning; robust multi-dataset performance	2D slice-based; high computation; limited volumetric reasoning
Chen H. et al. (2024) (CTFF-Net)	Parkinson's disease-related nuclei segmentation (brain MRI)	Multi-center clinical MRI datasets	CNN–Transformer interleaved encoder	Alternating CNN and Transformer encoder; cascaded channel–spatial fusion; symmetric boundary attention; adaptive Dice loss for small nuclei	Dice, IoU, HD95, ASD	DSC: 85.4%, IoU: 75.0%, HD95: 1.69 mm	Strong on tiny structures; explicit boundary modeling; exploits anatomical symmetry	Task-specific; limited scalability; moderate computational cost
Hua et al. (2024) (MMDA-TCNN)	3D multi-organ and lesion segmentation	ACDC, BraTS2019, Temporal Bone CT	Lightweight Transformer–CNN hybrid	CNN local features dynamically guide Transformer attention; multi-scale/multi-angle interactive fusion; 9.63M params	Dice, IoU	Dice: 91.8% (ACDC); competitive on BraTS	Lightweight for 3D hybrids; strong detail preservation; reduced attention collapse	Still heavier than CNN-only; architecture complexity
Wu R. et al. (2025) (H-VMUNet)	General medical image segmentation	ISIC2017, Spleen, CVC-ClinicDB	Vision Mamba (state-space; transformer-alternative)	High-order selective scan (H-SS2D) + Local-SS2D; U-Net backbone; global receptive field with linear complexity	Dice, IoU	Dice: 91.6% (Spleen); superior across all three datasets	Breaks quadratic attention cost; strong long-range modeling; low redundancy	Emerging architecture; limited clinical validation; less interpretable
Iqbal et al. (2025) (TBConvL-Net)	Multimodal lesion and organ segmentation	10 datasets (skin, thyroid, optic disc, X-ray, cells, etc.)	CNN + Swin Transformer + Bi-ConvLSTM	Transformer blocks + ConvLSTM in skip connections; composite boundary-aware loss; multiscale contextual fusion	Dice, IoU, Accuracy	Consistent SOTA across 10 datasets (Dice often >90%)	Strong multi-scale context; spatial–temporal fusion; robust generalization	Heavy hybrid; complex training; not real-time optimized
Dai et al. (2024) (I^2^U-Net)	Skin lesion, polyp, brain tumor, and multi-organ segmentation	ISIC, Kvasir, BraTS, and Synapse	Dual-path U-Net with non-local attention (Transformer-like)	Dual-path encoder behaves like unfolded RNN; multi-functional interaction; holistic global–local fusion	Dice, IoU, HD	Dice: up to ~92%–94% across tasks	Strong global–local fusion; feature reuse; good generalization	Not pure transformer; higher memory; complex architecture
Chen J. et al. (2024) (TransUNet extended framework)	Multi-organ and tumor segmentation (2D/3D)	Synapse, BTCV, BraTS2021, and pancreas/vessel datasets	ViT encoder + query-based Transformer decoder in U-Net	Systematic integration of Transformer encoder/decoder; coarse-to-fine cross-attention; supports encoder-only/decoder-only/hybrid modes	Dice, HD95	+1.06% Dice over nnU-Net (multi-organ); +4.30% Dice (pancreatic tumor); surpasses BraTS2021 top-1	Comprehensive study of transformer placement; strong for global organs and small tumors; flexible 2D/3D	High computational cost; large parameter count; heavy training
Li G. et al. (2025) (SMM-UNet)	Skin lesion and breast ultrasound segmentation	ISIC2017, ISIC2018, BUSI	Mamba (state-space) replacing transformers	Selective Fusion Mamba + Multi-scale Fusion Mamba; dynamic local–global fusion; linear complexity; ultra-light U-Net	Dice, IoU	SOTA Dice on ISIC and BUSI with only 0.038M params	Extremely lightweight; linear complexity; strong accuracy–efficiency trade-off	New paradigm; limited clinical validation; fewer volumetric experiments
Cai et al. (2025) (CGDMNet)	Cross-modal general medical segmentation	13 datasets; 5 modalities (CT/ MRI/ US/ dermoscopy/ endoscopy)	Dual-domain deformable Transformer (spatial + frequency)	Deformable spatial attention + frequency-domain Transformer + dynamic FFT adapter + multi-task adaptation learner	Dice, IoU, HD	Consistent SOTA across 13 datasets; strong cross-modal generalization	Explicit spatial–frequency fusion; robust cross-dataset performance	Very complex; high training cost; reproducibility challenges
Lv et al. (2025) (SG-UNet)	CT organ and lung segmentation	Synapse, Lung CT datasets	Swin Transformer with self-guided hybrid attention	Saliency-guided token reallocation; hybrid fine-grained global attention; CT-oriented design	Dice, HD	98.13% Dice (lung), 82.91% Dice (Synapse)	Dynamic attention focusing; strong fine-detail capture for CT anatomy	CT-specific; transformer remains heavy; moderate generalization evidence
Zhang et al. (2025b) (FET-UNet)	Breast ultrasound lesion segmentation	BUSI, UDIAT, BLUI	Parallel CNN + Swin Transformer U-Net	Dual-branch encoder (ResNet + Swin); AFAM aggregation; multi-scale decoder	Dice, IoU	Dice: 82.9% (BUSI), 88.9% (UDIAT), and 90.1% (BLUI)	Strong global–local fusion; robust on noisy ultrasound; multi-dataset validation	Task-specific; transformer mostly in encoder; moderate compute
Du et al. (2024) SegVol	Universal + interactive 3D volumetric segmentation	25 CT datasets (200+ categories); AMOS22, SegTHOR, ULS23	3D ViT encoder + CLIP text encoder + prompt encoders + SAM-like decoder	Pretrain on ~96K unlabeled CT + finetune on 6K labeled CT; point/box/text prompts; zoom-out–zoom-in inference	Dice; interactive prompt performance	Example: +19.25% avg Dice vs SAM-MED2D on AMOS22; +4.28% vs SAM-MED3D on SegTHOR; BBox+text Dice 83.02% (avg), text-only 77.17%	True 3D foundation segmentation with semantic + spatial prompts; large-scale pretraining; efficient volumetric inference	CT-focused training; prompt design required; large-scale training compute (A100 reported)
Ruan et al. (2025) VM-UNet	Medical image segmentation (2D organs + skin lesions)	ISIC17, ISIC18, Synapse	Pure SSM/Vision Mamba U-shape	Encoder/decoder built with VSS blocks; patch merge/expand; additive skip connections	ISIC: mIoU/DSC/Acc/ Spe/Sen; Synapse: DSC/HD95	ISIC17: mIoU 80.23, DSC 89.03, Acc 96.29, Spe 97.58, Sen 89.90; ISIC18: mIoU 81.35, DSC 89.71, Acc 94.91, Spe 96.13, Sen 91.12; Synapse: DSC 81.08%, HD95 19.21	Linear-complexity long-range modeling; strong across ISIC + Synapse; simple pure-SSM baseline	Not a transformer (SSM-based); depends on pretrained VMamba; broader multimodal validation needed
Wu J. et al. (2025) Medical SAM adapter/Med-SA	Promptable medical segmentation (2D → 3D adaptation)	Multi-task evaluation; highlighted BTCV abdominal multi-organ	SAM (ViT-H) + parameter-efficient adapters; 2D → 3D SD-Trans + HyP-Adpt	Freeze SAM; update ~2% parameters (~13M); SD-Trans adapts 2D SAM to 3D; prompt-conditioned adaptation	Dice, HD95 (BTCV)	BTCV (BBox 0.75 prompt): Dice 89.8%, HD95 2.18; +2.9% Dice vs Swin-UNETR with far fewer tunable params	Parameter efficient; explicit 3D adaptation; prompt-conditioned usability	Prompt-quality dependence; domain shift may remain; adaptation may underperform task-trained SOTA in some cases
Wang C. et al. (2024) Trans-VNet	3D tooth semantic segmentation	Dental CBCT: MAD + Cui *et al*. dataset	V-Net + Transformer cross-attention module	Patch-based ROI extraction; cross-attention refined by spatial/channel attention to handle sparsity and artifacts	DSC, IoU	MAD: DSC 88.67%, IoU 87.36%; Cui et al.: DSC 96.44%, IoU 95.17%	Strong on clean and artifact-heavy CBCT; ROI extraction reduces sparsity/artifact impact; cross-attention adds global context	Performance drops with heavy metal artifacts; small dataset size; artifact sensitivity noted
Sivanagaraju et al. 2025)	Liver tumor segmentation	LiverHCC (multiphasic MRI)	CRes2Net feature extractor + Transformer (MDA-SGT)	PFWF preprocessing; Res2Net feature extraction; multi-level dual-aspect self-attention transformer	Dice	Dice = 97.28% on LiverHCC	High reported Dice; explicit enhancement → CNN → Transformer pipeline; targets clinical MRI tumor delineation	Single-dataset evidence; sensitive to preprocessing choices; cross-center robustness not demonstrated
Li C. et al. (2025) (U-KAN)	General medical image segmentation (also backbone for diffusion models)	Multiple public benchmarks	U-Net with tokenized Kolmogorov–Arnold Network (KAN) blocks	Tokenized KAN layers near bottleneck to model nonlinear global relations; interpretable operators	Dice, IoU, HD95	Consistently outperforms U-Net and Transformer-U-Net backbones with lower computation cost	Interpretable global modeling; strong accuracy–efficiency trade-off; promising transformer alternative	Not a pure transformer; gains depend on tokenization; specialized KAN implementation required
Zheng et al. (2025) (AAHN)	Multimodal medical image segmentation	6 multimodal datasets (e.g., BraTS, Hecktor, CHAOS)	Asymmetric Transformer–CNN hybrid + heterogeneous graph fusion	Two-stream asymmetric encoder (CNN for leading modality, Transformer for auxiliary); T-CFAF alignment + CMHGF fusion	Dice, IoU, HD95	Hecktor21: Dice 82.42%, HD95 2.08 mm; BraTS2019 WT 92.80%, TC 87.64%; CHAOS liver 92.06%, RK 93.50%, LK 90.77%, spleen 92.08%; BraTS2023 ranked 1st across most metrics (WT 93.71%, TC 90.54%, ET 86.29%)	Explicit modality discrimination; adaptive fusion; strong multimodal generalization	Complex pipeline; higher training cost; primarily multimodal design
Liao et al. (2024) (LightM-UNet)	2D and 3D organ/lesion segmentation (lightweight deployment)	LiTS, Montgomery & Shenzhen, and other 2D/3D sets	Pure Mamba (SSM) U-Net	Residual Vision Mamba layers; linear complexity global modeling; mobile-oriented design	Dice, IoU	Outperforms nnU-Net while reducing params by ~116× and FLOPs by ~21×	Extremely lightweight; strong long-range modeling; edge deployment friendly	Not attention-based; limited evaluation diversity; sensitive to SSM design
Isensee et al. (2024) (nnU-Net Revisited)	Large-scale benchmark of 3D medical segmentation methods	Many diverse 3D datasets	CNN vs. Transformer vs Mamba comparative benchmark	Controlled re-evaluation (Swin-UNETR, nnFormer, U-Mamba, CoTr, etc.) under nnU-Net framework to reduce validation bias	Dice, HD95	Well-configured CNN nnU-Net variants often match or outperform Transformers/Mamba under fair validation	Critical for survey rigor; exposes validation bias; establishes reliable baselines	Not a novel segmentation model; focuses on methodology not architecture novelty
Chen T. et al. (2024) (HiDiff)	Multi-task medical segmentation (organs/tumors/ vessels/polyps)	Synapse, BraTS2021, Kvasir-SEG, CVC-ClinicDB, DRIVE, CHASE_DB1	Hybrid: segmentor + binary Bernoulli diffusion refiner + X-Former	Discriminative prior mask + binary Bernoulli diffusion refinement; cross-transformer bidirectional exchange; collaborative training	Dice, HD95, IoU, Accuracy, Recall	Consistently outperforms CNN/ Transformer/ diffusion baselines across datasets; SOTA Dice on Synapse and BraTS with gains on small structures	Strong boundary refinement; excellent small-object performance; plug-and-play with various backbones	Added training complexity; iterative refinement increases inference time; not a standalone backbone
Liu J. et al. (2025) (Swin-UMamba^†^)	General medical image segmentation	AbdomenMRI (AMOS 2022), EndoVis 2017, NeurIPS 2022 Cell Seg.	Mamba-based foundation model + U-shaped segmentation (VSS blocks)	Adapts pretrained VMamba; introduces SS2D; self-supervised medical adaptation (masked image modeling) then fine-tuning; supports pure Mamba or CNN decoder	DSC, NSD, F1-score, Params, FLOPs	AbdomenMRI: DSC 77.67%, NSD 84.28%; Endoscopy: DSC 69.31%; Microscopy: F1 62.19%; 28M params, 18.9 GFLOPs	First foundation-model adaptation of Mamba for medical segmentation; strong data efficiency; linear-complexity global modeling; consistent gains over ViT/Swin-UNETR/nnFormer/U-Mamba	Not attention-based transformer; requires pretraining + multi-stage training; limited modality diversity; less standardized than CNN/ViT
Du et al. (2024) (SegVol)	Universal & interactive 3D volumetric segmentation	25 CT datasets + 90k unlabeled CT; AMOS22, ULS23, SegTHOR (>200 categories)	3D ViT encoder + SAM-style promptable decoder; image/ spatial/ semantic encoders	3D foundation model; point/box/text prompts; zoom-out–zoom-in inference; self-supervised pretraining (SimMIM) + CLIP prompting	DSC; efficiency; multi-task generalization	AMOS22: avg Dice 85.93% (vs 66.68% SAM-Med2D); SegTHOR: avg Dice 81.55%; wins 19/22 tasks; up to +37.24% Dice	First universal 3D foundation model; semantic + spatial prompting; efficient volumetric inference; strong generalization	Large-scale compute required; primarily CT-focused; complex multi-stage training/data construction

**Table 7:** Quantitative benchmark of medical image segmentation methods (reported metrics as provided).

References	Dataset/task	Dice (DSC) (%)	Recall/Sens. (%)	IoU (%)	Specificity (%)
Liu J. et al. (2025)	AbdomenMRI–multi-organ MRI segmentation	77.67	NR	NR	NR
Du et al. (2024)	AMOS22–3D abdominal multi-organ CT segmentation (interactive)	85.93	NR	NR	NR
Chen T. et al. (2024)	Synapse–abdominal multi-organ CT segmentation	84.94	NR	NR	NR
Zheng et al. (2025)	CHAOS–multi-organ abdominal CT/MRI segmentation	92.06	NR	NR	NR
Li C. et al. (2025)	BUSI–breast ultrasound tumor segmentation	76.40	NR	63.38	NR
Isensee et al. (2024)	AMOS2022–3D abdominal multi-organ CT segmentation	88.64	NR	NR	NR
Liao et al. (2024)	LiTS–liver & tumor CT segmentation (3D)	84.58	NR	77.48	NR
Ruan et al. (2025)	ISIC 2018–skin lesion segmentation	89.71	91.12	81.35	96.13
Sivanagaraju et al. (2025)	LiverHccSeg–liver tumor MRI segmentation	97.28	NR	94.70	99.83
Wang C. et al. (2024)	Cui et al. CBCT–3D tooth semantic segmentation	96.44	NR	95.17	NR
Wu J. et al. (2025)	BTCV–3D abdominal multi-organ CT segmentation (interactive)	89.80	NR	NR	NR
Chen J. et al. (2024)	BTCV–3D abdominal multi-organ CT segmentation	88.39	NR	NR	NR
Zhang et al. (2025b)	BUSI–breast ultrasound lesion segmentation	82.9	NR	74.7	98.6
Lv et al. (2025)	Synapse–abdominal multi-organ CT segmentation	83.91	NR	72.28	NR
Cai et al. (2025)	ISIC 2018–skin lesion segmentation	91.62	NR	85.58 (mIoU)	NR
Li G. et al. (2025)	ISIC 2017–skin lesion segmentation	91.57	NR	84.69	NR
Wu R. et al. (2025)	ISIC2017–skin lesion segmentation	91.72	90.56	NR	98.31
Hua et al. (2024)	ACDC–3D cardiac MRI multi-structure segmentation	87.77	NR	NR	NR
Chen H. et al. (2024)	Clinical T2-FLAIR MRI–PD-related DGM nuclei segmentation	85.4	NR	75.0	NR
Dai et al. (2024)	ISIC 2018–skin lesion segmentation	90.10	NR	83.66	NR
Iqbal et al. (2025)	ISIC 2018–skin lesion segmentation	95.47	95.29	91.65	98.55
Guo et al. (2024)	ISIC 2018–skin lesion segmentation	91.15	91.13	85.03	97.96
Anwar et al. (2025)	LiTS–liver tumor segmentation from CT	94.7	94.2	89.9	NR
Zhu et al. (2024)	ISIC 2017–skin lesion segmentation	89.62	NR	82.33	NR
Liu H. et al. (2024)	MSD-Lung–3D lung tumor CT segmentation	78.02	81.26	NR	NR
Ou et al. (2024)	LiTS 2017–liver segmentation from CT	95.35	96.61	NR	NR

NR, Not Reported in the original study.

**Table 8:** Same-study non-transformer baseline comparisons in medical image segmentation.

Transformer-based or related model	Dataset/task	Reported model performance	Non-transformer baseline(s)	Reported baseline performance	Interpretation for fair comparison
TransUNet (Chen J. et al., 2024)	Multi-organ and tumor segmentation	Average Dice improvement: +1.06% for multi-organ segmentation; +4.30% for pancreatic tumor segmentation	nnU-Net	Reported as lower by 1.06% and 4.30% Dice in the corresponding tasks	The reported comparison is particularly informative because TransUNet is evaluated against nnU-Net, a strong and widely used non-transformer baseline for medical image segmentation. The observed Dice improvements therefore provide task-specific evidence that transformer-enhanced encoder–decoder designs may improve segmentation performance under comparable evaluation conditions.
UCTNet (Guo et al., 2024)	Synapse, ACDC, and ISIC2018 segmentation	Dice = 89.44% on Synapse; 92.91% on ACDC; 91.15% on ISIC2018	nnU-Net, D-Former, H2Former, ConvFormer	Synapse: UCTNet +2.45% over nnU-Net; ACDC: UCTNet +0.51% average Dice	The same-study evaluation across Synapse, ACDC, and ISIC2018 provides a useful basis for comparing UCTNet with CNN-based, transformer-based, and hybrid segmentation baselines. The reported improvements over nnU-Net suggest that the proposed design may offer added value, particularly when both local boundary information and broader anatomical context are required.
Med-SA (Wu J. et al., 2025)	Multimodal medical image segmentation; BTCV example	BTCV: Dice = 89.8%; HD95 = 2.18 mm	MedSAM, nnU-Net, Swin-UNETR, TransUNet, and CoTr	Compared with Swin-UNETR: +2.9% Dice; 9–11 mm HD95 reduction	The comparison indicates that adapting foundation-style segmentation models to medical imaging can improve both overlap-based and boundary-based performance in the evaluated setting. However, the interpretation should remain linked to the specific dataset, prompt strategy, adaptation procedure, and target anatomy, as promptable segmentation performance can vary across clinical contexts.
LightM-UNet (Liao et al., 2024)	LiTS 3D liver/tumor segmentation and 2D X-ray segmentation	LiTS: Dice = 84.58%; mIoU = 77.48%. 2D X-ray: Dice = 96.17%; mIoU = 92.74%	nnU-Net, SwinUNETR, UNETR, and U-Mamba	LiTS: +2.5% mIoU over nnU-Net; 47× fewer parameters and 15× lower computational cost. 2D X-ray: about +0.5% accuracy over U-Mamba with 99% fewer parameters	The results show that efficient Mamba-assisted or state-space-based segmentation architectures can provide competitive or superior performance while substantially reducing computational cost. This comparison is important because it demonstrates that transformer alternatives may be preferable in resource-constrained or volumetric segmentation settings when efficiency and accuracy are considered jointly.
Swin-UMamba (Liu J. et al., 2025)	AbdomenMRI, EndoVis, and microscopy segmentation	Strong multi-dataset performance reported	CNN-based, transformer-based, and Mamba-based comparators	–	The inclusion of CNN-, transformer-, and Mamba-based comparators enables a broader interpretation of segmentation performance across different architectural families. Swin-UMamba is best interpreted as a hybrid transformer-alternative model, and its results should be considered within the specific dataset, dimensionality, and validation protocols used in each benchmark.
nnU-Net Revisited (Isensee et al., 2024)	Large-scale 3D medical segmentation benchmarking	Well-configured CNN nnU-Net variants often match or outperform transformer and Mamba methods	CNN-based nnU-Net, ResNet/ConvNeXt nnU-Net variants, transformers, and Mamba models	–	This benchmark is methodologically important because it shows that carefully configured CNN-based segmentation frameworks remain highly competitive against transformer- and Mamba-based models. The finding emphasizes that architectural comparisons should account for tuning quality, training protocol, dataset scale, and implementation strength, rather than attributing performance differences solely to model family.
I2U-Net (Dai et al., 2024)	Skin lesion, polyp, brain tumor, and abdominal multi-organ segmentation	Superior performance and generalization over state-of-the-art methods	U-Net-derived and other segmentation baselines	–	I2U-Net provides a relevant non-transformer reference for assessing whether transformer-based designs offer consistent advantages over strong U-Net-derived segmentation models. Its inclusion highlights that well-designed convolutional or non-transformer architectures can still achieve strong generalization across heterogeneous segmentation tasks.
U-KAN (Li C. et al., 2025)	Medical image segmentation and generation	Higher accuracy with lower computational cost reported	U-Net-based segmentation backbones	–	The comparison illustrates that non-transformer architectural alternatives, including KAN-based designs, may improve segmentation accuracy and computational efficiency relative to conventional U-Net-style backbones. This supports a more balanced interpretation in which transformer-based models are one important direction, but not the only pathway for advancing medical image segmentation.

Segmentation metrics are highly task-dependent. Dice or IoU values should be compared only when models use the same dataset, target anatomy, dimensionality, annotation protocol, and evaluation split. nnU-Net is retained as a central non-transformer baseline because it remains a strong reference under rigorous validation.

### 5.3 Medical image classification

In medical image classification, Transformer-based models are primarily used to learn discriminative representations from whole images, image patches, regions of interest, or multiple diagnostic regions. Classification studies reviewed in this section cover modalities such as X-ray, CT, MRI, dermoscopy, retinal imaging, and histopathology. Methodologically, these studies include ViT-based models, Swin Transformer variants, CNN–Transformer hybrids, self-supervised and transfer-learning approaches, and multimodal frameworks. The focus of Section 5.3 is not only on reported classification accuracy, but also on how these models address limited annotated data, heterogeneous imaging appearances, local—global feature integration, baseline comparability, and clinical decision-support relevance.

The emergence of ViTs, following their initial success in natural image classification and recognition, has sparked significant interest within the medical image processing community. With the increasing volume of medical image data and advancements in computer vision, computer-aided diagnosis and treatment are becoming integral to modern medicine. ViTs, offering potential advantages over traditional CNNs in image classification tasks, are being explored for their ability to leverage global context and long-range dependencies within medical images. This exploration aims to enhance the accuracy and efficiency of medical image classification, ultimately contributing to improved diagnostic and treatment outcomes.

The reviewed classification approaches are categorized into transformer-based models and hybrid CNN–Transformer models. Transformer-based models rely on vision transformers for global feature extraction, whereas hybrid models combine local convolutional features with global transformer representations.

#### 5.3.1 Transformer-based models

**Figure 5:**
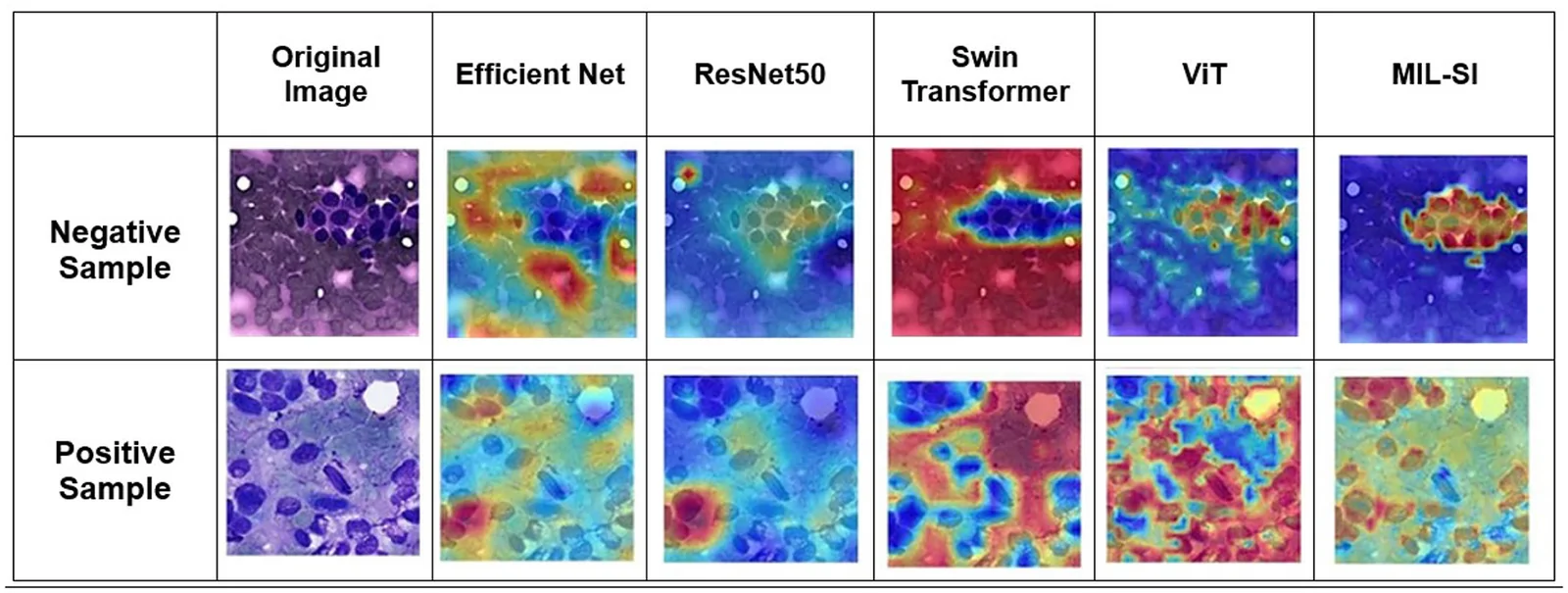
Attention areas of example samples in state-of-the-art methods and SI-ViT. The initial column is the original image of two positive and two negative samples, and the remaining columns are Grad-CAM results. As shown in the image, SI-ViT acquires the most precise attention areas and can precisely identify the outline of the cell regions. Adapted from Zhang et al. (2024).

The study in Zhang et al. (2024) introduced shuffle instance-based vision transformer (SI-ViT), an instance-aware ViTs framework, to address the challenges of automated rapid on-site evaluation (ROSE) for pancreatic cancer diagnosis. Recognizing the complexities arising from staining variations and diverse cancerous patterns, their method employs a novel shuffle instance strategy. By generating shuffled instance 'bags' and corresponding soft labels, SI-ViT learns broader relational distributions, while an un-shuffle step maintains alignment with sample labels. This dual approach enhances the model's ability to capture both intra- and inter-sample relationships, improving feature extraction and robustness. SI-ViT demonstrated significant performance gains in ROSE classification, provided interpretable attention maps, and exhibited strong generalization on other pathological datasets, highlighting its potential for AI-driven on-site cancer diagnosis. Figure 5 illustrates SI-ViT accurately identifies cell contours compared to other methods.

The authors of Yan et al. (2025) proposed discrete wavelet transform (DWT) to neighborhood attention transformer (NAT) (DWNAT-Net), a novel neural network architecture for breast cancer histopathology image classification, aiming to improve early diagnosis accuracy. Recognizing that traditional deep learning methods often neglect frequency domain features, which are crucial for detailed image analysis, their approach integrates DWT with a NAT. DWT is employed to decompose input images into various frequency bands, thereby extracting both frequency and spatial information. NAT, on the other hand, efficiently models local dependencies by confining attention calculations to token neighborhoods. Evaluated on the BreakHis and BACH datasets, DWNAT-Net demonstrated high image-level recognition accuracy, achieving 99.66% and 91.25%, respectively. The results highlight the architecture's competitive performance against existing state-of-the-art techniques, underlining the benefits of incorporating frequency domain analysis into transformer-based histopathology image classification.

More recently, authors in Dahou et al. (2025) suggested a deep learning-based framework and Beluga whale optimization (BWO) for medical image classification. Their method employed the CrossViT model, a vision transformer architecture derived from ViT, to extract meaningful features from medical images. To improve feature selection, they proposed modified BWO (MBWO) that integrates a triangular mutation operator (TMO) to enhance exploitation during optimization. In the Blood Cell dataset, the proposed model achieved the highest classification accuracy of 89.10% and a sensitivity of 84.9%, outperforming other comparative methods. The results demonstrate the potential of combining transformer-based deep learning and metaheuristic optimization for effective medical image analysis. Although the model indicates strong classification capability, the high time complexity of MBWO could limit its scalability to real-word datasets.

The study presented in Nejati Manzari et al. (2026) introduced an enhanced classification approach using Medical Vision Transformer (MedViTV2) which integrates Kolmogorov–Arnold Network (KAN) layers into the Transformer structure. An efficient KAN block was developed to reduce computational cost and improve accuracy. Furthermore, to address the instability of MedViT during scaling and to capture broader contextual information, expand receptive fields, and prevent feature collapse, an enhanced Dilated Neighborhood Attention (DiNA) mechanism was introduced. The MedViT-V2-tiny variant showed excellent results on the PAD-UFES-20 dataset, reaching 63.6% precision, 62.5% sensitivity, 91.7% specificity, 61.2% F1-score, 63.6% overall accuracy, and an AUC of 87.7%, demonstrating its strong accuracy and efficiency for practical medical imaging use. However, the authors noted that the model's robustness varied across corruption types, with color distortions causing a larger performance drop compared to other forms of corruption.

A ST-based method was proposed in Kumar et al. (2025), which achieve an accuracy of 80.76%, precision of 80.28%, recall of 78.04%, and F1-score of 76.46%. The model is an enhanced version of the original ST. Several modifications were made to improve its robustness and efficiency for medical imaging. It can handle low-quality images and process high-dimensional data efficiently using depth-wise convolutions. A lightweight Swin block is also employed to reduce computational cost. The architecture uses a multi-stage hierarchical design that combines self-attention with hierarchical feature fusion. This allows the network to learn coarse-to-fine features progressively. It can also focus on important regions using the self-attention mechanism.

According to the work of Alqahtani et al. (2025), integrating a multi-scale encoding mechanism with Vision Transformer architectures and a dynamic multi-loss function. The multi-scale encoding helps the model capture both detailed and global features. The dynamic loss function adjusts during training to improve classification performance. The method was tested on the ISIC-2018 and ChestX-ray14 datasets. On the ISIC-2018 dataset, it achieved 93.90% precision, 93.70% recall, and a 93.7% F1-score. The results demonstrate that the method improves classification accuracy, precision, and retrieval performance, highlighting its effectiveness for medical image analysis.

As reported in Li Y. et al. (2024), bootstrap own latent of Transformer (BOLT) is a self-supervised learning method for medical image classification. It uses a Transformer backbone with online and target branches to learn useful and consistent features through contrastive learning. BOLT was tested on skin lesion, knee fatigue fracture, and diabetic retinopathy grading tasks. It achieved 85.9% accuracy and an 85.8% F1-score for diabetic retinopathy. However, the model still has some limits. It performs less effectively on segmentation tasks, needs pre-trained weights for other ViT models, and is sensitive to the metrics used for difficulty measurement.

According to the findings of Mohamed et al. (2024), a new breast cancer classification method was proposed using an improved Nutcracker Optimizer combined with Chaos Game Optimization (CGO). They used the Cross Vision Transformer to extract important features from breast images, which helped boost the model's accuracy and reliability. The combined NOCG method achieved 92.86% accuracy and 81.255 sensitivity. The study also suggested that using opposite-based learning and evolutionary techniques could further improve the model's performance.

A recent study by Wang et al. (2026) proposed Paralleformer, a parallel and efficient network for classifying bone lesion images. It looks at both the center and surrounding parts of an image at the same time to capture more details. The Focus Linear Attention module helps the model focus on important information and ignore irrelevant parts. The channel-wise module and residual encoder help the network understand features better. By splitting the original patch embedding into two branches, the model improves the traditional ViT design. Paralleformer achieved 91.05% accuracy, showing better performance for bone lesion classification.

#### 5.3.2 Hybrid CNN-transformer models

**Figure 6:**
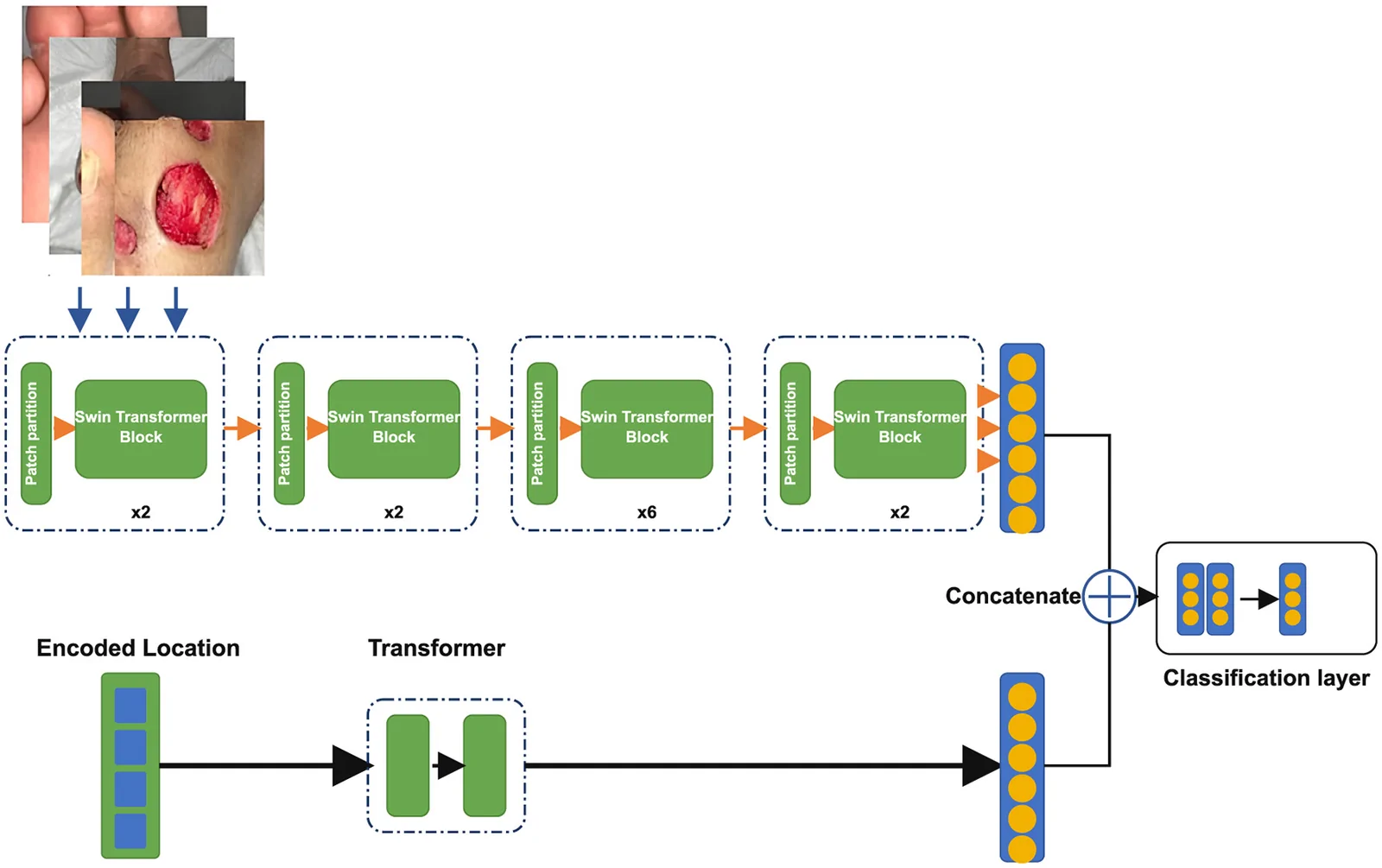
The structural design of the proposed multimodal wound classifier. Adapted from Mousa et al. (2025).

Mousa et al. (2025) introduced a multimodal approach for wound classification, as depicted in Figure 6, addressing the critical need for accurate diagnosis in wound care. Their study combined a ST, for extracting image-based features, with a standard Transformer, used to process location information encoded as a decimal map. This fusion of visual and spatial data aimed to improve the classification of diabetic, pressure, surgical, and venous wound types, which are often indicative of underlying complications. The proposed model, designed to leverage the strengths of transformers in integrating diverse data modalities, was evaluated on the Advancing the Zenith of Healthcare (AZH) dataset and compared against traditional deep neural networks (DNNs). The results demonstrated a significant improvement in classification accuracy, with F1-scores reaching 82.20% for the four-class classification task. This research highlights the potential of multimodal Transformer-based architectures to enhance wound diagnosis by effectively integrating image and location information.

A hybrid ViT-LSTM algorithm was developed in Hossain et al. (2025) to improve automated stroke diagnosis from CT scans, addressing the challenges of accurate interpretation. The model leverages the ViT for feature extraction, capitalizing on its self-attention mechanisms to capture complex visual information from CT scans and learn hierarchical representations of stroke indicators. To optimize ViT for this task, the MLP layer was removed, enhancing computational efficiency, and layer normalization was incorporated to stabilize training and improve the framework's ability to extract detailed features. A “flatten” layer was also added to expand the model's recognition of complex visual patterns. The sequential information extracted by the ViT is then processed by the LSTM network, capturing crucial temporal dependencies. To mitigate class imbalance and enhance clinical relevance, advanced data handling and explainable artificial intelligence (XAI) methods were incorporated. The model achieved high accuracies on both the BrSCTHD-2023 and Kaggle datasets, outperforming traditional CNN and standalone ViT models. This research demonstrates the potential of hybrid deep learning architectures to significantly advance stroke diagnosis by integrating visual and temporal information with enhanced interpretability.

The challenge of accurately distinguishing between benign, particularly level 3 of thyroid imaging reporting and data system (TI-RADS 3), and malignant thyroid nodules in ultrasound images was addressesd in Sun et al. (2023), a task hindered by subtle feature differences leading to diagnostic inconsistencies. To further enhance diagnostic accuracy and reduce unnecessary biopsies, the authors developed TC-ViT, a ViT-based model enhanced with contrastive learning. Furthermore, the researchers modified TC-ViT by incorporating a convolutional embedding path, termed ConvPatch Embedding, which runs parallel to the standard patch embedding. This addition allows the model to extract contextual and local features, which are then combined with class tokens to enhance classification accuracy. Positional embeddings are added to retain spatial information, and Transformer Blocks are utilized to globally model the semantic and positional features, refining the classification process. The algorithm effectively captures both global and local nodule features by using the nodule image as the region of interest (ROI) and employing contrastive learning to minimize the distance between representations of nodules within the same category. According to the reported evaluation, the presented TC-ViT framework achieved a 86.9% accuracy, outperforming other deep learning models in classification performance.

Retinal disease classification presents a significant challenge due to the subtle and minute nature of early-stage pathological regions, often overlooked by conventional deep learning models. Bi et al. (2023) proposed multiple instance learning with vision Transformer (MIL-ViT), a hybrid architecture designed to address the challenge of detecting subtle pathological regions in retinal disease classification. MIL-ViT integrates the global semantic representation of ViTs with the local feature extraction capabilities of MIL. The pipeline processes image patches as instances, exploiting attention-based MIL aggregation with a calibrated attention mechanism to learn instance contributions. A bag consistency loss enforces alignment between ViT and MIL outputs. To enhance performance, the ViT component was pre-trained on a large-scale fundus image dataset, demonstrating improved generalization over ImageNet pretraining. It is asserted that MIL-ViT outperformed state-of-the-art methods across multiple benchmarks, showcasing its effectiveness in fundus image classification.

**Figure 7:**
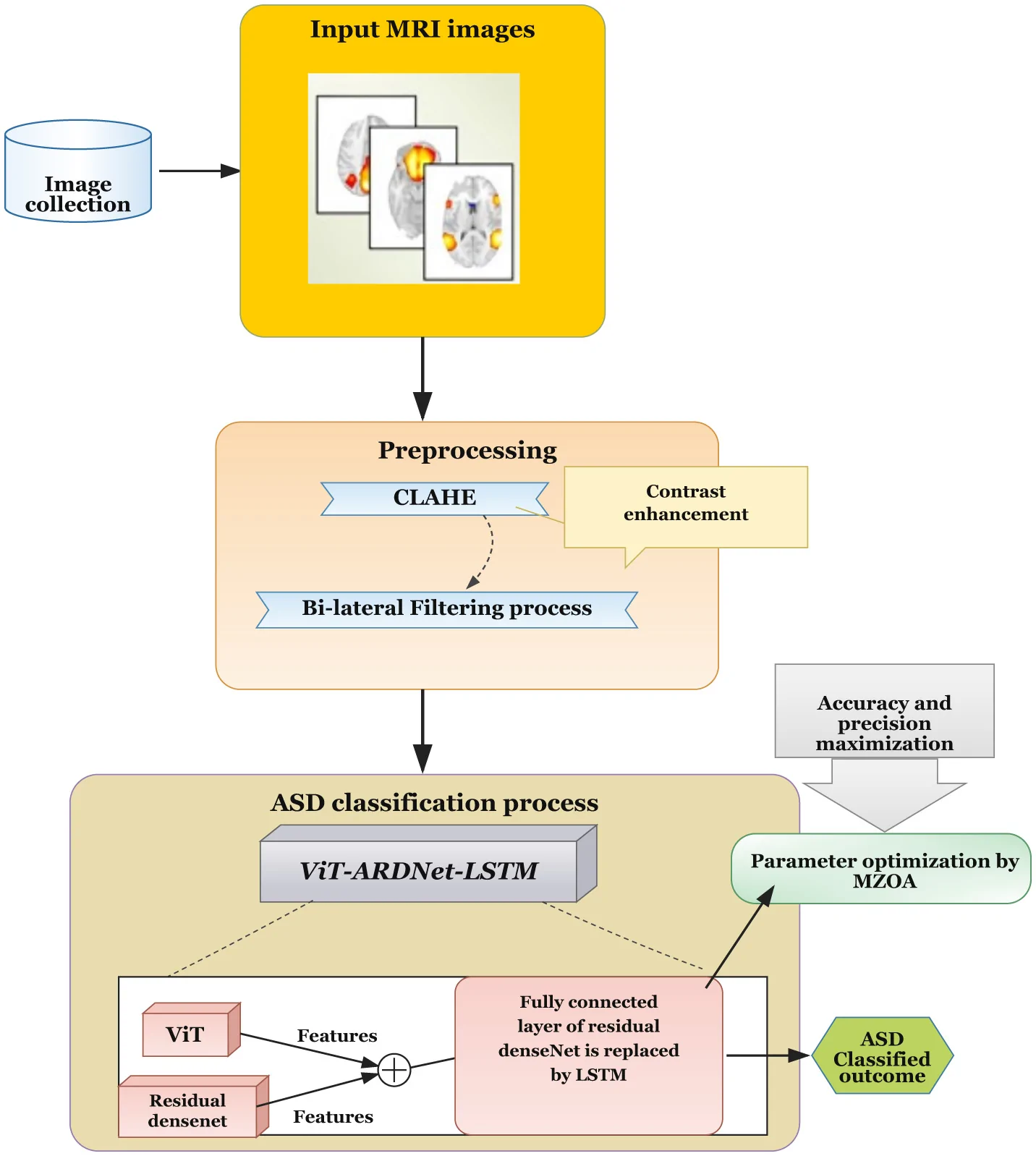
Visual depiction of the ASD classification model, showcasing the architecture of the Vision Transformer-based deep learning approach. Adapted from Parvathy et al. (2025).

The study in Parvathy et al. (2025) has developed a new deep learning model, employing ViTs, adaptive residual densenets, and LSTM (ViT-ARDNet-LSTM), in order to boost the accuracy of autism spectrum disorder (ASD) diagnosis utilizing MRI scans. Recognizing the limitations of traditional diagnostic methods, which often rely on symptom observation and can be unreliable, they sought to leverage the power of deep learning to analyze complex MRI data. The algorithm aims to address the challenges posed by the variability and complexity of MRI images, the need for robust feature extraction, and the importance of optimized model parameters. The ViT-ARDNet-LSTM framework combines the strengths of ViTs, adaptive residual dense networks, and LSTM networks. The process involves preprocessing MRI images using contrast limited adaptive histogram equalization (CLAHE) and bilateral filtering to enhance image quality. These preprocessed images are then fed into the ViT-ARDNet-LSTM model for ASD classification. The model's performance is further optimized using the modified zebra optimization algorithm (MZOA). Experimental results demonstrate that the ViT-ARDNet-LSTM framework achieves a 94% accuracy, precision, specificity. The findings suggest that the proposed model outperforms traditional methods. Figure 7 presents the structure and process of the developed ASD classification system, which utilizes ViTs.

In Li J. et al. (2024), the authors proposed a double branch convolutional transformer (DBCvT), a hybrid CNN-Transformer model that captures detailed features and works well even on small datasets. The model uses separable down sampling convolution (SDConv) to prevent losing important information during down sampling. It also includes a Dual-branch Channel Efficient Self-Attention (DCESA) mechanism to make self-attention more efficient and improve performance. A convolutional channel-enhanced attention module is added to strengthen relationships between feature map channels after self-attention. Tested on the Chest X-ray dataset, DBCvT achieved 97.76% accuracy, 99.38% AUC, 98.97% specificity, and 95.73% sensitivity, showing strong results for medical image analysis.

In the study by Liu S. et al. (2024), the authors proposed Eff-CTM, a hybrid and efficient medical image classification network that combines multi-branch CNN, token grouping Transformer, and mixer MLP modules. The model uses a dual-branch structure that connects CNN, Transformer, and MLP to capture diverse feature representations. In the final stage, an efficient Transformer module is used to extract rich feature information. The model was tested on three public medical image datasets—Chest X-ray, Chaoyang, and Small-ISIC2018—and achieved strong results, including 97.03% accuracy and a 97.63% F1-score on the Chest X-ray dataset. Overall, Eff-CTM achieved better performance with fewer parameters, proving its effectiveness and efficiency for medical image classification tasks. However, the model still needs better performance and a lighter design for clinical use.

A different approach was proposed by Song et al. (2024), who introduced a knowledge distillation method that combines both feature and soft label transfer. In this method, knowledge from a complex teacher model is passed to a simpler student model to improve its accuracy. The teacher model, Res-Transformer, is built on a U-Net design and uses attention layers to capture detailed image features. The student model, ResU-Net, is lighter and uses residual blocks and skip connections to make the network more stable and recover lost image details. Experiments on several disease datasets showed strong results, with the teacher model achieving 96.9% accuracy. However, the approach requires training a large and complex teacher model, which increases computation time and cost. This makes it less practical for lightweight or real-time medical applications.

Researchers in Pan et al. (2024) developed a medical image detection system to identify kidney incidentalomas in CT images using a YOLOv4+ASFF model with a Swin Transformer backbone. The YOLOv4 algorithm detects targets, while the Transformer handles long-range dependencies. The Adaptive Spatial Feature Fusion (ASFF) module helps manage features of different sizes. The model achieved over 87.7% accuracy. However, the study used limited retrospective data and a small sample size. In addition, the data came from only a few centers, which reduces diversity in imaging machines.

In Wu et al. (2023), the authors proposed a hybrid deep learning model called CTransCNN for medical image classification. The model combines CNN and Transformer branches, each including modules to enhance feature attention, capture residual information from multiple paths, and allow interaction between features. It was tested on ChestX-ray11, NIH ChestX-ray14, and a self-collected Chinese medicine tongue dataset (TCMTD), achieving an AUC of 84.56. The main limitation is that combining CNN and Transformer increases the model's computational complexity. It may also need more diverse data for better performance.

In Nejati Manzari et al. (2023), researchers developed MedViT, a robust and efficient hybrid model for medical image classification. The model uses strong building blocks and adds a new data augmentation method that combines feature normalization with regular augmentation. It makes self-attention faster and simpler by using a convolution-based approach. It also learns smoother decision boundaries to be more resistant to adversarial attacks. Tested on 12 medical datasets, MedViT-S (224) achieved 94.2% AUC and 85.1% accuracy. A limitation is that the model may still face challenges in real-world clinical deployment.

Khaliq et al. (2025) proposed a hybrid AI model combining Vision Transformers and Perceiver IO for multi-disease medical image classification. ViT captures global features in images, while Perceiver IO improves feature extraction efficiency and precision. The model performed very well across different diseases: for neurological disorders, it achieved 99.0% accuracy and 99.0% F1-score; for skin diseases, 95.0% accuracy and 95.0% F1-score; and for lung diseases, 98.0% accuracy and 98.0% F1-score. However, the model faces challenges for real clinical use, including high computational requirements, data quality issues, uncertain generalizability, and ethical and deployment concerns.

In Kari et al. (2026), the authors proposed SMMCTE, a hybrid model for tumor detection and classification. It uses dropout and L2 regularization to prevent overfitting, a transformer with fusion blocks (SCMMCT) for accurate tumor segmentation, and Smart Learning to transfer segmentation features for classification. EfficientNetB7++ with a conformer block is used to improve classification accuracy. Tested on BraTS 2020 and BraTS 2021 datasets, the model achieved 99.6% and 99.3% accuracy, respectively. Overall, SMMCTE improves robustness, reduces overfitting, handles class imbalance, and enhances interpretability.

#### 5.3.3 Interpreting quantitative measures reported in heterogeneous studies

The quantitative values reported in this review should be interpreted with caution and within the context of the experimental design of each study. Because the reviewed studies used different datasets, tasks, modalities, training/validation/testing splits, validation strategies, and baselines, the numbers reported in the tables should not be taken as direct comparisons or definitive rankings of the models. For example, the accuracy reported in a binary classification problem on a small, private data set is not directly comparable in difficulty and clinical validity to the accuracy reported in a multiclass, multicenter, or severely class imbalanced problem. Similarly, the DICE score in the 2D segmentation of a small lesion has a different nature with the DICE score in the 3D segmentation of multi-organ or multi-structure and should be interpreted in the context of the same task and evaluation protocol.

Therefore, the quantitative tables of this paper are presented as a descriptive synthesis of the measures reported in the original studies. The purpose of these tables is to show the range of reported performances, the type of metrics used, modalities, datasets, comparative baselines, and overall architectural trends; Not making a homogenous statistical comparison or drawing definitive conclusions about the absolute superiority of one model family over another. In cases where studies have used standard baselines such as ResNet, U-Net, nnU-Net, VoxelMorph, or other non-transformational methods, these baselines have been considered in the interpretation of the results. However, stronger conclusions about the superiority of a model can only be made when the methods have been evaluated on the same dataset, the same partition, the same criteria, the same validation protocol, and comparable implementation conditions.

Table 9 provides a comparative and systematic summary of transformer-based medical image classification methods, in which the different application areas, architectural designs and configurations, methodological innovations, and contributions, performance indicators, as well as the strengths and limitations of each approach are carefully and analytically reviewed and compared.

**Table 9:** Comparative summary of transformer-based medical image classification methods, highlighting application domains, architectural designs, methodological contributions, performance, strengths, and limitations.

References	Application	Dataset(s)	Transformer architecture	Key methodology	Evaluation metrics	Best reported results	Strengths	Limitations
Sun et al. (2023)	Thyroid nodule malignancy classification (TI-RADS 3 vs. malignant)	Private thyroid ultrasound dataset	TC-ViT (ViT + ConvPatch + contrastive learning)	ConvPatch embedding integrates local texture with global context; contrastive learning between full thyroid image and segmented nodule ROI	Accuracy, Sensitivity, Specificity, and AUC	Accuracy 86.9%; improved sensitivity and specificity over CNN baselines	Explicit global–local feature fusion; clinically motivated TI-RADS focus; ROI-based contrastive representation learning	Moderate performance compared to later ViT variants; reliance on private dataset; requires accurate segmentation preprocessing
Yan et al. (2025)	Breast cancer histopathology classification	BreakHis, BACH	DWNAT-Net (Neighborhood Attention Transformer + DWT)	Discrete Wavelet Transform embedded into Neighborhood Attention to jointly model spatial and frequency-domain information	Accuracy, Precision, Recall, F1-score	BreakHis: Accuracy 99.66%, F1 ≈ 99%; BACH: Accuracy 91.25%	Explicit frequency–spatial modeling; excellent texture discrimination; strong benchmark performance	Higher computational cost; pathology-specific focus; lack of external clinical validation
Hossain et al. (2025)	Brain stroke detection and classification from CT	BrSCTHD-2023 (private), Kaggle Stroke	Hybrid ViT–LSTM	ViT extracts spatial representations, while LSTM captures sequential dependencies; includes imbalance handling and explainable AI (attention, SHAP, and LIME)	Accuracy, Precision, Recall, and F1-score	Private CT: Accuracy 94.55%; Kaggle: Accuracy 96.61%	Hybrid spatial–temporal modeling; integrated explainability; cross-dataset validation	Increased architectural complexity; limited comparison with modern ViT variants; scalability analysis missing
Bi et al. (2023)	Retinal disease classification (DR, glaucoma, AMD)	APTOS 2019, RFMiD, LAG, iChallenge-AMD	MIL-ViT (Multiple Instance Learning ViT)	Dual-branch ViT with MIL pooling and calibrated attention; large-scale fundus pretraining	Accuracy, AUC, Sensitivity, Specificity	State-of-the-art AUC and accuracy across all datasets (AUC > 0.90)	Strong global–local semantic modeling; patch-level interpretability; excellent cross-dataset generalization	Complex training pipeline; dependence on large pretraining datasets; increased computational cost
Zhang et al. (2024)	Pancreatic cancer ROSE cytopathology classification	Private ROSE dataset	SI-ViT (Shuffle Instance ViT)	Shuffle/unshuffle instance strategy models intra- and inter-sample relations and suppresses staining variability	Accuracy, Sensitivity, Specificity, AUC	Accuracy > 95%; superior sensitivity and specificity over CNN/ViT baselines	Instance-aware representation learning; robustness to staining noise; interpretable attention maps	Evaluated only on ROSE-style data; careful bag construction required; limited public benchmarks
Li J. et al. (2024)	General medical image classification	Multiple public medical datasets	DBCvT (Double-Branch CNN–Transformer)	Dual-branch hybrid architecture with separable downsampling, channel-efficient MHSA, and weighted feature fusion	Accuracy, Precision, Recall, F1-score	Consistently outperformed CNN and ViT baselines across datasets	Effective local–global fusion; designed for small datasets; efficient attention design	Architectural complexity; absence of large-scale clinical validation; limited robustness analysis
Parvathy et al. (2025)	Autism Spectrum Disorder classification from MRI	Benchmark ASD MRI datasets	ViT-ARDNet-LSTM	CLAHE and bilateral filtering preprocessing; ViT for spatial encoding; ARDenseNet and LSTM for deep and temporal modeling; meta-heuristic hyperparameter optimization	Accuracy, Precision, Sensitivity, Specificity, and FPR	Accuracy 94%; balanced performance across all metrics	Comprehensive hybrid pipeline; explicit parameter optimization; strong balanced accuracy	Heavy and complex pipeline; dependence on heuristic optimization; limited interpretability
Dahou et al. (2025)	Multi-domain medical image classification	Several benchmark datasets	CrossViT + MBWO	CrossViT for feature extraction; Modified Beluga Whale Optimizer for feature selection	Accuracy, Precision, Recall, and F1-score	Higher accuracy and F1 than baseline deep models across datasets	Optimization-enhanced generalization; applicability across heterogeneous domains	Multi-stage non-end-to-end pipeline; optimizer sensitivity; increased computational overhead
Nejati Manzari et al. (2026)	Robust multimodal medical image classification	17 clean + 12 corrupted benchmarks (MedMNIST-C)	MedViTV2 (KAN-integrated Transformer + DiNA)	First integration of Kolmogorov–Arnold Networks into ViTs; dilated neighborhood attention; hierarchical hybrid design	Accuracy, robustness benchmarks, FLOPs	State-of-the-art on 27/29 benchmarks; +4.6% accuracy; 44% FLOP reduction	Strong robustness to corruption; scalable and efficient design; theoretical novelty	Architectural complexity; extensive benchmarking required; not disease-specific
Kumar et al. (2025)	High-dimensional medical image classification	MRI and CT datasets	Improved Swin Transformer	Iterative hierarchical Swin encoder with adaptive attention for noisy and low-contrast data	Accuracy, Precision, Recall, F1-score, CSI	Accuracy 80.76%; F1 76.46%	Noise-aware multi-scale modeling; suitability for high-dimensional inputs	Lower performance than hybrid models; limited dataset diversity; no robustness evaluation
Wu et al. (2023)	Multilabel medical image classification	ChestX-ray11, NIH ChestX-ray14, and TCMTD	CTransCNN (Parallel CNN–Transformer)	Parallel CNN and Transformer branches with label-correlation modeling and imbalance-aware attention	mAP, AUC, Precision, Recall, and F1-score	Consistent improvements in mAP and AUC over CNN and ViT baselines	Explicit modeling of label dependencies; true CNN–Transformer interaction	Heavy architecture; high training cost; limited modality diversity
Li Y. et al. (2024)	Limited-label medical image classification	Skin, knee fracture, and DR datasets	BOLT (Self-supervised ViT)	Token-wise perturbation SSL; difficulty-aware auxiliary task; ViT pretraining without ImageNet	Accuracy, AUC, and F1-score	Outperformed ImageNet and SSL baselines across all tasks	Removes ImageNet dependency; strong for small datasets; novel difficulty-aware SSL	Requires large unlabeled data; additional pre-training stage; not disease-specific
Song et al. (2024)	Lightweight disease image classification	Multiple radiology and pathology datasets	Res-Transformer teacher → ResU-Net student	Knowledge distillation from heavy ViT-U-Net teacher to lightweight student	Accuracy, loss, model complexity	Teacher accuracy 96.9%; student accuracy +7.2% after distillation	Bridges heavy transformers and deployable models; deployment-oriented	Two-stage training; transformer not retained in final model; limited dataset diversity
Mohamed et al. (2024)	Breast cancer classification	Three breast imaging datasets	CrossViT + CGO-NO	CrossViT feature extraction combined with Chaos-Game optimized feature selection	Accuracy, Sensitivity, and Specificity	Higher accuracy and sensitivity than CNN and ViT baselines	Strong multi-scale modeling; high breast-cancer sensitivity	Not end-to-end; optimizer dependence; limited generalization beyond breast cancer
Pan et al. (2024)	Renal incidentaloma detection and classification	Private renal CT dataset (1,485 images)	YOLOv4 + ASFF + Swin Transformer	Swin Transformer backbone integrated into YOLOv4 with adaptive spatial feature fusion	Accuracy, detection precision, inference speed	Improved detection accuracy and generalization over CNN-YOLO baselines	Multi-scale dependency modeling; joint detection and classification	Primarily detection-focused; private dataset; no cross-center validation
Wang et al. (2026)	Bone tumor pathology classification	Private and public histopathology datasets	Paralleformer	Parallel global–local patch extraction with focus linear attention and residual encoding	Accuracy	Accuracy 91.05%; >4% improvement over CNN and ViT	Efficient parallel modeling; reduced attention complexity; pathology-specific design	Limited tumor scope; no multi-center validation; transformer-heavy for WSI
Liu S. et al. (2024)	Multi-domain medical image classification	ChestX-ray, histopatho- logy, and skin disease datasets	Eff-CTM (CNN + Token-grouped Transformer + MLP)	Multi-stage hybrid with reduced token transformer and mixer-MLP for efficiency	Accuracy, parameter count	Higher accuracy with fewer parameters than CNN, ViT, and MLP baselines	Excellent efficiency–accuracy trade-off; deployment-friendly	No robustness analysis; architecture tuning complexity; task-level specificity limited
Nejati Manzari et al. (2023)	Generalized medical image classification	MedMNIST-2D	MedViT	Hierarchical CNN–Transformer with frequency-aware fusion and robustness-oriented training	Accuracy, AUC, robustness benchmarks	Higher average accuracy and AUC than ResNet and ViT; strong robustness	Robustness-focused design; multi-domain generalization; efficient attention	Not disease-specific; requires standardized datasets; limited interpretability
Khaliq et al. (2025)	Multi-disease classification	CT, MRI, dermoscopy, and X-ray datasets	ViT + Perceiver IO	ViT global encoding with Perceiver IO latent attention for scalable multimodal learning	Accuracy, Precision, Recall, F1-score	Accuracy up to 99.0% and F1 up to 99.0% across diseases	True multi-disease framework; strong cross-modality modeling	Broad scope limits disease-specific optimization; heavy architecture
Kari et al. (2026)	Multimodal brain tumor classification	BraTS 2020, BraTS 2021	SCMMCT Transformer + EfficientNetB7++	Missing-modality compensation transformer with segmentation-guided learning	Accuracy, Sensitivity, and Dice	Accuracy > 99%; Dice ≈ 98%	Handles missing modalities; extremely high clinical metrics	Very complex pipeline; segmentation dependency; high computational cost
Alqahtani et al. (2025)	Medical image classification and retrieval	ISIC-2018, ChestX-ray14	Multi-Scale Vision Transformer	Multi-scale patch encoding with dynamic multi-loss optimization	Precision, Recall, F1-score, AUC, and mAP	ISIC-2018 F1 ≈ 93.7%; AUC > 0.92	Joint classification and retrieval; explicit multi-scale design	Heavy training; large data requirement; complex loss design
Mousa et al. (2025)	Multimodal wound classification	AZH wound dataset	Swin Transformer + Transformer	Dual-stream visual and location-token transformers with feature fusion	Accuracy, Precision, Recall, and F1-score	F1 82.2% (four-class); binary tasks up to 100% accuracy	True multimodal modeling; strong clinical relevance	Limited dataset size; moderate multi-class performance; no external validation

In addition, Table 10 presents a quantitative benchmark of transformer-based medical image classification methods, in which performance evaluation indicators are collected and systematically compared, exactly in accordance with the values reported in the original studies. To avoid any misconceptions about the absolute superiority of transformer-based models, in addition to the main quantitative tables, interstudy comparisons with non-transformer baselines are also reported separately. These comparisons are only presented in cases where the transformer and non-transformer methods were investigated in the same study and under comparable evaluation protocols. Furthermore, Table 11 summarizes peer-reviewed comparisons between transformer-based models and non-transformer baselines in medical image classification tasks.

**Table 10:** Quantitative benchmark of transformer-based medical image classification methods (metrics as reported).

References	Dataset/task (as reported)	Acc. (%)	Prec. (%)	Recall/Sens. (%)	Spec. (%)	F1 (%)	AUC (%)
Yan et al. (2025)	BreakHis–binary breast cancer histopathology classification	99.66	99.47	99.77	NR	99.47	NR
Hossain et al. (2025)	BrSCTHD-2023–brain stroke CT classification (multi-class)	94.55	93.00	93.00	NR	93.00	NR
Bi et al. (2023)	LAG–glaucoma fundus image classification	96.60	NR	96.30	94.40	NR	99.60
Zhang et al. (2024)	ROSE–pancreatic cancer cytopathology image classification (binary)	94.00	91.98	90.68	NR	91.32	NR
Sun et al. (2023)	Thyroid ultrasound–TI-RADS 3 vs malignant nodule classification (binary)	86.90	NR	87.10	86.10	92.40	NR
Parvathy et al. (2025)	ABIDE MRI–autism spectrum disorder classification (binary)	94.60	94.00	94.00	94.00	≈94.00	NR
Dahou et al. (2025)	PH2–skin lesion dermoscopic image classification (multi-class)	99.29	NR	100.00	98.81	NR	NR
Nejati Manzari et al. (2026)	Fetal-Planes-DB–fetal ultrasound plane classification (multi-class)	95.30	95.60	95.30	99.00	95.30	91.00
Kumar et al. (2025)	High-dimensional medical image classification (multi-class)	80.76	80.28	78.04	NR	76.46	NR
Li J. et al. (2024)	Peripheral blood cell microscopic image classification (8 classes)	98.95	NR	99.10	99.85	NR	99.98
Li Y. et al. (2024)	APTOS 2019–diabetic retinopathy grading (multi-class)	85.90	NR	NR	NR	85.80	NR
Song et al. (2024)	COVID-19 chest X-ray image classification (multi-class)	96.90	NR	NR	NR	NR	98.90
Mohamed et al. (2024)	INBreast–mammogram classification (normal vs. abnormal)	92.86	NR	68.18	98.08	NR	NR
Pan et al. (2024)	Renal CT–incidentaloma detection/classification (benign vs. malignant vs. healthy)	87.70	NR	NR	NR	NR	85.20
Wu et al. (2023)	TCMTD–multilabel tongue medical image classification	NR	79.51 (OP)	89.31 (OR)	NR	84.12 (OF1)	84.56
Nejati Manzari et al. (2023)	PneumoniaMNIST–chest X-ray pneumonia classification (binary)	96.10	NR	NR	NR	NR	99.50
Khaliq et al. (2025)	Neurological diseases (stroke + Alzheimer's)–MRI/CT image classification	99.00	99.00	100.00	NR	99.00	NR
Kari et al. (2026)	BraTS 2020–brain tumor MRI classification (benign/malignant/no-tumor)	99.60	NR	99.40	99.20	NR	NR
Wang et al. (2026)	LC25000 (lung subset)–histopathology lung cancer classification	97.77	97.77	97.77	NR	97.77	NR
Liu S. et al. (2024)	Chest-Xray–pneumonia classification (normal vs pneumonia)	97.03	97.02	98.24	NR	97.63	96.64
Mousa et al. (2025)	AZH dataset–four-class wound classification (multimodal: image + location)	83.12	82.09	82.20	NR	82.20	NR
Alqahtani et al. (2025)	ISIC-2018–skin lesion multi-class classification	NR	93.9	93.7	NR	93.7	NR

NR, Not Reported in the original study.

The values in tables are derived from the original studies and should not be interpreted as a direct comparison or definitive ranking of models due to differences in data sets, task difficulty levels, training/testing splits, validation strategies, assessment criteria, and baselines used. The tables are provided solely for the purpose of a descriptive synthesis of reported measures and to identify general trends in performance across studies.

**Table 11:** Same-study non-transformer baseline comparisons in medical image classification.

Transformer-based model	Dataset/task	Reported transformer performance	Non-transformer baseline(s)	Interpretation for fair comparison
TC-ViT (Sun et al., 2023)	Thyroid ultrasound classification	Accuracy = 86.9%	Classical deep-learning/CNN-based networks	The same-study comparison indicates a performance advantage of TC-ViT over classical deep-learning and CNN-based approaches in thyroid ultrasound classification. However, because exact baseline values are not summarized here, the comparison is best interpreted as qualitative evidence of improvement rather than a fully quantitative effect estimate.
Swin-Base with HSW-MSA and SwiGLU (Pacal et al., 2024)	ISIC 2019 eight-class skin lesion classification	Accuracy = 89.36%; Recall = 85.13%; Precision = 88.22%; F1-score = 86.65%	Popular CNN models and other vision models	This comparison provides task-specific evidence for the effectiveness of the modified Swin-based architecture in multi-class skin lesion classification. The interpretation remains most reliable within the ISIC 2019 evaluation setting and should not be generalized beyond comparable class structures, preprocessing, and validation protocols.
MIL-ViT (Bi et al., 2023)	Fundus image classification across public benchmarks	Outperformed recent fundus classification models	Deep learning models and deep multiple-instance learning methods	The reported comparison suggests that integrating vision-transformer representations with multiple-instance learning can improve fundus image classification relative to recent deep-learning baselines. This finding is particularly relevant for weakly supervised or region-level fundus analysis, where image-level labels may not fully localize pathological patterns.
MedViT (Nejati Manzari et al., 2023)	Generalized medical image classification on MedMNIST-2D	Higher average accuracy and AUC; improved robustness	ResNet and other CNN baselines	The MedMNIST-based evaluation provides a relatively standardized setting for comparing transformer-based and CNN-based models across diverse medical image classification tasks. The reported improvements support the potential robustness of MedViT, while the interpretation remains dependent on the benchmark design and task-specific dataset characteristics.
DBCvT (Li J. et al., 2024)	Chest X-ray/medical image classification	Accuracy = 97.76%; AUC = 0.9938; Specificity = 98.97%; Sensitivity = 95.73%	CNN-based medical classification baselines	The reported performance indicates strong discriminative capability in the evaluated chest X-ray and medical image classification setting. Its comparison with CNN-based baselines is informative when interpreted within the same dataset, split, and evaluation protocol, without implying architecture-wide superiority across unrelated classification tasks.
Eff-CTM (Liu S. et al., 2024)	Chest X-ray, histopathology, and dermatology classification	Chest X-ray: Accuracy = 97.03%; F1-score = 97.63%	CNN, ViT, and MLP baselines	This comparison is methodologically informative because the model is evaluated against multiple architecture families, including CNN-, ViT-, and MLP-based baselines. The reported results suggest that hybrid architectural design may improve classification performance while maintaining efficiency, particularly when local and global representations are jointly exploited.
YOLOv4+ASFF Swin Transformer (Pan et al., 2024)	Renal incidentaloma detection and classification in CT	Improved detection accuracy and generalization	CNN-YOLO baselines	The comparison reflects the benefit of incorporating transformer-based contextual modeling into a CNN-YOLO detection framework for renal incidentaloma analysis. Since the task combines detection and classification, the results should be interpreted as evidence for hybrid object-detection performance rather than as a direct comparison among pure image-classification models.
Paralleformer (Wang et al., 2026)	Bone tumor histopathology classification	Accuracy = 91.05%; >4% improvement reported	CNN and ViT baselines	The reported relative improvement suggests that the parallel architectural design can enhance histopathology classification compared with CNN and ViT baselines evaluated in the same study. The finding remains specific to the histopathology task and should be interpreted in relation to patch-level sampling, staining variability, and dataset composition.
ViT–Perceiver IO (Khaliq et al., 2025)	Multi-disease classification using MRI, X-ray, and dermoscopy	Accuracy up to 0.99; F1-score up to 0.99	Conventional architectures	The multi-disease evaluation indicates that transformer-based and Perceiver-style modeling may provide flexible representation learning across heterogeneous imaging modalities. However, because the tasks span different diseases and modalities, the comparison is most appropriately interpreted as broad evidence of adaptability rather than a direct equivalence across all classification settings.
Swin Transformer + Transformer fusion (Mousa et al., 2025)	Multimodal wound classification	Four-class F1-score = 0.822; binary-task accuracy up to 1.0	DNN-based classifiers	The same-study comparison supports the value of multimodal transformer-based feature fusion for wound classification. The binary and four-class results should be interpreted separately, as class granularity, label imbalance, and task difficulty can substantially affect the apparent magnitude of performance improvement.

These comparisons should be interpreted only when transformer and non-transformer methods were evaluated within the same study and under comparable data splits, preprocessing, and metrics.

### 5.4 Medical image reconstruction

In medical image reconstruction and quality improvement, the main focus is on recovering higher quality images from incomplete, undersampled, noisy, or artifactual data. This is of particular importance in applications such as MRI, CT, CBCT, PET reconstruction, multimodal, and super-resolution imaging, because reducing imaging time, reducing radiation dose or improving image resolution should not result in loss of diagnostic information. Transformer-based models in this field have been commonly used to model large-scale structural dependencies, integrate spatial and frequency information, recover anatomical details and reduce artifacts. This subsection analyzes reconstruction studies based on the type of imaging problem, type of input data, dominant architecture, image quality criteria, and computational constraints.

In the field of medical image reconstruction and quality improvement, the reviewed studies mainly focus on MRI, CT, CBCT, PET, and multimodal image reconstruction. Unlike segmentation and classification, in this field the role of transformers appears more in modeling long-range dependencies, integrating spatio-frequency information, reconstructing structural details, and reducing artifacts. Therefore, the studies in Section 5.4 are analyzed based on the type of input data, reconstruction goal, type of transformer or hybrid architecture, and image quality metrics such as PSNR, SSIM, and reconstruction error.

The reconstruction of 3D anatomical structures from medical images is an important area, and Transformer networks have emerged as a significant tool in this domain. Their ability to model global relationships and long-range dependencies makes them well-suited for capturing the complex spatial information required for accurate 3D reconstruction from 2D projections or other forms of sparse or incomplete data. By learning these intricate relationships, Transformers can contribute to generating more coherent and detailed 3D volumes, potentially overcoming some of the limitations of traditional reconstruction methods.

The reviewed reconstruction approaches are organized into three architectural categories: transformer-based reconstruction models, hybrid CNN–Transformer models, and transformer alternatives.

#### 5.4.1 Transformer-based models

**Figure 8:**
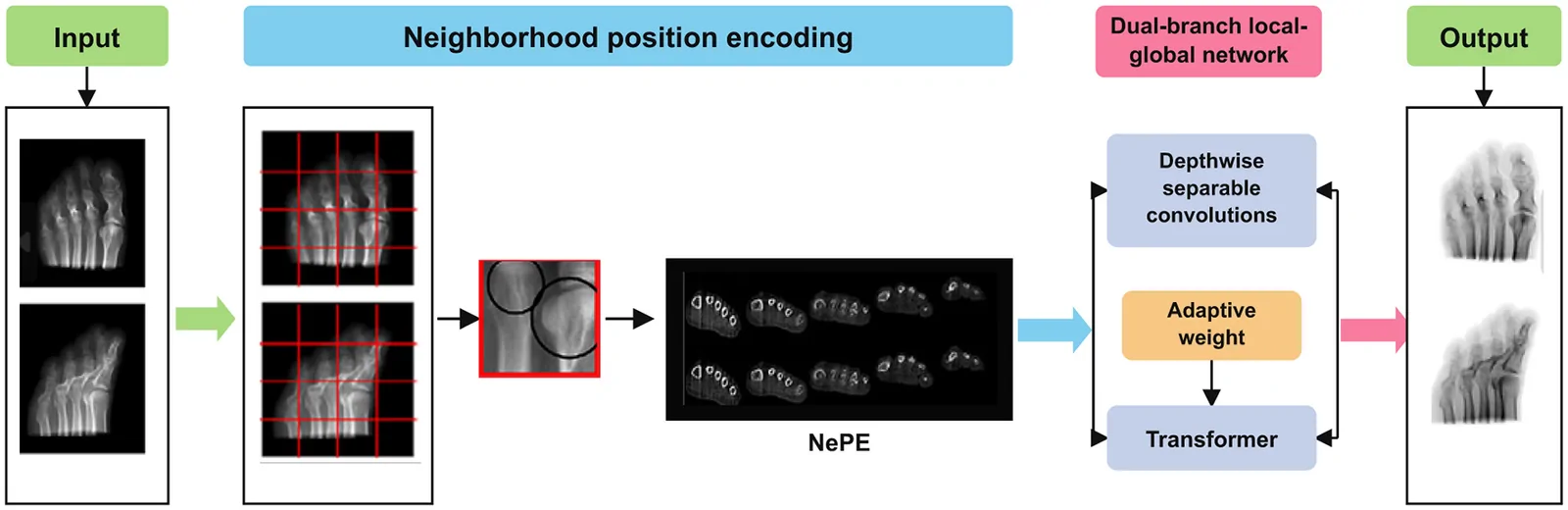
A sparse-view X-ray 3D foot reconstruction method that utilizes a neighborhood Transformer framework. Adapted from Wang W. et al. (2025).

As shown in Figure 8, the authors in Wang W. et al. (2025) proposed a technique based on a neighborhood Transformer to enhance reconstruction accuracy and detail preservation. Wang et al. address the challenges of sparse-view X-ray 3D foot reconstruction, which is crucial for analyzing foot bone structures but limited by existing methods in handling sparse data and complex anatomy. A new neighborhood position encoding strategy is introduced, dividing X-ray images into relevant local regions selected via nearest neighbors to capture detailed features. Building on neural radiance fields (NeRF), they introduce a neighborhood Transformer module, which improves the representation of complex foot bone structures using depthwise separable convolutions and a dual-branch localglobal Transformer network. An adaptive weight learning strategy within the Transformer further enhances the model's ability to capture long-distance dependencies, improving its handling of sparse views.

Li Y. et al. (2025) address the challenge of over-smoothing and detail loss in deep learning-based sparse-view CT reconstruction, which occurs due to the lack of high-frequency information when projection views are severely under sampled. They propose TD-STrans, a Tri-Domain sparse-view CT reconstruction model based on sparse Transformer, which integrates frequency domain information into the traditional projection-image domain approach. TD-STrans comprises three key modules: a projection domain recovery module (PDRM) to complete sparse projections, a Fourier domain filling module (FDFM) to mitigate artifacts and over-smoothing by filling missing high-frequency details, and an image refinement module (IDRM) to further enhance image details. A multidomain joint loss function is designed to simultaneously improve reconstruction quality in the projection, image, and frequency domains, enhancing detail preservation. Simulation experiments on a lymph node dataset and real experiments on a walnut dataset demonstrate TD-STrans' effectiveness in removing artifacts, suppressing oversmoothing, and preserving structural fidelity. The integration of a sparse transformer across these three domains offers a novel solution for ultra-sparse-view CT imaging by alleviating over-smoothing and detail loss caused by reduced views (Li Y. et al., 2025).

The study in Cao et al. (2025) developed mask-based Swin Transformer network (MBST), a mask-based ST network, to enhance 4D cone-beam computed tomography (4D-CBCT) reconstruction quality, particularly under limited scanning conditions and high scanning speeds. Trained on 4D-CBCT data from 20 thoracic tumor patients with simulated sparse views, MBST leverages the Swin Transformer's multi-head self-attention for long-range feature modeling and sliding windows for efficiency. A key aspect is a masking strategy that randomly obscures input image pixels during training, forcing the network to learn textural and structural information for recovery, thus improving generalization to different scanning conditions. Quantitative evaluations on simulated data showed significant improvements in SSIM and PSNR compared to original 4D-CBCT and other deep learning methods across various scanning intervals. Clinical data evaluation on unseen patients also demonstrated substantial improvements in image quality and CT values after MBST optimization, accurately recovering high-density and lung structures. The study concludes that MBST is a highly generalizable reconstruction network for enhancing 4DCBCT image quality across diverse scanning scenarios.

The study in Zhang et al. (2025a) introduced CS-SwinGAN to overcome the limits of current deep learning methods for MRI reconstruction. This model uses a Swin Transformer-based generative adversarial network and includes a Compressed Sensing (CS) Block for pre-enhancement. The architecture has three main stages. First, the CS Block handles frequency-domain loss and gives a pre-enhanced output. The next two stages make up the generator, which works in the image domain. Their results are added together to create the final reconstructed image. Unlike traditional CS-MRI methods that use iterative optimization or only data-driven networks, CS-SwinGAN combines frequency- and image-domain processing in one framework, similar to how MRI reconstruction works in practice. Tests on the fastMRI brain T2 dataset with different sampling patterns showed that CS-SwinGAN always gave better results. For Cartesian sampling, 10% of the usual data, it reached PSNR = 27.68, SSIM = 0.8042, and NMSE = 0.0257. For 20% sampling, it achieved PSNR = 31.32, SSIM = 0.8714, and NMSE = 0.0114. The model performed best with Poisson sampling, likely because of its higher incoherence, and it also reduced noise well in k-space. However, CS-SwinGAN has some drawbacks. It needs a lot of computing power due to its many parameters, and it does not use a complex-valued network design, which limits how well it can capture the full complexity of MRI data. Still, this approach shows promise for combining Transformer models with compressed sensing to improve MRI reconstruction.

The study in Yang T. et al. (2025) addresses the high computational cost of 3D MRI reconstruction by introducing a more efficient method, Slice-to-Volume and Fusion Reconstruction (SVFR). This method uses about 40% less memory than the 3D U-Net but still maintains high reconstruction accuracy. Instead of working with full 3D volumes, SVFR rebuilds 3D MR images from 2D under sampled slices taken from three directions. It uses a pre-trained 2D Vision Transformer, which is adapted for MRI reconstruction by replacing its classification head with a reconstruction head and adjusting its input for three-channel slice data. To recover spatial details lost between slices, a volume-wise fusion model extracts and combines deep features from the three reconstructed volumes. An Attention-Based Feature Fusion (ABFF) module further improves the fusion process and spatial consistency. Tests on large 3D multi-coil brain k-space and Stanford Fully sampled 3D FSE Knees datasets showed strong reconstruction quality and computational efficiency at different acceleration rates. On the 3D Brain dataset at 4× acceleration, the attention-based fusion model achieved NMSE = 0.0195, PSNR = 34.732, SSIM = 0.9351, DSSIM = 0.0325, and LPIPS = 0.0126, outperforming other methods. While SVFR shows strong results, future research will test its performance in real clinical MRI systems to confirm its reliability and practical use in medical imaging.

In Liu J. et al. (2024) researchers developed a new framework called R3ST (Retinex-based Semantic Spectral Separation Transformer) to advance spectral reconstruction for retinal. R3ST can turn standard RGB images into multispectral fundus images, which helps make retinal oximetry easier to access and more affordable. Traditional models often have limited accuracy and do not generalize well across different imaging devices, which can affect diagnostic reliability. R3ST tackles these problems with three main parts: the Self-Supervised Retinex Reflectance Extraction Module (RREM), the Unsupervised Semantic Spectral Separation Module (U3SM), and the Spectral-wise Transformer. The RREM uses Retinex theory to extract reflectance information from RGB fundus images in a self-supervised way, helping the model adapt to different imaging conditions. The U3SM uses unsupervised learning to find semantic information that guides spectral reconstruction, so pixels with different semantic features are treated differently. The Spectral-wise Transformer applies spectral-wise attention to capture long-range and inter-spectral relationships, which improves reconstruction accuracy. They also added an oxygen saturation loss function to further improve performance in clinically important retinal areas. R3ST is tested on two datasets: one from a dual-modal fundus camera and another from a Canon device to check generalization. R3ST outperformed other methods, with a mean MAE-ROI of 3.36, RMSE-ROI of 4.25, and PSNR of 37.22. These results show that R3ST not only improves spectral reconstruction accuracy but also works well across different devices, making it a strong option for practical retinal oximetry and other eye disease diagnostics.

According to Lyu et al. (2023), cardiac cine MRI reconstruction can be improved by using a region-focused multi-view transformer-based GAN architecture. This approach captures complex spatio-temporal patterns, leading to more accurate and detailed images of the heart. It uses a generator-discriminator setup, with the generator based on a multi-view transformer that splits six consecutive cardiac frames into different temporal and spatial views. These include a base view for long-term dependencies, two small views for short-term dynamics, and six tiny views for detailed spatial features. Each view is processed by a spatial-temporal transformer with multi-head self-attention and feed-forward networks, helping the model learn relationships between patches of different sizes. A cross-view attention module brings together information from these views, letting larger views use temporal details from smaller ones to improve temporal correlation learning and reconstruction accuracy. The model also uses a cardiac region detection loss to focus on the heart, which helps produce higher-quality images important for clinical use. Tests on a cine MRI dataset from 30 healthy volunteers, each with 6 slices and 15 temporal frames from TRUFI sequences, showed that this method outperformed current reconstruction techniques. It achieved PSNR = 34.58 dB, SSIM = 0.8745, and RMSE = 1.89, even with a 10 acceleration factor. However, the authors pointed out two main limitations: the model was trained only on single-coil data, which limits its immediate clinical use, and it has a large number of parameters.

In Hosseinimanesh et al. (2025), researchers introduced a deep learning system that can automatically create custom dental crown shapes, making dental restoration design much faster and more accurate. The model uses a partial 3D scan of the prepared tooth, nearby teeth, and the opposite jaw to predict the full shape of the crown. The system is based on a transformer model that learns both small and large details from the 3D scan. The first part of the model looks at the 3D scans using special layers to find local shapes, while the second part uses a transformer with a new attention method and nearest neighbor search to understand both close and distant relationships. To start the second part, another network creates questions from the first part's output, helping the model fill in the missing parts of the tooth step by step. After the crown shape is made, another module turns the points into a smooth and accurate 3D surface. This step uses special math methods for fast and smooth results. The training uses three types of loss: one to improve the fit at the edge, one to better match the shape, and one to control the quality of the 3D surface. Tests on a dataset of 388 training, 98 validation, and 71 test cases showed the model reduced shape errors by 12.32% and surface errors by 46.43% compared to the previous method, with the edge loss giving another 5.59% improvement. The results showing CD-L1 = 54.39, CD-L2 = 8.41, and MSE = 0.0015. The whole process, built in PyTorch and trained with the AdamW optimizer, takes less than five seconds per crown, making it very useful for clinics. However, the model still has trouble showing very fine surface details because of limits in the 3D scan and the transformer. Still, this study shows a strong combination of transformer learning and surface building, moving closer to fully automatic, high-precision dental prosthetic design.

To address the limitations of conventional U-Net–based diffusion models, which often fail to capture long-range dependencies and global anatomical context in MRI super-resolution, a new Diffusion Transformer for MRI Super-Resolution (DiTMSR) is proposed in Tu et al. (2025). Unlike typical diffusion models that use U-Net architectures and are limited by their local focus, DiTMSR uses a conditional Diffusion Transformer backbone to support global reasoning and maintain topological consistency during image reconstruction. The framework works with a low-resolution (LR) target image and can also use a high-resolution (HR) reference image with a different contrast. Both images are encoded, combined with Gaussian noise, and then gradually denoised by the conditional DiT. At each step, cross-attention brings in features from the reference image to keep structures aligned and avoid geometric distortion. To turn the denoised data into high-quality MRI images, DiTMSR uses a Hybrid Mamba Decoder (HMD) with three main parts: a Content Preservation Module (CPM) to keep global structure and main features, six Hybrid Mamba Blocks (HMBs) for efficient decoding, and a self-attention layer to improve local texture. The HMBs mix the MambaVision Mixer with a feedforward network, which allows for efficient global feature mixing, better decoding accuracy, and lower memory use. This hybrid design balances the local detail strengths of CNNs with the global modeling of Transformers, solving the inefficiency of using only attention-based decoders. The CPM also helps keep anatomical accuracy by aligning structures between the LR and generated super-resolution features, making sure features are consistent throughout the network. Tests on the FastMRI and BraTS datasets show that DiTMSR performs better than current leading methods, with PSNR = 34.83 dB and SSIM = 0.8873 on FastMRI, and PSNR = 35.25 dB and SSIM = 0.9017 on BraTS. In summary, DiTMSR brings together the scalability of Transformer-based diffusion models and the efficiency of the Mamba decoder, resulting in high-quality, accurate MRI super-resolution with improved reconstruction and computational efficiency.

#### 5.4.2 Hybrid CNN-transformer models

Han and Du (2025) introduce MHWT, a novel multimodal hybrid window attention Transformer, to address the limitations of the ST's fixed window size in capturing long-range dependencies in high-resolution MRI reconstruction. MHWT employs a retractable attention mechanism with a shape-alternating window design to expand attention coverage while maintaining efficiency. It also uses a variable and shifted window attention strategy for more flexible modeling of local and global dependencies. Enhancements to the Transformer encoder improve training stability and reconstruction performance. Experimental results on multiple public datasets demonstrate that MHWT outperforms state-of-the-art methods in both single-modal and multimodal MRI reconstruction scenarios, showcasing superior image quality and adaptability. The MHWT architecture includes a shallow image fusion module (SIFM) for multimodal input, a shallow feature extraction module using convolutions, a deep feature extraction module (DFEM) composed of retractable attention blocks (RABs) for global context, and a feature fusion and reconstruction module that integrates shallow and deep features at multiple levels to produce the final high-quality reconstructed image.

To improve the reconstruction quality of MRI images from under sampled data, the study in Hou et al. (2025) introduced IGT-Net. IGT-Net combines a Gaussian Mixture Model (GMM) with an Adjacent Spatial Adaptive Transformer (ASAT). The network has two main parts: the Compressive Sensing Sampling Initialization Module (CSS-IM) and the Reconstruction Group. CSS-IM simulates the MRI sampling process and creates initial reconstructions for different sampling rates, which are then sent to the reconstruction group. This group includes two transformer-based modules, TrISTA and TrGMM, that work together to produce high-quality images. TrISTA uses a Dynamic Gradient Descent Module (DGDM) for fast and stable convergence, along with a Transformer-based Proximal Mapping Module (TPMM) that helps keep structural details by integrating nearby spatial features. TrGMM, which includes a GMM-based refinement unit and an ASAT block, improves clarity by removing artifacts and noise using maximum likelihood estimation and adaptive feature extraction. Both TrISTA and TrGMM have a plug-and-play ASAT mechanism to capture long-range dependencies and keep information flowing smoothly between iterations. Tests on public datasets like IXI, StrokeQD, and BrainWeb showed that IGT-Net outperformed recent state-of-the-art methods. Using nine TrISTA blocks and two TrGMM blocks gave the best results, with PSNR = 44.05, SSIM = 99.05, NMSE = 2.16, and RLNE = 10.94. IGT-Net provides strong reconstruction quality and converges efficiently, but its performance decreases a bit at very low sampling rates because aggressive artifact removal can cause over-smoothing. Even so, IGT-Net demonstrates that using transformer architectures with probabilistic modeling can greatly improve MRI reconstruction accuracy and reliability.

In M.khair et al. (2026), the authors introduced VRF-Net, a hybrid deep learning model that combines a vision Transformer with a Residual Feature Network to recover high-resolution system matrices in magnetic particle imaging (MPI). The model tackles issues such as down sampling and changes in coil sensitivity. VRF-Net uses transformer-based global attention and residual convolutional layers to reconstruct both large and small image details. It mimics real MPI conditions with a two-step down sampling process and is trained with paired-image super-resolution on the Open MPI dataset and a simulated coil-sensitivity dataset. The architecture includes a self-attention block with eight attention heads, a multi-layer perceptron with a hidden dimension of 128, and ten residual feature blocks with 128 feature channels each. These blocks use 3 × 3 convolutions and skip connections to keep low-level features, while up sampling is done at scales of 2, 4, and 8, then further improved with extra convolutional layers. The framework also has a transformer module for spatial information, several residual blocks, patch embedding, multi-head self-attention, and up sampling layers. When tested on Open MPI data, VRF-Net showed an average improvement of 88.2% in nRMSE, 44.7% in pSNR, and 34.3%. At 2× scaling on the Open MPI dataset, it achieved nRMSE = 0.403, pSNR = 39.08 dB, and SSIM = 0.835. The model also performed well at higher scaling factors and generalized well to other datasets. Although the lack of *in vivo* validation is a limitation due to unmodeled biological differences, VRF-Net shows promise for improving MPI reconstruction quality and could help future clinical applications.

The WUTrans-MoCo is introduced in Yuan et al. (2025) to improve the quality of 4D-CBCT reconstruction while keeping track of breathing movement. The model uses earlier CT scan data to guide the process. This model combines several advanced parts to fix common problems like streaks, blurry movement, and mismatched body structures. First, the NGJO-Net uses a special method to bring in information from earlier scans and reduce streaks in low-dose, limited-angle scans. Next, the WUTrans part corrects for movement in these scans, removing unwanted image problems while keeping breathing motion realistic. The UQSA feature inside WUTrans helps pull out important details and match information at different levels, making it easier to combine data from different sources and line up images correctly. To make the body structures even more accurate, the SEFF part blends the adjusted CBCT and earlier CT images using a simple two-layer network, which helps avoid mismatches. The model uses ResNet blocks to pull out strong features, and an 8-layer DenseNet decoder to reuse information and show details at different sizes. It also uses a multi-level approach and a step-by-step blending method to better fix changes in lung shapes. Evaluated on the 4D-Lung dataset from the Cancer Imaging Archive (TCIA) and the SPARE Challenge dataset, WUTrans-MoCo achieved excellent results with DSC = 83.1%, SSIM = 0.9094, PSNR = 41.35, and ASD = 0.39 mm. Overall, this method shows strong promise for making high-quality, movement-corrected 4D-CBCT images, which can help make radiation treatment more precise and faster during surgery.

Recently, to improve low-dose sparse-view computed tomography (SVCT) reconstruction, a new network called DdeNet was developed in Lin et al. (2024). It uses special tools called Pale-Transformer and Laplacian convolution to improve image quality and reduce unwanted marks. Older methods often create strong streaks in the images, and CNN-based networks have struggle to capture patterns that are far apart. DdeNet solves these problems by working on both the raw scan data and the final images. For the raw data, a Sinogram Restoration Network (SRN) based on UNet with extra learning steps fixes the missing parts by looking at a bigger area and fixing mistakes from filling in gaps. For the images, a two-part network combines an Image Restoration Network (IRN) and an Edge Enhancement Network (EEN) to make the CT images clearer. The IRN uses a Pale-Transformer block with a simpler type of convolution to better understand the whole image, and the EEN uses Laplacian and multi-size convolutions with a special focus mechanism to sharpen edges and reduce noise. At the end, both sets of features are joined together to make the final image. Tests on the Mayo Clinic Low Dose CT dataset show that DdeNet makes better images than older neural network methods, keeping important details and making clearer, cleaner images even when very few scan images are used. DdeNet achieves superior reconstruction quality with PSNR = 36.06, SSIM = 0.8914, and RMSE = 0.0163.

In Chen et al. (2025), authors proposed a new MRI reconstruction method which is called Dual-Domain Fusion and Cross-Attention Network (DDFCA-Net). The model uses two parallel paths to combine k-space and image-domain information. One path process down-sampled k-space data with a convolutional neural network (CNN), while the other works on a low-quality image using a vision transformer. Both paths start with a Shallow Feature Extraction Module (SFEM), then move through a Dual-Domain Deep Feature Extraction and Fusion Module (DDFEFM), and finally a Reconstruction Module (RM) to produce high-quality MRI images. The DDFEFM contains several Dual-Domain Feature Learning Blocks (DDFLBs). Each block has a k-space Feature Extraction Unit (KFEU) with two point-wise convolutional layers and residual connections, an Image-domain Feature Extraction Unit (IFEU) built with a Transformer to capture long-range dependencies, and a Feature Fusion Unit (FFU) for integrating features and updating data consistency. To make the Transformer more efficient, a Multi-head Enhanced Transposed Attention (ETA) mechanism, based on the Multi-head Deep Convolutional Transposed Attention (MDTA) from Restormer, is used. The RM brings together features from both domains using a point-wise convolutional layer and two 3 × 3 convolutional layers to reconstruct the final image. A key feature of DDFCA-Net is its cross-attention strategy: queries come from image-domain features, while keys and values are taken from the combined k-space and image-domain features. This setup allows for better feature interaction and stronger contextual learning. Tests on the CC359-Brain and FastMRI single-coil knee datasets with different sampling strategies showed that DDFCA-Net performs better than current leading methods. For example, with G1D50% sampling, it improved PSNR by up to 4.01. An ablation study on cross-attention (CA) showed a PSNR of 37.26 dB and SSIM of 0.9499, supporting that combining CNNs in k-space with Transformers in the spatial domain improves feature extraction, long-range modeling, and overall MRI reconstruction quality.

In Wang et al. (2023), the authors present a Hybrid Feature Fusion Neural Network with Transformer integration (HybridF) for DCE-MRI super-resolution. This model uses both convolutional neural networks and transformers to capture local details and broader context. The Hybrid Fusion Block (HFB) in this model merges features from both networks. CNN and residual modules extract low-level spatial features at different scales, while a multi-scale transformer finds high-level global patterns to help focus on important lesion areas. The HFB then fuses these features, using adaptive weighting to combine information from different scales. The model has two parallel branches, each with three 3 × 3 convolution layers, to extract multi-scale features from low-resolution DCE-MRI images. These features are divided into patches and fused in stages to make the most of cross-scale relationships, which helps the network reconstruct detailed anatomical structures. The method was tested on a clinical DCE-MRI dataset with 212 patient cases and the public IXI dataset. It achieved PSNR = 27.39 dB, SSIM = 0.893, and NMSE = 0.01. These results show that HybridF outperforms current DCE-MRI super-resolution methods, offering better reconstruction quality and showing promise for clinical use.

In Wang Y. et al. (2024), a new 3D model called MEaTransGAN was introduced to create high-quality SPET images using low-dose PET and T1-weighted MRI scans. The model combines information about body function and structure using three main parts: a generator, an edge detector, and a discriminator. The generator has four main parts: two encoders based on CNNs, a module that combines features from both scans, a Transformer encoder, and a CNN decoder. The encoders pick out details specific to each scan type—low-dose PET for body function and T1-MRI for body structure—using 3 × 3 × 3 convolutions with batch normalization and LeakyReLU activation. The feature integration module blends these details based on how they relate in space, while the Transformer encoder, made up of four layers of multi-head self-attention and feed-forward networks with layer normalization and shortcut connections, finds patterns across the whole image. The CNN decoder brings back image details using special upscaling filters and skip connections. An edge-aware loss, guided by a Sobel-based edge detector, helps keep the structure accurate. The discriminator looks at small parts of the image to tell apart real and generated SPET images and their edge maps, making sure the results look realistic. Tests on a clinical dataset of 16 people (8 healthy, 8 with mild cognitive problems) and a test dataset showed that MEaTransGAN worked better than other leading methods. For the clinical dataset, it reached PSNR = 25.426 dB, SSIM = 0.988, NMSE = 0.0188 for healthy subjects, and PSNR = 26.041 dB, SSIM = 0.988, NMSE = 0.0221 for those with mild cognitive problems. On the test dataset, it got PSNR = 29.448 dB, SSIM = 0.992, NMSE = 0.004, showing its high accuracy and usefulness in real cases.

Researchers addressed the problem of limited-angle artifacts in Dual-Energy Cone-Beam Computed Tomography (DE-CBCT) by developing the Transformer-Integrated Multi-Encoder Network (TIME-Net) (Zhang et al., 2023). TIME-Net creates high-quality DE-CBCT images from degraded inputs collected with two complementary quarter-scans, which helps lower both radiation dose and scan time. The network includes three encoders (image, prior, and transformer), two decoders (for low- and high-energy channels), and a feature fusion module. Each part has a specific role in the reconstruction process. The image encoder, based on CNNs, extracts important features from the degraded images. The prior encoder uses information from previous images to capture structural details, while the transformer encoder brings in global context by modeling long-range relationships. These features are combined and compressed into shared representations by the fusion module. The two decoders then work on the low- and high-energy channels to restore DE-CBCT images with fewer artifacts. Both the image and prior encoders are adapted from the U-Net architecture to extract features at different scales. The network was trained and tested on clinical DECT images from 25 patients to create a simulated DE-CBCT dataset, and also on real rat data for practical testing. The reconstructed images had a resolution of 512 × 512 × 200 voxels, showing the method can restore fine details. Results showed that TIME-Net performed well, with MAE of 16.39 HU, SSIM of 96.94%, and PSNR of 39.66 dB on simulated data, and MAE of 12.12 HU, SSIM of 93.57%, and PSNR of 36.57 dB on real rat data. Both qualitative and quantitative tests confirmed that the model removes artifacts, recovers subtle structures, and maintains accuracy. Clinical tests, including virtual non-contrast imaging and iodine quantification, also supported TIME-Net's usefulness in medical imaging. Although the system is complex, its modular design makes it possible to combine CNN and Transformer components efficiently.

In Pan et al. (2025), researchers introduced a real-time volumetric imaging framework called A-SI to improve tumor motion tracking during image-guided radiation therapy and reduce radiation exposure. This framework reconstructs cone-beam CT (CBCT) images by combining optical surface imaging (OSI) with single-angle X-ray projections. OSI captures detailed surface features but does not provide internal anatomical information, which can make tumor localization less accurate. To address this, A-SI merges OSI with low-frequency X-ray data using a patient-specific generative model named the Physics-Integrated Consistency-Refinement Denoising Diffusion Probabilistic Model (PC-DDPM). This model creates high-quality Optical Surface-Derived CBCT (OSD-CBCT) volumes. PC-DDPM uses prior anatomical and respiratory motion data from four-dimensional CT (4DCT) scans taken during treatment planning. Its design is based on an encoder-decoder structure with convolutional and Swin Transformer blocks to extract both local and global features. Two main refinement strategies, Physics-Integrated (PI) and Cycle-Consistency (Cycle-C), help ensure the CBCT reconstructions are physically and anatomically accurate. PI refinement uses X-ray projection physics to guide the first-view OSD-CBCT, while Cycle-C refinement uses the first view as a reference to improve later reconstructions and keep them consistent over time. The model uses two DDPMs: Projection-DDPM generates full-angle projections, and CBCT-DDPM creates volumetric data. These are connected by a Geometric Transformation Module (GTM) that provides physics-based guidance. Tests on 56 lung cancer patients showed that A-SI improved image quality and tumor localization, reaching a PSNR of 35.61 dB and SSIM of 0.93, and lowering MAE to 25.13 HU. The model also localized tumors with sub-millimeter accuracy, with an average center of mass distance of 0.60 mm for the first view and 0.67 mm for later views, performing better than standard CBCT methods. However, the framework is still computationally intensive, taking 2.5 seconds for each 3D reconstruction on an A100 GPU.

#### 5.4.3 Transformer alternatives

The work in Huang J. et al. (2025) introduced MambaMIR, an Arbitrary-Masked Mamba-based model with wavelet decomposition for medical image reconstruction and uncertainty estimation, addressing limitations of CNNs (local sensitivity, linear complexity) and ViTs (global sensitivity, quadratic complexity). Mamba, combining linear scalability and global sensitivity, is leveraged in this Ushaped architecture. A novel arbitrary scan masking (ASM) mechanism introduces randomness for uncertainty estimation, providing uncertainty maps without hyperparameter tuning and mitigating the performance drop associated with MC dropout in low-level tasks. Wavelet transformation is integrated for enhanced texture preservation and perceptual quality, with a GAN-based variant, MambaMIR-GAN, also explored. Comprehensive experiments across multiple medical image reconstruction tasks demonstrate that MambaMIR achieves the best reconstruction fidelity, while MambaMIR-GAN offers the best perceptual quality, outperforming baselines and stateof- the-art methods. The MC-ASM provides valuable uncertainty maps for clinicians without compromising performance. The architecture includes patch embedding/unembedding, paired encoder/decoder residual blocks with skip connections, arbitrary-masked state space (AMSS) blocks, wavelet-based down sampling/up sampling, and a bottleneck with wavelet-embedded AMSS (WAMSS) and an attention block. Wavelet decomposition features of the input are also integrated into the encoding pathway.

In Zou et al. (2025), a new model called MMR-Mamba was introduced to make multimodal MRI scans better. Multimodal MRI gives extra helpful information for diagnosis and treatment, but the scans take a long time, which makes them hard to use in hospitals. MMR-Mamba uses a model called Mamba, which can find connections across the whole image without needing a lot of computer resources. The system mixes information from both the image space and the frequency space. It has three main parts. First, the Target-guided Cross Mamba (TCM) part works in the image space to boost the target scan features by adding helpful information from the reference scan. Second, the Selective Frequency Fusion (SFF) part works in the frequency space to mix global frequency information and bring back sharp details in the image. Finally, the Adaptive Spatial-Frequency Fusion (ASFF) part links both spaces by sharing useful information between them, so the model gets the best from both. The system was tested on two standard datasets: BraTS for brain MRI and fastMRI for knee MRI. The results showed that MMR-Mamba made better quality images than the best existing methods. For example, on the BraTS dataset, it reached an average PSNR of 40.49 dB and SSIM of 0.984 for 4× faster scans, doing better than CNN and Transformer-based models. Overall, MMR-Mamba brings together image and frequency information to make MRI scans faster and clearer.

Table 12 presents a comprehensive benchmark of transformer-based medical image reconstruction methods, in which different approaches are systematically analyzed and compared in terms of architectural designs, reconstruction strategies, application areas, performance evaluation criteria, and key features. Moreover, a reported-metric benchmarking of the performance of transformer-based medical image reconstruction methods, focusing on the reported evaluation indicators and comparative analysis of the results obtained in different studies, is presented in Table 13. In addition, peer-reviewed comparisons between transformer-based methods or related architectures and non-transformer baselines in medical image reconstruction are reported in Table 14.

**Table 12:** Comprehensive benchmark of transformer/transformer-alternative medical image segmentation methods.

References	Application/ task	Dataset(s)	Transformer type/ architecture	Key methodology	Evaluation metrics	Best reported results	Strengths	Limitations
Hou et al. (2025) (IGT-Net)	MRI reconstruction from undersampled k-space	IXI, StrokeQD, BrainWeb	ASAT Transformer + Deep Unfolding + GMM	Multi-stage unrolled network integrating TrISTA and TrGMM with adjacent-stage feature sharing	PSNR, SSIM, NMSE, and RLNE	Avg. PSNR 38.56 dB, SSIM 92.76%; lowest NMSE and RLNE across sampling rates	Physics-guided interpretability; excellent artifact suppression; robust across sampling ratios	Multi-stage inference cost; mainly 2D MRI; no external clinical validation
Wang W. et al. (2025)	Sparse-view X-ray 3D foot reconstruction	Private sparse-view foot X-ray	Neighborhood Transformer + NeRF	Neighborhood position encoding with dual-branch local–global transformer embedded in NeRF	PSNR, SSIM, and 3D error	Best overall reconstruction accuracy in study	Extremely sparse-view capable; preserves fine bone structures	No public benchmark; high NeRF computation cost
Li Y. et al. (2025) (TD-STrans)	Sparse-view CT reconstruction	CT lymph node, walnut	Tri-domain Sparse Transformer	Joint projection–image–frequency domain reconstruction with Fourier filling	PSNR, SSIM, VIF	60 views: PSNR 42.99 dB, SSIM 98.9%, VIF 0.72	Excellent detail preservation; ultra-sparse robustness	Complex pipeline; accurate domain transforms required
Huang J. et al. (2025) (Mamba- MIR)	MRI/CT/PET reconstruction + uncertainty	fastMRI, SKM-TEA, SVCT, and PET	Mamba + Wavelet + ASM	Linear-complexity global modeling with uncertainty-aware masking	PSNR, SSIM, LPIPS	fastMRI ×4: PSNR 41.59 dB, SSIM 98.0%	Cross-modality generalization; uncertainty estimation	Unvalidated uncertainty clinically; higher memory
Han and Du (2025) (MHWT)	Single- and multi-modal MRI reconstruction	IXI, fastMRI, and BraTS2020	Hybrid Swin Transformer	Retractable and variable window attention with modality fusion	PSNR, SSIM, NMSE	IXI ×4: PSNR 38.96 dB, SSIM 98.16%	Strong modality fusion; long-range modeling	Windowed attention limits full global context
M.khair et al. (2026) (VRF-Net)	MPI system matrix recovery and reconstruction	Open MPI, simulated MPI	ViT + Residual Feature Network	Global ViT attention with local residual refinement	nRMSE, PSNR, and SSIM	PSNR 39.08 dB (matrix); 41.58 dB (image)	First transformer-based MPI recovery	No in vivo validation
Cao et al. (2025) (MBST)	4D-CBCT reconstruction	20 thoracic cancer patients	Masked Swin Transformer	Multi-interval sparse projection training	PSNR, SSIM, ME, MAE	Clinical SSIM 81.5%, PSNR 27.93 dB	Clinically validated; motion-robust	Limited cohort size
Zhang et al. (2025a) (CS-SwinGAN)	Multi-coil MRI reconstruction	fastMRI multi-coil	Swin Transformer GAN	CS pre-enhancement with dual-domain GAN supervision	PSNR, SSIM, and NMSE	Best PSNR/SSIM across acceleration factors	Noise-robust; strong detail recovery	GAN instability; heavy computation
Yang T. et al. (2025) (SVFR)	Efficient 3D MRI reconstruction	Brain MRI, Stanford knee	2D ViT + attention fusion	Slice-to-volume fusion across three planes	PSNR, SSIM, and NMSE	Higher PSNR with ~40% less memory	Memory-efficient volumetric modeling	Indirect 3D representation
Hou et al. (2025) (IGT-Net)	Accelerated MRI reconstruction	Public MRI datasets	ASAT Transformer + GMM + unfolding	ISTA-based transformer unrolling with statistical artifact modeling	PSNR, SSIM, NMSE, and RLNE	Best PSNR/SSIM across sampling ratios	Strong physics consistency	Higher inference time
Liu J. et al. (2024) (R3ST)	Fundus spectral reconstruction	Public multispectral fundus	Spectral Transformer + Retinex	Retinex-guided spectral attention learning	MAE, spectral error, and oximetry error	SOTA across cameras (scenario-based)	Cross-camera robustness	No unified benchmark
Chen et al. (2025) (DDFCA-Net)	Accelerated MRI reconstruction	CC359, fastMRI	Dual-path ViT + CNN	Cross-attention fusion between image and k-space	PSNR, SSIM, and NMSE	Best per mask and acceleration	True dual-domain attention	Single-coil only
Wang et al. (2023) (HybridF)	DCE-MRI super-resolution	Clinical DCE-MRI, IXI	Transformer + CNN hybrid	Multi-scale CNN–Transformer fusion	PSNR, SSIM	Reported average PSNR gains vs. CNN SR models	Dynamic MRI focus	No unified PSNR
Yuan et al. (2025) (WUTrans-MoCo)	Motion-compensated 4D-CBCT	Public lung datasets	Transformer + NeRF	NeRF-guided reconstruction with motion compensation	PSNR, SSIM, and MAE	Best across respiratory phases	Clinically relevant 4D modeling	Very heavy pipeline
Lin et al. (2024) (DdeNet)	Sparse-view CT reconstruction	Low-dose CT dataset	Pale-Transformer + CNN	Sinogram restoration + global image transformer	PSNR, SSIM, and RMSE	Best per sparse-view setting	Strong edge preservation	Single-dataset validation
Pan et al. (2025)	Real-time CBCT reconstruction	56 lung cancer patients	Diffusion Transformer (PC-DDPM)	Physics-guided patient-specific diffusion	PSNR, SSIM, and tumor error	High-fidelity real-time reconstruction	Ultra-sparse imaging	Patient-specific training
Wang Y. et al. (2024)	Low-dose PET reconstruction	Clinical PET–MRI	3D Transformer GAN	Multimodal fusion with edge-aware loss	PSNR, SSIM, and RMSE	Best across datasets (scenario-based)	Strong clinical relevance	GAN instability
Lyu et al. (2023)	Dynamic cardiac MRI reconstruction	Public cine MRI	Multi-view Transformer GAN	Spatio-temporal attention with region-focused loss	PSNR, SSIM	Best up to ×10 acceleration	Excellent temporal modeling	Dynamic-only
Zhang et al. (2023) (TIME-Net)	Dual-energy CBCT artifact removal	Simulated + animal data	Multi-encoder Transformer	Image, prior, and transformer fusion	PSNR, SSIM, RMSE	Superior across angle settings	Dual-energy awareness	Limited human data
Zou et al. (2025) (MMR-Mamba)	Multimodal MRI reconstruction	BraTS, fastMRI	Mamba + frequency fusion	Selective spatial–frequency integration	PSNR, SSIM, and NMSE	Highest PSNR/SSIM with fewer parameters	Efficient long-range modeling	Paired modalities required
Hosseinimanesh et al. (2025)	Dental crown mesh reconstruction	Clinical dental scans	Point Transformer	Point completion + DPSR	Chamfer, MSE	CD 12.32%, MSE 46.43%	Clinically critical margin modeling	Task-specific
Tu et al. (2025) (DiTMSR)	MRI super-resolution	Public + clinical MRI	Diffusion Transformer + Mamba	Latent diffusion with efficient decoding	PSNR, SSIM, and RMSE	SOTA across datasets (per-scale)	Strong global modeling	Diffusion inference cost

**Table 13:** Quantitative performance comparison of transformer-based medical image reconstruction methods.

References	Dataset/task	PSNR (dB)	SSIM (%)	Other metrics
Wang W. et al. (2025)	Sparse-view X-ray CT foot reconstruction (NAF dataset + TIGRE simulated projections)	32.88	94.09	FPS reported (not used for quality comparison)
Li Y. et al. (2025)	Sparse-view CT reconstruction – CT lymph node dataset (30 projections)	42.16	97.05	VIF = 0.6901
Huang J. et al. (2025)	Sparse-view CT reconstruction – Low-Dose CT abdomen subset	45.72	98.30	LPIPS = 0.037
Han and Du (2025)	Multimodal MRI reconstruction – IXI dataset, random ×4 undersampling	38.96	98.16	NMSE = 0.00255
Hou et al. (2025)	MRI reconstruction – IXI dataset (average over 5 sampling rates)	38.56	92.76	NMSE = 0.0466, RLNE = 0.1287
Cao et al. (2025)	4D-CBCT reconstruction – clinical evaluation	27.93	81.50	ME = −53.79, MAE = 73.77
Zhang et al. 2025a)	Multi-coil MRI reconstruction – fastMRI brain T2, Cartesian mask (30% sampling)	34.46	92.89	NMSE = 0.0052
Yang T. et al. (2025)	3D multi-coil brain MRI reconstruction (SVFR, ×4 acceleration)	33.72	93.48	NMSE = 0.0198, DSSIM = 0.0326
M.khair et al. (2026)	MPI system matrix recovery – Open MPI dataset (×2 scale)	39.08	83.50	nRMSE = 0.403
Yuan et al. (2025)	Sparse-view 4D-CBCT reconstruction—TCIA lung dataset (avg. over phases)	45.89	98.10	RMSE reported
Lin et al. (2024)	Sparse-view CT reconstruction—Mayo Clinic LDCT dataset (128 views)	38.89	92.16	RMSE = 0.0121
Liu J. et al. (2024)	RGB-to-multispectral fundus reconstruction (retinal oximetry, 4-fold CV)	37.22	NR	MAE_ROI_ = 3.36, RMSE_ROI_ = 4.25
Chen et al. (2025)	Accelerated MRI reconstruction – CC359 Brain, Gaussian-1D 30% sampling	38.25	96.22	NR
Wang et al. (2023)	DCE-MRI super-resolution – IXI brain MRI	33.37	95.00	NMSE = 0.01
Wang Y. et al. (2024)	PET reconstruction (LPET + T1-MRI → SPET, clinical dataset)	25.73	98.80	NMSE ≈ 0.0205
Lyu et al. (2023)	Cardiac cine MRI reconstruction (dynamic MRI, AF = ×10)	34.58	87.45	RMSE = 1.89 × 10^−2^
Zhang et al. (2023)	Limited-angle dual-energy CBCT reconstruction (quarter-scan setting)	39.66	96.94	MAE = 16.39 HU
Zou et al. (2025)	Multimodal brain MRI reconstruction—BraTS dataset (×4 acceleration)	40.98	98.50	NMSE = 0.00454
Pan et al. (2025)	Lung 4DCT → OSD-CBCT reconstruction	35.61 ± 2.90	93.00 ± 3.00	MAE = 25.13 HU, NCC = 0.99, FID = 0.26, LPIPS = 0.07
Tu et al. (2025)	fastMRI MRI super-resolution (×4)	34.51	88.24	FID = 105.0, LPIPS = 0.120
Hosseinimanesh et al. (2025)	Personalized dental crown mesh reconstruction from 3D point-cloud context	NR	NR	CD-L1 = 54.39, CD-L2 = 8.41, MSE = 0.0015

NR, Not Reported in the original study.

**Table 14:** Same-study non-transformer baseline comparisons in medical image segmentation.

Transformer-based or related model	Dataset/task	Reported model performance	Non-transformer baseline(s)	Reported baseline performance	Interpretation for fair comparison
TransUNet (Chen J. et al., 2024)	Multi-organ and tumor segmentation	Average Dice improvement: +1.06% for multi-organ segmentation; +4.30% for pancreatic tumor segmentation	nnU-Net	Reported as lower by 1.06% and 4.30% Dice in the corresponding tasks	The reported comparison is particularly informative because TransUNet is evaluated against nnU-Net, a strong and widely used non-transformer baseline for medical image segmentation. The observed Dice improvements therefore provide task-specific evidence that transformer-enhanced encoder–decoder designs may improve segmentation performance under comparable evaluation conditions.
UCTNet (Guo et al., 2024)	Synapse, ACDC, and ISIC2018 segmentation	Dice = 89.44% on Synapse; 92.91% on ACDC; 91.15% on ISIC2018	nnU-Net, D-Former, H2Former, ConvFormer	Synapse: UCTNet +2.45% over nnU-Net; ACDC: UCTNet +0.51% average Dice	The same-study evaluation across Synapse, ACDC, and ISIC2018 provides a useful basis for comparing UCTNet with CNN-based, transformer-based, and hybrid segmentation baselines. The reported improvements over nnU-Net suggest that the proposed design may offer added value, particularly when both local boundary information and broader anatomical context are required.
Med-SA (Wu J. et al., 2025)	Multimodal medical image segmentation; BTCV example	BTCV: Dice = 89.8%; HD95 = 2.18 mm	MedSAM, nnU-Net, Swin-UNETR, TransUNet, and CoTr	Compared with Swin-UNETR: +2.9% Dice; 9–11 mm HD95 reduction	The comparison indicates that adapting foundation-style segmentation models to medical imaging can improve both overlap-based and boundary-based performance in the evaluated setting. However, the interpretation should remain linked to the specific dataset, prompt strategy, adaptation procedure, and target anatomy, as promptable segmentation performance can vary across clinical contexts.
LightM-UNet (Liao et al., 2024)	LiTS 3D liver/tumor segmentation and 2D X-ray segmentation	LiTS: Dice = 84.58%; mIoU = 77.48%. 2D X-ray: Dice = 96.17%; mIoU = 92.74%	nnU-Net, SwinUNETR, UNETR, and U-Mamba	LiTS: +2.5% mIoU over nnU-Net; 47× fewer parameters and 15× lower computational cost. 2D X-ray: about +0.5% accuracy over U-Mamba with 99% fewer parameters	The results show that efficient Mamba-assisted or state-space-based segmentation architectures can provide competitive or superior performance while substantially reducing computational cost. This comparison is important because it demonstrates that transformer alternatives may be preferable in resource-constrained or volumetric segmentation settings when efficiency and accuracy are considered jointly.
Swin-UMamba (Liu J. et al., 2025)	AbdomenMRI, EndoVis, and microscopy segmentation	Strong multi-dataset performance reported	CNN-based, transformer-based, and Mamba-based comparators	–	The inclusion of CNN-, transformer-, and Mamba-based comparators enables a broader interpretation of segmentation performance across different architectural families. Swin-UMamba is best interpreted as a hybrid transformer-alternative model, and its results should be considered within the specific dataset, dimensionality, and validation protocols used in each benchmark.
nnU-Net Revisited (Isensee et al., 2024)	Large-scale 3D medical segmentation benchmarking	Well-configured CNN nnU-Net variants often match or outperform transformer and Mamba methods	CNN-based nnU-Net, ResNet/ConvNeXt nnU-Net variants, transformers, and Mamba models	–	This benchmark is methodologically important because it shows that carefully configured CNN-based segmentation frameworks remain highly competitive against transformer- and Mamba-based models. The finding emphasizes that architectural comparisons should account for tuning quality, training protocol, dataset scale, and implementation strength, rather than attributing performance differences solely to model family.
I2U-Net (Dai et al., 2024)	Skin lesion, polyp, brain tumor, and abdominal multi-organ segmentation	Superior performance and generalization over state-of-the-art methods	U-Net-derived and other segmentation baselines	–	I2U-Net provides a relevant non-transformer reference for assessing whether transformer-based designs offer consistent advantages over strong U-Net-derived segmentation models. Its inclusion highlights that well-designed convolutional or non-transformer architectures can still achieve strong generalization across heterogeneous segmentation tasks.
U-KAN (Li C. et al., 2025)	Medical image segmentation and generation	Higher accuracy with lower computational cost reported	U-Net-based segmentation backbones	–	The comparison illustrates that non-transformer architectural alternatives, including KAN-based designs, may improve segmentation accuracy and computational efficiency relative to conventional U-Net-style backbones. This supports a more balanced interpretation in which transformer-based models are one important direction, but not the only pathway for advancing medical image segmentation.

### 5.5 Medical image registration

Medical image registration deals with the alignment of fixed and moving images with the aim of comparing, merging or tracking anatomical and functional changes. This task differs from segmentation, classification, and reconstruction in terms of geometric nature and evaluation criteria, as its performance depends on the accuracy of the deformation field estimation, topology preservation, anatomical compatibility and the type of transformation used. Therefore, rigid, affine, and deformable registration methods should be interpreted separately. In this subsection, studies based on transformer and hybrid models of image registration are reviewed based on the type of transformation, modalities involved, how spatial correspondence is modeled, evaluation criteria and the extent of applicability in clinical scenarios.

In medical image registration, the reviewed studies are divided based on the type of geometric transformation and the nature of the alignment problem. Specifically, rigid, affine, and deformable registration methods should not be interpreted as a homogeneous group, as their clinical goals, evaluation criteria, and level of computational complexity differ. Therefore, the studies in Section 5.5 are categorized based on the type of registration, modalities involved, architectural design, use of cross-attention or spatial modeling, and metrics such as Dice, TRE, HD95, and SDlogJ.

#### 5.5.1 Distinguishing rigid, affine, and deformable registration methods

In interpreting studies related to medical image registration, it is necessary to distinguish between rigid, affine, and deformable registration, as these three families do not solve the same problem with the same level of complexity. In rigid registration, transformation is usually limited to translation and rotation, and the main goal is to achieve global alignment of two images without changing the geometric shape of the internal structures. In affine registration, in addition to translation and rotation, transformations such as scaling and shearing are also allowed, and therefore the model has a greater ability to align global geometric differences. In contrast, deformable registration seeks to model local and nonlinear spatial changes and is usually used to accommodate complex anatomical structures, interindividual variations, organ motion, or changes due to time and treatment.

Accordingly, the registration studies in this review are interpreted in three distinct groups. Rigid methods, or methods designed primarily for initial and global alignment, should not be directly compared to deformable methods such as TransMorph or XMorpher, because deformable methods typically have larger transformation spaces, more degrees of freedom, and different evaluation criteria. Similarly, affine registration falls somewhere in between, being more complex than rigid registration in terms of geometric flexibility but less restrictive than deformable registration.

In terms of performance evaluation, the reported metrics should also be interpreted within the same registration family. For example, metrics such as target registration error or landmark error are more appropriate for evaluating global alignment in rigid and affine registration, while in deformable registration metrics such as Dice similarity coefficient for structure overlap, Hausdorff distance for boundary error, Jacobian determinant for checking for unrealistic deformations, and folding rate for evaluating the validity of the deformation field are usually more important. Therefore, the performance values reported in the registration studies in this review are presented as descriptive and contextual evidence and should not be used to directly rank methods with different transformation families.

To avoid misinterpretation, the analysis of registration performance is organized by transformation type and methodological objective. This separation suggests that performance differences between studies may be due not only to model quality but also to differences in registration type, imaging modality, target anatomical structure, evaluation criteria, and problem difficulty level. Therefore, fair comparisons between registration methods are only tenable when methods are evaluated in the same transformation family, on the same data, with the same criteria, and under the same evaluation protocol.

The reviewed registration approaches are organized into four architectural categories: transformer-based segmentation models, hybrid CNN–Transformer models, transformer alternatives, and non-transformer comparative models.

Vision transformer for unsupervised volumetric medical image registration, presented in Chen et al. (2021) introduced the first ViT-based framework for volumetric (3D) registration of medical images. The model is designed to overcome the inherent limitation of convolutional networks in modeling long-range spatial relationships, since convolution operations only consider local dependencies in the image. The ViT-V-Net architecture has a hybrid structure of ConvNet and Transformer, in which high-level features are first extracted by convolutional layers and then fed into ViT as patch embeddings so that the network can learn long-range dependencies between volumetric image points. After processing in 12 alternating layers of multiple attention (MSA) and MLP, the output of ViT is returned to 3D space and reconstructed with a V-Net-style decoder. To avoid spatial information loss, long skip connections are used between the encoder and decoder, and a combined loss function of MSE and diffusion loss regularizer is used to ensure smoothness of the deformation field. Experiments on a brain MRI dataset (260 T1-weighted images) show that ViT-V-Net outperforms reference methods including SyN, NiftyReg, and two versions of VoxelMorph. The average Dice = 0.726 ± 0.13 is obtained for ViT-V-Net versus 0.713 for NiftyReg and 0.711 for VoxelMorph-2, while the GPU execution time is reported to be only 0.002 seconds for each pair of images. Also, the t-test statistical test confirms the significant difference in the performance of ViT-V-Net compared to other models (*p* < 0.001). Qualitative results showed that ViT-V-Net produces smoother and more accurate deformation fields than CNN-based methods and has better structural adaptation in complex regions such as ventricles and cortical areas.

#### 5.5.2 Transformer-based models

Medical image registration is a heterogeneous task, and the reviewed methods should not be interpreted as belonging to a single directly comparable family. Depending on the transformation model, registration methods can be broadly divided into rigid or initial alignment methods, affine registration methods, and deformable registration methods. Rigid registration estimates global translations and rotations and is mainly suitable for coarse alignment or cases in which anatomical deformation is limited. Affine registration extends this setting by allowing scaling, shearing, and other global linear transformations. In contrast, deformable registration estimates dense spatial displacement fields and is intended to model local, nonlinear anatomical changes. Because these transformation families differ in their degrees of freedom, clinical use cases, evaluation metrics, and expected difficulty, the following discussion separates them into distinct methodological categories rather than treating all registration results as directly comparable.

Given the methodological differences among rigid, affine, deformable, and diffeomorphic registration, Table 15 provides a transformation-aware categorization of representative registration methods and clarifies how their reported metrics should be interpreted within comparable task families.

**Table 15:** Transformation-aware categorization of representative medical image registration methods.

Model	Registration family	Transformation model	Architecture/method type	Domain/modality	Evaluation setting	Reported metrics	Interpretation and comparability note
TLBO–Affine–SURF–Projective (Arora et al., 2024)	Rigid/affine global alignment	Rigid transformation parameters followed by affine/projective refinement	Optimization-based and feature-based non-deep-learning method	Mono-modal and multimodal brain image registration	Whole Brain Atlas and Kaggle datasets	SSIM, MI, RMSE	Should be interpreted as a global alignment method. Its SSIM, MI, and RMSE values are not directly comparable with Dice- or Jacobian-based deformable registration results.
Patch-RegNet/ViT-Morph (Zhao et al., 2023)	Hybrid multi-stage registration	Whole-volume rigid registration, patch-based rigid refinement, and patch-based deformable registration	Hierarchical CNN–ViT registration framework	CT–MR and MR–MR head-and-neck radiotherapy images	Head-and-neck multimodal registration in adaptive radiotherapy	DSC	Should be interpreted as a multi-stage framework. Its rigid and deformable components should be discussed separately because each stage solves a different registration subproblem.
ViT-V-Net (Chen et al., 2021)	Deformable registration	Dense displacement field estimation	ViT encoder with V-Net decoder	3D brain MRI registration	Unsupervised volumetric registration	Dice, MSE, smoothness loss	Represents an early Transformer-based deformable registration model. It should be compared mainly with other dense deformation-field methods, not with rigid or affine-only approaches.
TransMorph (Chen et al., 2022)	Deformable/ diffeomorphic registration	Dense deformation field; diffeomorphic and Bayesian variants	Hybrid Transformer–ConvNet architecture	3D brain MRI and phantom-to-CT registration	Inter-patient and atlas-to-patient volumetric registration	DSC, Jacobian-based measures, uncertainty estimates	Appropriate for comparison with other deformable or diffeomorphic methods. Bayesian and diffeomorphic variants should not be mixed with simple affine registration results.
XMorpher (Shi et al., 2022)	Deformable registration	Dense deformation field guided by cross-attention correspondence	Cross-attention Transformer-based registration framework	Cardiac and/or volumetric medical image registration	Weakly or semi-supervised deformable registration settings	DSC, Jacobian/folding-related measures	Should be interpreted as a deformable correspondence-learning method. Its Dice and topology-preservation measures are not comparable with global similarity metrics from rigid alignment.
TransMatch (Chen Z. et al., 2024)	Deformable registration	Dense deformation field estimated through multilevel feature matching	Dual-stream Transformer with local window self- and cross-attention	Brain MRI registration	Unsupervised deformable registration on LPBA40, IXI, and OASIS	DSC, folding rate, runtime, memory	Designed for explicit deformable feature matching. Its performance should be compared with deformable methods such as VoxelMorph, ViT-V-Net, TransMorph, and SymTrans under comparable datasets.
SymTrans (Ma et al., 2022)	Deformable/ diffeomorphic registration	Dense displacement field and diffeomorphic registration	Symmetric Transformer-based U-shaped network with efficient attention	Medical image registration	Unsupervised image registration	Registration accuracy, deformation regularity	Should be treated as a deformable Transformer-based method. Its contribution is long-range cross-image modeling, not global affine alignment.
SparseMorph (Bai et al., 2024)	Hybrid affine + deformable registration	Initial affine alignment followed by deformable vector field prediction	Sparse Transformer encoder with CNN decoder and weak supervision	Mono-modal brain MRI and multimodal cardiac registration	IXI, OASIS, and MMWHS datasets	DSC, runtime, parameters	Contains an affine initialization component, but the main reported contribution is deformable registration. Its affine stage should not be interpreted as equivalent to final dense deformation performance.
LWSA registration (Li W. et al., 2024)	Deformable registration	Dense deformation field with wavelet-enhanced attention	Wavelet transform with linear self-attention	Brain MRI registration	LPBA40, IXI, and OASIS brain registration benchmarks	DSC, HD95, Jacobian-related measures	Belongs to deformable registration because it estimates local nonlinear alignment. Its results are comparable only with methods evaluated on similar brain MRI deformable-registration protocols.
SCT-Net (Liu X. et al., 2025)	Deformable registration	Dense deformable registration between radiotherapy CT image pairs	Multi-level self-cross Transformer registration network	Cervical cancer radiotherapy CT images	EBRT–ICBT deformable registration for dose accumulation	DSC, HD95	Task-specific deformable registration method. Its bladder and rectum DSC/HD95 values should not be generalized to brain MRI or rigid alignment tasks.
MCANet (Liu et al., 2026)	Deformable registration	Dense deformation field estimation	Hybrid multi-scale convolutional network with Swin Transformer bottleneck	3D brain MRI registration	OASIS, LPBA40, and IXI datasets	Registration accuracy/DSC-related measures	A hybrid deformable method combining local convolutional features and global Transformer context. It should be compared with other brain MRI deformable methods under the same datasets.
H-SGANet (Zhou and Cao, 2025)	Deformable registration	Dense deformation field estimation	Hybrid ConvNet–graph attention–Transformer framework	Volumetric brain MRI registration	OASIS and LPBA40 datasets	DSC, runtime, parameters	A deformable hybrid model emphasizing anatomical graph connectivity. Its efficiency and Dice results should be interpreted within brain MRI deformable registration only.
AutoFuse/AutoFuse-Trans (Meng et al., 2025)	Deformable registration	Dense deformation field with adaptive feature fusion	CNN-based and Swin-Transformer-based automatic fusion network	Brain MRI and cardiac MRI registration	Eight public datasets; inter-subject and intra-subject settings	DSC, TRE, Jacobian-related measures	Should be interpreted as a deformable fusion-based framework. The CNN and Transformer variants should be compared within the same dataset and transformation setting.
TCDE-Net (Yang X. et al., 2025)	Deformable registration	Dense nonlinear deformation estimation	Unsupervised dual-encoder network using Swin Transformer and ConvNeXt encoders	3D brain MRI registration	OASIS, IXI, and Hammers-n30r95 datasets	DSC, deformation regularity, task-specific registration metrics	A deformable hybrid architecture designed for complex nonlinear deformation. It should not be compared with rigid or affine-only methods.
WaveMorph (Zhang X. et al., 2025)	Deformable registration	Dense deformation field with topology-aware refinement	Wavelet-guided multi-scale ConvNeXt registration model	Brain MRI registration	IXI atlas-to-patient and OASIS inter-patient registration	DSC, runtime, topology-related measures	Transformer alternative rather than a pure Transformer method. It is useful as a deformable-registration comparator, not as evidence of Transformer-only superiority.
NIR (Sun et al., 2024)	Deformable/ diffeomorphic registration	Continuous displacement or velocity field through neural fields	Optimization-based neural-field registration framework	3D brain MRI registration	Mindboggle101 and OASIS datasets; cross-dataset evaluation	Surface Dice, volume Dice, Jacobian-related measures	A non-Transformer deformable alternative. It is useful for contextual comparison, especially for topology preservation and domain-shift robustness.
RDP-Net (Wang H. et al., 2024)	Deformable/ diffeomorphic registration	Recursive dense deformation estimation with diffeomorphic integration	CNN-based recursive deformable pyramid network	Brain MRI registration	LPBA, Mindboggle, and IXI datasets	DSC, HD, ASSD, Jacobian-related measures	Non-Transformer deformable comparator. It should be used to contextualize whether Transformer modules improve over strong CNN-based registration baselines.
uniGradICON (Tian et al., 2024)	Foundation-style deformable registration	General-purpose deformation model with inverse-consistency constraints	GradICON-based foundation registration model	Multi-anatomy, Multimodal medical image registration	Twelve public datasets; zero-shot and fine-tuning settings	DSC, TRE, HD, folding-related measures	A foundation-style registration method rather than a task-specific Transformer. Its broad evaluation makes it useful for generalization analysis, but it should not be mixed with single-dataset rigid or affine methods.
M2M-Reg (Choo et al., 2026)	Multimodal deformable registration	Dense deformation field guided by mono-modalized cyclic training	Framework-level approach compatible with existing deformable architectures	Extremely heterogeneous PET–MRI and FA–MRI registration	ADNI multimodal registration setting	DSC, Jacobian-related measures	A framework-level deformable method for heterogeneous modalities. It should be compared with deformable multimodal baselines, not with rigid, affine, or single-modality registration methods.
LessNet/Decoder-only registration (Jia et al., 2025)	Deformable/ diffeomorphic registration	Dense deformation estimation with decoder-only design	Lightweight decoder-only registration architecture	Multi-organ deformable medical image registration	Multiple public datasets across brain, cardiac, and abdominal settings	DSC, TRE, Jacobian, runtime	A lightweight deformable comparator. Its main relevance is efficiency and generalization, but its metrics remain specific to deformable registration settings.

The table separates registration methods according to transformation family to avoid misleading cross-family comparisons. Rigid and affine methods primarily evaluate global alignment, whereas deformable and diffeomorphic methods estimate local nonlinear transformations and require additional assessment of deformation regularity. Therefore, similarity-based metrics such as SSIM, MI, and RMSE should not be directly compared with deformable-registration metrics such as DSC, HD95, TRE, Jacobian determinant, or folding rate unless the methods are evaluated within the same transformation family, dataset, anatomy, and validation protocol.

##### 5.5.2.1 Rigid and initial alignment registration

Rigid and initial alignment methods are primarily designed to correct global misalignment between moving and fixed images. These methods are typically useful when the dominant spatial differences are caused by patient positioning, scanner geometry, acquisition orientation, or global motion rather than local anatomical deformation. In this setting, the transformation is usually limited to translation and rotation, and the goal is to produce a stable global correspondence that can either serve as the final registration output or as an initialization step for more flexible affine or deformable methods.

A representative example is the hybrid optimization-based method proposed by Arora et al. (2024). This approach combines teaching-learning-based optimization (TLBO) with feature-based alignment using SURF, KNN matching, RANSAC-based outlier removal, and projective transformation. Although this method is not a transformer-based deep learning architecture, it is useful as a non-transformer comparator for rigid or global alignment because it explicitly optimizes image similarity and feature correspondence without requiring training data. Its reported evaluation relies on similarity-oriented measures such as structural similarity, mutual information, and RMSE, which are more appropriate for global alignment assessment than for dense anatomical deformation analysis. Therefore, such results should not be directly compared with deformable registration methods that report Dice score, HD95, Jacobian determinant, or folding rate.

Rigid and initial alignment approaches remain clinically relevant because they can improve the robustness of subsequent deformable registration, particularly in multimodal settings or in cases with large initial spatial displacement. However, their main limitation is that they cannot adequately model local anatomical variability, organ motion, tumor deformation, or nonlinear inter-subject differences. For this reason, they should be interpreted as alignment or initialization methods rather than as alternatives to full deformable registration frameworks.

##### 5.5.2.2 Affine registration

Affine registration occupies an intermediate position between rigid alignment and deformable registration. Compared with rigid transformation, affine models can represent a broader range of global geometric differences by incorporating scaling, shearing, and anisotropic transformation. This makes affine registration useful for inter-subject alignment, scanner-dependent geometric variation, and preprocessing before dense deformable registration. However, affine methods still assume a single global transformation and therefore cannot capture local nonlinear anatomical changes.

Several learning-based registration frameworks include affine alignment either as an explicit component or as part of a multi-stage pipeline. For example, TransMorph was introduced as a hybrid Transformer–ConvNet model for volumetric medical image registration and includes variants related to affine and deformable registration (Chen et al., 2022). Similarly, some deformable registration pipelines first perform rigid or affine pre-alignment before estimating a dense deformation field. SparseMorph, for instance, incorporates an affine network for initial alignment before the subsequent deformable registration stage (Bai et al., 2024). In these cases, the affine component should be understood as a global transformation stage that improves initialization and reduces the burden on the deformable module, rather than as a direct equivalent of dense deformable registration.

The evaluation of affine registration should therefore be interpreted separately from deformable registration. Metrics such as mutual information, normalized cross-correlation, landmark error, RMSE, or similarity improvement may be meaningful for affine alignment, whereas Dice score or surface-distance metrics become more informative when anatomical labels or dense correspondences are available. Consequently, affine registration results in this review are treated as evidence of global alignment capability and preprocessing effectiveness, not as direct evidence of nonlinear anatomical correspondence.

##### 5.5.2.3 Deformable registration

Deformable registration represents the most flexible and technically demanding family of medical image registration methods. Unlike rigid or affine registration, deformable methods estimate dense displacement fields or diffeomorphic transformations that can model local anatomical variation, organ motion, disease-related deformation, and inter-subject structural differences. This family is particularly important in brain MRI registration, cardiac registration, radiotherapy planning, multimodal fusion, longitudinal analysis, and atlas-based segmentation.

Transformer-based and transformer-hybrid models have been widely explored in this family because attention mechanisms can capture long-range spatial dependencies between moving and fixed images. Early volumetric transformer-based approaches such as ViT-V-Net introduced patch-based Transformer encoding for 3D deformable registration (Chen et al., 2021). TransMorph further integrated Transformer modules with convolutional architectures for unsupervised volumetric medical image registration and introduced diffeomorphic and Bayesian variants to improve topology preservation and uncertainty estimation (Chen et al., 2022). XMorpher extended this direction by using cross-attention between moving and fixed images to explicitly model paired-image correspondence for deformable medical image registration (Shi et al., 2022). TransMatch also used a multilevel dual-stream feature-matching strategy to estimate deformation fields in unsupervised deformable registration (Chen Z. et al., 2024).

More recent studies have further diversified the deformable registration landscape. SymTrans uses a symmetric transformer-based architecture to model long-range cross-image relevance and directly predict deformation fields (Ma et al., 2022). SparseMorph improves computational efficiency through sparse attention and weak supervision for mono- and multimodal deformable registration (Bai et al., 2024). SCT-Net applies a multi-level self-cross transformer architecture to deformable registration in cervical cancer radiotherapy, where accurate registration between EBRT and ICBT images is required for dose accumulation (Liu X. et al., 2025). Other recent approaches, including wavelet-guided, ConvNeXt-based, neural-field-based, and foundation-style registration models, further show that transformer-based registration is increasingly being evaluated alongside transformer alternatives and non-transformer comparators rather than in isolation (Zhang X. et al., 2025; Sun et al., 2024; Tian et al., 2024).

Despite their strong performance, deformable methods should be interpreted with caution because their evaluation depends heavily on the target anatomy, dimensionality, modality, deformation magnitude, and regularization strategy. A high Dice score in atlas-to-brain registration is not directly comparable to a Dice score in multimodal cardiac or radiotherapy registration. Similarly, a method that improves overlap may still produce unrealistic deformation fields if topology preservation is not assessed. Therefore, deformable registration methods are discussed in this review as a separate family, and their reported performance is interpreted in relation to deformation regularity, anatomical plausibility, and task-specific validation.

##### 5.5.2.4 Interpretation of registration metrics

The performance metrics used in medical image registration are highly dependent on the transformation family and should not be directly compared across rigid, affine, and deformable methods. For rigid and affine registration, similarity-based metrics such as mutual information, normalized cross-correlation, structural similarity, RMSE, or landmark-based target registration error are commonly used to assess global alignment. These metrics indicate whether the moving and fixed images are globally better aligned, but they do not necessarily measure the quality of local anatomical correspondence.

For deformable registration, overlap and surface-distance metrics are more frequently reported when anatomical labels are available. Dice similarity coefficient evaluates the overlap between warped and reference anatomical structures, while HD or HD95 reflects boundary error and is often more sensitive to local misalignment. Additional deformation-quality metrics, such as the Jacobian determinant, folding rate, or the percentage of non-positive Jacobian values, are important for assessing whether the predicted deformation field is anatomically plausible and topology-preserving. In diffeomorphic or topology-preserving registration, these measures are particularly important because a high overlap score alone may not guarantee a physically meaningful transformation.

Accordingly, the quantitative results summarized in the registration tables and figures should be interpreted as descriptive, task-specific evidence rather than as a direct ranking across all registration methods. Comparisons are most meaningful when methods belong to the same transformation family, are evaluated on the same dataset, use the same preprocessing and validation protocol, and report the same metrics. In this review, rigid or initial alignment methods, affine methods, and deformable registration methods are therefore separated to avoid misleading cross-family comparisons.

Full Transformer for Deformable Medical Image Registration via Cross Attention, XMorpher, a full transformer framework for deformable medical image registration, was introduced in Shi et al. (2022). The framework is specifically designed to learn the cross-correlation between a moving image and a reference image. The XMorpher model consists of two parallel U-shaped subnetworks that simultaneously extract features from moving and fixed images and exchange information through cross attention transformer (CAT) modules. This design allows the network to gradually learn multi-level semantic correspondence between the two images. Unlike previous models such as Voxelmorph or TransMorph that used intra-image attention, XMorpher utilizes cross-attention between base and searching windows of different sizes, allowing for learning accurate local adaptations while optimizing computations. The model was evaluated in two unsupervised (VM-XMorpher) and semi-supervised (PC-XMorpher) learning frameworks on MM-WHS 2017 and ASOCA CT Heart data. Experimental results showed that in the unsupervised mode, VM-XMorpher with Dice = 83.0% and low field folding rate |*Jφ*| ≤ 0% = 3.15 has a significant advantage over Voxelmorph (80.2%) and TransMorph (81.1%). Also, in the semi-supervised mode, PC-XMorpher outperformed PC-Reg with Dice = 86.9% and showed the best topology preservation with minimal distortion |*Jφ*| ≤ 0% = 0.32. Qualitative results also confirmed the visual superiority of XMorpher in organ boundaries and more accurate alignment of cardiac structures. Component elimination analysis showed that the use of CAT blocks and multi-scale window segmentation significantly increased the registration accuracy and smoothness of the deformation field.

Chen Z. et al. (2024) proposed a model referred to as TransMatch, a transformer-based multilevel dual-stream feature matching network for unsupervised deformable image registration (DIR). The goal of this research was to combine the power of deep learning with explicit feature matching instead of traditional iterative optimization-based methods, which are usually time-consuming and rely on manual feature selection. In this framework, the TransMatch model has a symmetric dual-stream architecture in which the moving image and the fixed image are independently encoded in two separate branches. Each branch extracts multilevel features using a local window self-attention module based on the ST structure. Then, by introducing the key module local window cross-attention (LWCA), explicit matching between the features of two images at different levels (4 × 4 × 4 to 32 × 32 × 32) is performed. This mechanism enables the model to learn spatial relationships at local and global levels simultaneously and estimate the deformation field with high accuracy. To ensure the continuity and smoothness of the deformation field, a combined loss function including local normalized cross-correlation (LNCC) and L2 smoothing regularizer are used. Experiments are conducted on three brain MRI datasets LPBA40, IXI and OASIS, and the results show that TransMatch achieves Dice similarity coefficient values of 0.697, 0.761, and 0.794, respectively, which is superior to the advanced models TransMorph, ViT-V-Net, CycleMorph, and VoxelMorph. In addition, the deformation field folding rate |*Jφ*| ≤ 0 is reported to be almost zero (0.1%), indicating topology preservation and distortion prevention. In terms of efficiency, the model requires only a few tenths of a second on average for each image pair and its memory consumption is lower than previous models.

In another work, a multi-level self-cross Transformer architecture based registration network (SCT-Net) was developed (Liu X. et al., 2025). The model is said to be a deformable image registration method based on a multi-level self-cross Transformer architecture, specifically designed for the registration of CT scans from EBRT and ICBT in cervical cancer patients. Addressing the critical need for accurate dose accumulation in combined radiotherapy, SCT-Net employs parallel feature encoding subnetworks to extract multi-level registration-aware features from the fixed (ICBT) and moving (EBRT) images. These features are then processed by an enhanced Transformer architecture that fuses cross-attention and self-attention mechanisms to learn voxel-level correspondences. Following a dual-branch design similar to XMorpher, SCT-Net utilizes a shared selfcross Transformer to progressively align features and learn displacement information, which is subsequently decoded into the deformation field. Experimental results on 80 cervical cancer patients demonstrated that SCT-Net significantly outperformed VoxelMorph and XMorpher in terms of DSC and HD for bladder and rectum registration, highlighting its superiority for accurate deformable registration in this critical clinical application.

A symmetric Transformer-based framework known as SymTrans has been introduced for unsupervised medical image registration, providing an effective solution to the limitations of conventional CNNs (Ma et al., 2022). The model incorporates a Convolution-based Efficient Multi-Head Self-Attention (CEMSA) module designed to enhance local spatial awareness while reducing the computational overhead typically associated with standard Transformer architectures. By integrating CEMSA blocks within both the encoder and decoder of a U-shaped network, SymTrans is capable of modeling long-range spatial dependencies between moving and fixed images. The architecture directly predicts a dense deformation field, which is subsequently applied to warp the moving image toward alignment with the fixed reference. Experimental findings reported by Ma et al. (2022) demonstrate that this approach achieves state-of-the-art registration accuracy, validating the efficiency of the symmetric design and the effectiveness of the proposed attention mechanism.

#### 5.5.3 Hybrid CNN-transformer models

Zhao et al. (2023) presented patch-based registration network (Patch-RegNet), a hierarchical deep learning framework for deformable image registration (DIR), specifically targeting the complexities of CT-MR and MR-MR registration in head and neck radiotherapy. Recognizing the limitations of existing deep learning methods in such challenging anatomical regions, their approach integrates three sequential steps: initial whole-volume rigid registration, followed by a patch-based rigid registration to refine local alignment, and finally, a patch-based deformable registration achieved through their proposed ViT-Morph network. ViT-Morph uniquely combines CNNs and ViTs and exploits a modality-independent neighborhood descriptor for robust inter- and intra-modality registration. It is declared that based on the results obtained from Trained and validated on a dedicated head and neck patient dataset, Patch-RegNet demonstrated significant improvements in DIR accuracy, outperforming VoxelMorph and traditional architectures in terms of DSC for multiple critical organs at risk. In addition, it is stated that The hierarchical framework offers a robust and automated unsupervised solution for enhancing the precision of image registration in MR-guided adaptive radiotherapy for the head and neck.

The authors in Bai et al. (2024) introduced a framework called SparseMorph, a weakly supervised lightweight Transformer model for both mono- and multi-modal deformable image registration, aiming to improve computational efficiency and registration accuracy. To reduce complexity, they designed a sparse multi-head self-attention (SMHA) mechanism and a multi-branch multi-layer perception (MMLP) module for efficient feature capture. SparseMorph also incorporates an anatomically-constrained weakly-supervised strategy to guide the alignment of regions of interest. The architecture includes an affine network for initial rigid registration followed by a registration network with a Transformer-based encoder, a CNN-based decoder, and skip connections to predict the deformation vector field (DVF). Training is guided by a similarity loss and a weakly-supervised segmentation loss. Evaluated on mono-modal brain (IXI, OASIS) and multimodal cardiac (MMWHS) datasets, SparseMorph outperformed the state-of-the-art TransMatch with significant DSC score improvements while using considerably fewer parameters, demonstrating its efficiency and superior performance for clinical DIR applications.

The work in Meng et al. (2025) introduced an approach to medical image registration that is based on a data-driven fusion strategy and, unlike traditional experimental design-based methods (such as Early, Middle or Late Fusion), automatically optimizes where and how to fuse information during training. The presented model, called automatic fusion network (AutoFuse), has three U-Net-like branches that extract features from fixed and moving images separately and then combine them in a third branch called Fusion Branch by a module called fusion gate (FG). The FG module uses an attention mechanism to dynamically learn fusion weights to decide at each network level how much spatial information from each image to include in the alignment process. To increase the spatial perception range without significantly increasing memory, efficient large kernel (ELK) blocks are also used in the AutoFuse architecture. In addition to the CNN version, another version called AutoFuse-Trans is also introduced, which replaces convolutional blocks with ST to better model long-range dependencies. AutoFuse can be trained in both unsupervised and semi-supervised modes. In the unsupervised mode, a combined cost function consisting of normalized cross-correlation (NCC) and Kullback–Leibler Divergence is used, along with a Jacobian Determinant-based regularization component to ensure smoothness and invertibility. In the semi-supervised mode, an additional loss for the joint registration-segmentation loss is defined to also take advantage of weak anatomical labels. In extensive experiments on eight datasets, including inter-subject (3D brain MRI with OASIS, Buckner, and Mindboggle databases) and intra-subject (4D heart MRI with ACDC database) registration tasks, the AutoFuse model and its AutoFuse-Trans version achieved the absolute best performance in both cases compared to 14 reference methods including VoxelMorph, TransMorph, SEN, and AC-DMiR. In the unsupervised brain registration task, AutoFuse achieved an average of Dice = 80.8% and AutoFuse-Trans achieved 81.3%, while the rate of voxels with negative Jacobians remained < 0.05%. In the heart registration task, the model with Dice = 85.7% (and the transformer version with 86.2%) outperformed all competitors. Ablation studies showed that the design of the FG module was the main factor in improving performance, and the use of partially labeled data in semi-supervised training resulted in a 3%–6% increase in Dice. Furthermore, interpretation of the FG weight maps showed that AutoFuse adaptively tended to use more individual image features than combined information in deeper layers, indicating the model's deep understanding of local and global structural alignment.

TCDE-Net is proposed by Yang X. et al. (2025). The model is based on an unsupervised dual-encoder network designed to improve 3D brain medical image registration. The work tackeled the limitations in capturing local details, global context, and handling complex deformations. The technique leverages two complementary encoders, a ST and a ConvNeXt, in order to effectively extract both global and local features. By this it was intended to enhance the model's ability to manage large nonlinear deformations. A detail-enhancement attention (DEA) module is integrated to restore fine-grained features. This integration was crucial for registering intricate structures like gray-white matter boundaries. Besides, a deformation estimation and composition (DEC) module with skip connections aids in recovering multi-scale deformation features during the decoding stage. Relying on the evaluations on the OASIS, IXI, and Hammers-n30r95 3D brain MR datasets, it is claimed that TCDE-Net demonstrated superior performance and robustness compared to common registration techniques across various evaluation metrics. Moreover, it is reported that the obtained results highlighted the effectiveness of their dual-encoder approach for high-resolution brain image registration.

A lightweight hybrid sparse graph attention network (H-SGANet) for deformable medical image registration is employed in Zhou and Cao (2025). The developed model aimed to better represent anatomical connectivity and reduce computational costs compared to existing hybrid ConvNet-Transformer models. This network incorporates sparse graph attention (SGA), a mechanism based on vision graph neural networks (ViG) that utilizes predefined anatomical connections to expand the receptive field. To further capture long-range dependencies, Zhou and Cao introduced separable self-attention (SSA) integrated with depth-wise convolution in an SSAFormer module. H-SGANet, built on a U-Net architecture, processes images by extracting graph structured features using SGA in the encoder, followed by SSAFormer at the bottleneck. Skip connections facilitate multi-resolution feature fusion. The network optimizes moving and fixed images simultaneously through a hybrid feature fusion layer and end-to-end learning. Evaluations on the OASIS and LPBA40 datasets demonstrated significant Dice score improvements over VoxelMorph with a similar parameter count, highlighting the benefits of their hybrid SGA approach for volumetric medical image registration.

Chen et al. (2022) introduced TransMorph, a hybrid Transformer-ConvNet model for unsupervised volumetric medical image registration. Addressing the limitation of CNNs in capturing long-range spatial relationships, they leveraged the large receptive field of Vision Transformers for improved spatial correspondence learning between moving and fixed images. TransMorph incorporates Transformer architectures for both affine and deformable registration. Furthermore, the authors presented diffeomorphic and Bayesian variants of TransMorph to ensure topology-preserving deformations and provide registration uncertainty estimates, respectively. The authors claimed that the models were extensively validated on three distinct 3D medical imaging applications: inter-patient and atlas-to-patient brain MRI registration, and phantom-to-CT registration. It is further asserted that through comparative evaluations against existing registration methods and other Transformer-based architectures it is demonstrated that TransMorph achieves significant performance improvements, underscoring the efficacy of Transformer-based models for medical image registration tasks.

Hybrid multi-scale network for deformable brain medical image registration was introduced in Liu et al. (2026). The models is a hybrid and multi-scale network for unsupervised registration of brain MRI images that as claimed achieves high accuracy and topological stability in deformation fields by simultaneously utilizing local convolutional features and global transform dependencies. The MCANet architecture is designed based on the U-Net structure and consists of three main parts: an encoder, a transform bottleneck, and a decoder. In the encoder and decoder paths, a new module called multi-scale convolutional attention (MCA) is exploited, which combines three types of convolutions with different kernels—standard, depth-separable, and dilated—in parallel to simultaneously extract fine details, light channel information, and broad contexts of brain structures. This design allows the model to adapt multiscale features and adaptively highlight deformation regions. In the bottleneck section, a 3D ST is used to model long-range dependencies and accurately identify complex deformation regions. This combination of convolution and transformer ensures both high local accuracy and global structural understanding. For training, MCANet uses a hybrid loss function including MSE for image similarity and L2 regularization for deformation field smoothing, and is evaluated in unsupervised mode on OASIS, LPBA40, and IXI datasets. Quantitative results have shown that the model achieved Dice = 80.12% on the OASIS dataset, significantly improving (up to 9% in Dice) over CNN-based models such as VoxelMorph, CycleMorph, and transformer models such as TransMorph and ViT-VNet. Also, on LPBA40 and IXI datasets, the model outperformed TransMorph by 1.55% and 4.03%, respectively, and provided higher accuracy (over 13% improvement in Dice) in complex anatomies such as left-vessel and inferior lateral ventricle. Ablation experiments showed that both the MCA Block and ST bottleneck are essential to maintain accuracy under large deformations; removing each one results in a performance loss of about 1%–2%. The analysis of the attention regions, i.e., class activation maps (CAM), also showed that MCANet has a wider acceptance region and more accurate background coverage than the TransMorph model, which helps to better understand morphological changes.

#### 5.5.4 Transformer alternatives

In Li W. et al. (2024), a deformable medical image registration model leveraging wavelet transform and linear attention was developed. The algorithm aimed to address information loss during down sampling and disrupted anatomical integrity in transformerbased methods. Their approach utilizes the discrete wavelet transform to decompose images, providing richer features and preserving high-frequency information, thus mitigating down sampling-related losses. They introduce a linear wavelet self-attention (LWSA) module, combining wavelet transform with linear attention, which reduces computational complexity, expands the receptive field while maintaining structural integrity, and allows the model to focus differentially on various image regions. The proposed model adopts a U-Net architecture with LWSA modules in the encoder and decoder, along with skip connections and a spatial transformer network. Evaluated on three public brain imaging datasets, their method demonstrated improved registration performance compared to other models, highlighting the benefits of integrating wavelet transform and linear attention for effective deformable image registration.

#### 5.5.5 Non-transformer comparators

Non-transformer comparators establish important baselines and alternative paradigms through classical optimization, CNN designs, and novel mathematical frameworks.

The work in Hering et al. (2023) presented a challenge and a standardized data reference for the comprehensive evaluation of medical image registration algorithms. The aim of the study was to create a single platform for comparing deep, classical and hybrid learning methods on a wide range of intra- and inter-patient registration tasks. The Learn2Reg framework includes six key clinical tasks that cover a range of single-mode and multi-mode registrations (CT–CT, MR–CT, and MR–US) as well as inter- and intra-patient registrations in various body regions, including the brain, abdomen and lung. The dataset includes more than 65 implementations from 20 international teams that have been evaluated in a standardized and reproducible environment. For comprehensive evaluation, a set of complementary metrics such as accuracy (Dice, TRE), topological stability (SDlogJ), robustness (DSC30), and running time (RT) are employed, which provide a multidimensional analysis of the efficiency of the algorithms. The results of this challenge showed that the ConvexAdam method, by combining features extracted from UNet networks and inverse-consistent convex optimization, achieved the highest overall performance and was among the top three methods in all six tasks. After that, the Laplacian pyramid registration network (LapIRN) model achieved the best results by designing a multi-scale pyramid network in the OASIS and Hippocampus MR tasks. In contrast, classical methods such as corrField also provided competitive results in intra-patient registration despite not using labeled data. The MEVIS and PIMed methods also achieved stable and accurate performance on lung and abdominal tasks by combining classical optimization and deep learning. In addition to inter-algorithm comparison, this study investigated the effect of label bias, domain transfer, and the efficiency of unsupervised methods and showed that combining label-based learning with multi-scale architectures can minimize the gap between supervised and unsupervised methods. In terms of speed, the results showed that the classical GPU-accelerated methods are not much different from deep learning models in terms of execution time and can perform 3D registration in a few seconds. Overall, the Learn2Reg project is the first scalable and multi-task framework for the accurate evaluation of medical image registration algorithms, and by providing a standard dataset, rigorous benchmarks, and an open evaluation platform, it has created a new reference for future research in the field of deep learning-based medical image registration.

Introduced in 2024 as the first foundation model for medical image registration, uniGradICON (Tian et al., 2024) is a fundamental step toward combining the advantages of classical optimization-based methods and deep learning methods. While deep learning image registration networks (such as VoxelMorph and LapIRN) are fast and accurate but are trained only for specific tasks, uniGradICON was designed with the goal of creating a universal, fast, and generalizable model that can perform a variety of registrations without the need for retraining. It is based on the concept of gradient inverse consistency (GradICON), a weaker regularization that only guarantees invertibility, thus allowing a single model to be trained on a variety of data without the need to tune hyperparameters specific to each dataset. The uniGradICON architecture consists of a multi-stage network with four co-constructed U-Nets operating at different resolution levels (from 1/4 to full scale) and using the localized normalized cross-correlation (LNCC) similarity function with the GradICON regularizer to maintain the stability of the transformations. The model is trained on a combination of twelve general datasets including organs such as lung, brain, knee and abdomen and modalities such as CT, CBCT and MRI. In terms of performance, uniGradICON delivers results on par with or better than the classical and dedicated methods such as SyN and VoxelMorph on in-distribution tasks (for example, on the L2R-Abdomen dataset, the average Dice reached 78.9% with IO). In zero-shot scenarios, it is asserted that the model has achieved accuracy comparable to the top five competing Learn2Reg methods in registering extrapolated data from new anatomical sources or regions, and even outperformed them in some cases by implementing Instance Optimization (IO). In addition, finetuning uniGradICON on specific datasets (such as L2R-CBCT) has improved the accuracy of Dice by about 63.7%, surpassing the best available dedicated model.

The authors of Wang H. et al. (2024) presented an algorithm called recursive deformable pyramid network (RDP-Net), designed for unsupervised medical image registration, focusing on achieving high accuracy and reasonable computational efficiency in the presence of complex and bulky deformations. The model has a fully convolutional recursive pyramid structure and, unlike many recent methods, does not use heavy modules such as attention or transformer. The key idea of RDP-Net is to use a step-by-step recursive strategy from coarse-to-fine and gradually transfer high-level semantic information between different levels to estimate the deformation field in a logical, continuous, and accurate manner. In the proposed architecture, two separate encoders based on ResNet are used to extract features from fixed and moving images to better model independent features and different image styles. The pyramid decoder consists of three main modules: feature fusion, deformation estimation, and deformation dusion. At each level, the network performs several internal recursion to recursively refine the deformation field, and finally, by combining several partial fields, a continuous and topology-preserving deformation is obtained. To ensure the smoothness of the field, a diffeomorphic layer based on ordinary differential equation (ODE) and scaling and squaring integration is used. RDP-Net is tested on three public databases LPBA, Mindboggle, and IXI, and compared with twelve reference methods including VoxelMorph, TransMorph, PCNet, RDN, and SyN. The results show that the proposed model achieves the highest Dice score and the lowest Hausdorff distance and ASSD in all datasets. In particular, even on data without affine pre-alignment, RDP-Net achieved accuracy close to or better than manual pre-processing methods and reduced inference time by 33%. Component removal (ablation) experiments also showed that both multi-level regression and semantic transfer play a key role in improving accuracy.

Zhang X. et al. (2025) developed WaveMorph: wavelet-guided multi-scale convNeXt for unsupervised medical image registration, an approach for unsupervised medical image registration that significantly improves registration accuracy and computational efficiency by utilizing multiscale wavelet domain feature fusion and the ConvNeXt architecture. In this method, a module called multi-scale wavelet feature fusion (MSWF) is designed that uses Haar wavelet decomposition for lossless downsampling; this allows accurate spatial information and frequency features of images to be preserved simultaneously, allowing the network to align fine anatomical structures in high detail. In the reconstruction path, the authors have used a lightweight module called Dynamic Upsampling (DySample), which enables adaptive reconstruction of structures without introducing blurring or topological distortion. Thus, WaveMorph, by combining frequency domain representations and the spatial learning power of ConvNeXt, has been able to achieve high accuracy and geometric stability in multi-step registration tasks. Experimental results on brain MRI data (atlas-to-patient and inter-patient) showed that WaveMorph achieved Dice = 0.779 ± 0.015 and Dice = 0.824 ± 0.021, respectively, while its inference time was only 0.072 seconds per image. These values show better performance in both accuracy and speed compared to CNN- and Transformer-based models. In addition, topological analyses indicate that the model is able to preserve complex structures without distortion and is robust to noise and intensity differences. It is declared that WaveMorph, by utilizing multiscale wavelet domain fusion and the optimized ConvNeXt architecture, provides a lightweight, accurate, and suitable approach for real-time registration of medical images and is an important step toward the development of fast and topology-preserving methods for deformable registration.

In Choo et al. (2026), the authors presented a framework for unsupervised registration of multimodal images, specifically designed for cases with extreme heterogeneity between modalities, such as PET–MRI and FA–MRI. In these situations, conventional multimodal similarity measures such as LNCC or MI lose the ability to detect true alignment and cause image distortion. To solve this problem, the proposed multi-to-mono registration (M2M-Reg) model rewrites the multimodal registration problem into a monomodality problem and uses the monomodality similarity in a cyclic learning framework to guide the network. At each training step, a pair is sampled as a bridge between two modalities to create a cyclic structure, and the model indirectly learns intermodal relationships from intramodal paths. In addition, the authors present a new regularizer called gradient cycle consistency (GradCyCon), which uses Jacobian to combine cyclic mappings, guaranteeing smoothness and invertibility of the transformation, and enhances the diffeomorphic behavior of the network. In addition to fully unsupervised learning, this framework also has semi-supervised learning capabilities and can use pre-aligned pairs as “training bridges” to inject real mapping knowledge into the training process; without the need for label masks or reference maps. In experiments on the ADNI4 database of 350 positron emission tomography (PET)–MRI and 280 fractional anisotropy (FA)–MRI pairs, the M2M-Reg model was evaluated based on different architectures such as TransMorph, CorrMLP, and GradICON. The authors declared that their quantitative results showed that using M2M-Reg resulted in a twofold increase in the Dice index and an almost complete reduction of points with negative Jacobians (%∣*Jϕ*∣ < 0 ≈ 0), while the baseline models suffered from severe geometric distortions. Furthermore, even using only 5% of the pre-aligned pairs resulted in an improvement of more than 5% in Dice, indicating the high efficiency of the semi-supervised design.

In Sun et al. (2024), a framework called neural image registration (NIR) for medical image registration was introduced, which attempts to bridge the gap between classical optimization-based methods and fast learning-based methods. The main idea of NIR is that instead of defining the transformation on a discrete 3D network, the deformation field is modeled as a continuous function using “neural fields” or coordinate-capable MLPs; that is, the network maps a 3D coordinate to a displacement vector (for conventional deformable registration) or a velocity vector (for diffeomorphic registration) at each call, and if necessary, the final field is obtained by solving an ordinary differential equation, i.e., Neural ODE. The authors support two paths within the NIR unitary framework: (1) direct displacement field generation for general registrations and (2) velocity field generation and integration for topology-preserving registrations, the latter of which provides smooth and invertible transformations by ensuring that the Jacobian determinant is positive. To make the optimization truly practical and cost-effective, the paper proposes a two-stage optimization design with hybrid coordinate sampling: in the first phase, coarse, and fast alignment is performed with downsize sampling, and in the second phase, fine-tuning areas and local distortions are controlled with mini-patch sampling; this design allows both high registration accuracy and low proportion of points with negative Jacobians. NIR has been tested on two 3D brain MRI datasets, Mindboggle101 and OASIS, and in both cases, it has shown performance comparable to or better than classical methods such as SyN and NiftyReg, as well as several advanced learning-based methods (VoxelMorph, TransMorph, and XMorpher) in terms of both surface and volume Dice and the rate of points with negative Jacobian (**J** ≤ 0), while having lower optimization time and GPU consumption. More importantly, in the out-of-domain registration scenario (Mindboggle vs. OASIS), where many learning-based methods suffer from domain shift, NIR maintains its accuracy and regularity by performing the registration as a pairwise optimization on the continuous neural field, and is introduced as a flexible, data-insensitive, and topology-preserving solution for 3D medical image registration.

Arora et al. (2024) have presented a hybrid and optimized method for medical image registration that aims to achieve high accuracy in registering single-modal and multimodal images with minimal error and maximum similarity. In this method, first, image noise is removed using a Gaussian filter and then intensity normalization is performed. The next step involves finding the optimal parameters of the rigid transformation through the teaching-learning-based optimization (TLBO) algorithm, which is designed with the objective function of maximizing mutual information (MI). This algorithm has faster convergence than methods such as PSO and GA due to the lack of complex control parameters. To improve the final accuracy, the authors have added feature-based methods to the optimization phase. Key features are extracted using the speeded-up robust features (SURF) algorithm and then aligned using k-nearest neighbors (KNN). Random sample consensus (RANSAC) is used to remove false pairs, and finally projective image transformation is applied to correct the alignment. This hybrid approach, called TLBO–Affine–SURF–Projective, is tested on two databases, Whole Brain Atlas and Kaggle, and the results show that the proposed model performs much better than the reference methods, including particle swarm optimization (PSO), gray wolf optimization (GWO), and progressive images and SURF (PI-SURF) algorithm. Specifically, the structure similarity index measure (SSIM) rate is improved by 8%, the MI rate is improved by 12.71%, and the RMSE is reduced by 41.44% in multimodal images. The key advantage of this research is the intelligent combination of TLBO with robust features against intensity and noise changes (SURF-RANSAC), which leads to accurate, fast, and stable registration of medical images.

An architecture called tumor-aware recurrent registration (TRACER) for deformable image registration between patients with lung cancer was presented in Jiang et al. (2025). The method is specifically designed to align CT images between different patients to prevent unrealistic deformations in tumor areas while preserving the topology of healthy tissues. The TRACER model is based on a multi-stage 3D convolutional LSTM network in which deformations are calculated gradually and recursively to capture large anatomical differences between patients without losing local details. The network input includes 3D fixed and moving images along with tumor segmentation maps, which, with the Tumor Conditioning mechanism, allows the model to separately consider the features of the tumor region and suppress abnormal deformations around it. In addition, TRACER uses bidirectional tumor rigidity constraints, an intensity similarity loss function, and a deformation smoothness constraint to preserve the tumor geometry and tissue structure. Each stage of the LSTM includes a spatial Transformer network that integrates the velocity field into a Diffeomorphic Flow and preserves the topology. The model is trained completely unsupervised and uses more than 32,000 pairs of CT images of patients with non-small cell lung cancer (NSCLC). In three evaluation datasets, including Dataset I (308 image pairs), Dataset II (756 pairs), and Dataset III (42 patients with therapeutic dose data), the performance of TRACER was compared with several leading methods: PACS, FastSym, TransMorph, and SyN. The results showed that TRACER performed best in tumor preservation, with the lowest tumor volume difference (Δ*T*= 0.24% in Dataset I and 0.13% in Dataset III) and the lowest mean squared intensity error (MSE = 0.004–0.005). In addition, it also performed superiorly in tumor radiation dose preservation (dose difference of only 0.01–0.013 Gy), while the calculation time per registration was less than 10 s. Ablation studies showed that removing tumor conditioning completely eliminated tumor geometry and impossible physical deformations, while combining multi-step CLSTM with bilateral tumor conditioning resulted in topological stability and higher accuracy in healthy tissue registration. It was also found that using multi-step CLSTM enhanced feature activation in regions with structural differences (such as heart and tumor) and improved multi-scale alignment.

The RS-MOCO, i.e., regularized semi-supervised motion correction model, introduced in Huang C. et al. (2025), provides a deep learning-based framework for motion registration and correction in cardiac T1 mapping, focusing on topology preservation, geometric stability, and accuracy in the presence of large contrast variations. The model consists of three complementary networks: AffN for initial rigid alignment, RegN for accurate deformation registration, and SegN for semi-supervised pseudo-realistic label generation that provides better guidance to RegN. The key innovation in RS-MOCO is the introduction of the topology-based bidirectional and local orientation consistency (BLOC) constraint, which prevents field folding and anatomical structures by checking the bidirectional consistency of transformations and local control of the Jacobian determinant. In addition, the weighted loss (WLs) composite loss function is designed with an adaptive combination of four similarity measures (NCC, MI, NGF, and MIND) to simultaneously learn intensity and spatial structure information, and the CBAM channel-spatial attention module is also used to highlight key regions in the network. It is reported that evaluations on T1Dataset210 and SZC-T1 data showed that RS-MOCO outperforms reference methods such as SyN, VoxelMorph, DisQ, and Cb-Reg. The model achieved Dice = 0.827 and HDendo = 6.19 px on the original dataset, while reducing the field folding rate to less than 0.9%, while its inference time was only 8 seconds (vs. more than 376 seconds for SyN). Also, in the out-of-domain data, the registration accuracy was maintained at Dice = 0.809, and the qualitative results showed that after motion correction, the uniformity of signal intensity in the myocardial tissue and the clarity of the endocardial and epicardium boundaries were significantly improved. In summary, RS-MOCO, by combining semi-supervised learning, BLOC topology-based constraint, WLs weighted loss function, and dual-domain attention mechanism, has been able to provide an accurate, stable, and interpretable model for motion correction and registration of multi-contrast cardiac images, and is considered one of the most efficient learning-based frameworks in the field of cardiac medical image registration in recent years.

In the paper “Decoder-Only Image Registration” (Jia et al., 2025), the authors presented a framework for unsupervised 3D medical image registration, LessNet, that, unlike conventional encoder-decoder architectures, only includes a learnable decoder and replaces the entire encoder section with hand-designed features. LessNet uses three simple pooling operations (max, min, and average) to extract multiscale features from pairs of fixed and moving images and feeds these features directly into a four-section hierarchical decoder to learn the dense displacement field. This design completely eliminates the learnable parameters in the encoder, significantly reducing the number of parameters and memory required. In addition, a version of Diff-LessNet is also presented that is able to predict diffeomorphic fields by estimating static velocity fields and applying scaling and squaring operators. LessNet was evaluated on five reference datasets, including OASIS-1 (interpersonal brain), IXI (patient-to-patient atlas), ACDC (ES-ED heart), CHAOS (abdominal MRI), and a large multisite set of 13 databases. The results showed that LessNet provided comparable or better performance with only about 2% of the TransMorph parameters and less than half the memory of VoxelMorph. Using the default LessNet16 configuration, Dice ≈ 0.761 on OASIS and ≈ 0.765 on IXI.Diff-LessNet was also able to show more stable performance than the reference methods in preserving topology and avoiding folds (|*Jφ*| < 0 ≈ 0%). Statistical analysis (Wilcoxon test) did not show a significant difference between LessNet and advanced models such as TransMorph or LKU-Net, but a significant reduction in the number of parameters (more than 98% fewer) and training time (5 times faster) was reported. In addition, the results of the comparison with the “encoder-only” version (Slicer Network) confirmed that the optimized decoder in LessNet is able to provide performance on par with full networks without the need for a learnable encoder. As claimed, LessNet offers a new perspective on the field of medical image registration; this model shows that increasing the network size does not necessarily lead to improved performance, but rather that efficiency, accuracy, and topological stability can be improved simultaneously by focusing on designing compact and decoder-driven networks.

Table 16 provides a comprehensive overview of deep learning-based medical image registration methods, in which different approaches are systematically reviewed and compared in terms of architectural designs, alignment strategies, application areas, performance evaluation criteria, and distinctive features. Besides, Table 17 demonstrates a quantitative benchmark of transformer-based medical image registration methods, in which the performance evaluation indicators are compiled exactly based on the values reported in the original sources and adjusted to allow for an accurate, transparent, and systematic comparison between different approaches. In addition, Table 18 summarizes peer-reviewed comparisons between transformer-based methods or related architectures and non-transformer baselines in medical image registration.

**Table 16:** Comprehensive benchmark of transformer-based medical image reconstruction methods.

References	Application/ task	Dataset(s)	Transformer type/ architecture	Key methodology	Evaluation metrics	Best reported results	Strengths	Limitations
Chen Z. et al. (2024) (Trans- Match)	3D deformable brain MRI registration	LPBA40, IXI, and OASIS	Dual-stream Transformer with multi-level cross-attention	Explicit multilevel feature matching using windowed cross-attention between moving and fixed streams	DSC, TRE, HD, SDlogJ	LPBA40 DSC ≈ 0.697 ± 0.020; IXI DSC ≈ 0.764 ± 0.023; OASIS DSC ≈ 0.815 ± 0.023; TRE ≈ 1.62 mm	Explicit inter-image matching; strong global correspondence modeling; consistent SOTA on brain MRI	High memory cost; mono-modal MRI only; no tumor-aware modeling
Hering et al. (2023) (Learn- 2Reg)	Multi-task benchmark for inter-/intra-patient registration	Brain MR, Abdomen CT, Lung CT, and MR–US	Benchmark platform (CNN, ViT, hybrid methods)	Unified evaluation of >65 methods across anatomies and modalities	DSC, TRE, HD95, SDlogJ, runtime	Best teams achieved DSC ~0.82 (brain), TRE < 2 mm (lung), SDlogJ ~0.07	Largest public benchmark; standardized metrics; robustness and plausibility analysis	Not a single method; results vary strongly per task
Jiang et al. (2025) (TRACER)	Tumor-aware inter-patient CT registration	Lung cancer CT (3 datasets, >1000 pairs)	Recurrent 3D ConvLSTM (non-Transformer)	Progressive recurrent deformation with tumor masks and bidirectional rigidity losses	Tumor volume diff., MSE, dose error	Tumor volume diff. 0.13%–0.40%; dose error ≈0.01 Gy	First tumor-aware DIR; preserves tumor geometry; clinically relevant evaluation	Requires tumor masks; higher training complexity; not Transformer-based
Chen et al. (2022) (Trans- Morph)	3D deformable MRI and CT registration	IXI, OASIS, brain atlas, and XCAT-CT	Swin Transformer encoder + CNN decoder	Long-range dependency modeling with diffeomorphic and Bayesian variants	DSC, TRE, HD, and Jacobian	IXI DSC ≈ 0.754 ± 0.124; TRE ≈ 1.4 mm; SDlogJ ≈ 0.09	First major Transformer registration framework; uncertainty modeling	Heavy architecture; high GPU demand; limited clinical validation
Wang H. et al. (2024) (RDP)	Large-deformation brain MRI registration	LPBA40, Mindboggle, IXI	Pure CNN recursive pyramid	Coarse-to-fine recursive deformation refinement	DSC, ASSD, HD, Jacobian	DSC up to 0.832 ± 0.015 with reduced folding	Excellent large-deformation handling; lightweight; no affine pre-alignment	No global attention; weaker long-range modeling; not Transformer-based
Zhang X. et al. (2025) (WaveMorph)	Unsupervised brain MRI registration	IXI, OASIS	ConvNeXt + wavelet-guided fusion (Transformer-inspired CNN)	Wavelet-based lossless downsampling with dynamic upsampling	DSC, HD, Jacobian, and runtime	IXI DSC 0.779 ± 0.015; OASIS DSC 0.824 ± 0.021; 0.072s/image	Preserves high-frequency anatomy; real-time capable	Not a true Transformer; brain-only evaluation
Jia et al. (2025) (LessNet)	Multi-organ unsupervised deformable registration	13 public datasets (brain, cardiac, and abdomen)	Decoder-only CNN (non-Transformer)	Handcrafted multiscale pooling with learnable decoder only	DSC, TRE, Jacobian, runtime	Brain DSC ≈ 0.765; NPJ ≈ 0.148% with minimal parameters	Extremely lightweight; fast; strong generalization	Weaker global context modeling
Tian et al. (2024) (uniGrad- ICON)	Foundation model for general medical registration	12 datasets across modalities	Multi-stage GradICON U-Net	Unified training with gradient inverse consistency	DSC, TRE, HD, foldings	Consistent SOTA or near-SOTA across datasets; strong zero-shot ability	First foundation registration model; cross-modality robustness	Very large training cost; not Transformer-based
Choo et al. (2026) (M2M-Reg)	Extremely heterogeneous multimodal registration	ADNI PET–MRI, FA–MRI	Framework-level, model-agnostic	Cyclic mono-modal similarity training with GradCyCon regularization	DSC, Jacobian	Up to 2× higher DSC than SOTA on PET–MRI	Solves extreme multimodal mismatch; architecture-independent	Complex pipeline; tested mainly on brain
Arora et al. (2024)	Rigid/affine multimodal registration	Whole Brain Atlas, Kaggle datasets	TLBO + SURF–RANSAC (non-DL)	Feature-based optimization with projective alignment	SSIM, MI, RMSE	SSIM +8%, MI +12.7%, RMSE −41.4%	Explainable; no training required	Not deformable; not scalable to complex anatomy
Li W. et al. (2024) (LWSA)	Deformable brain MRI registration	LPBA40, IXI, OASIS	CNN + Linear Wavelet Self-Attention	Wavelet decomposition replaces downsampling; linear attention	DSC, HD95, Jacobian	OASIS DSC ≈ 0.83; HD95 improved vs TransMorph	Global receptive field with reduced attention cost	Brain-only; heavier than CNN baselines
Chen et al. (2021) (ViT-V-Net)	3D brain MRI deformable registration	In-house T1 MRI (260 volumes)	ViT encoder + V-Net decoder	Patch-based ViT models long-range dependencies	Dice, MSE, and smoothness	Dice 0.726 ± 0.130; >0.1 gain over affine	First ViT volumetric registration model	High memory; coarse patching
Shi et al. (2022) (XMorpher)	General deformable registration	Brain MRI benchmarks	Full Transformer with cross-attention	Dual parallel encoders exchange features via CAT blocks	DSC, HD	+2.8% DSC over VoxelMorph	Explicit correspondence modeling	Very heavy; memory intensive
Liu X. et al. (2025) (SCT-Net)	Clinical EBRT–ICBT CT registration	80 cervical cancer patients	Multi-level self-cross Transformer	Voxel-wise correspondence via self- and cross-attention	DSC, HD95	Bladder DSC 0.76; Rectum DSC 0.54; HD95 improved	Clinically validated; task-specific	Limited generalization
Liu et al. (2026) (MCANet)	Deformable brain MRI registration	OASIS, LPBA40, IXI	Hybrid CNN + Swin Transformer bottleneck	Multi-scale convolutional attention with global modeling	DSC, HD95, TRE	DSC gains up to +6.69% vs. baselines	Good balance of local and global modeling	Brain-only evaluation
Ma et al. (2022) (SymTrans)	Unsupervised deformable registration	LPBA40, IXI, and OASIS	Symmetric CNN–Transformer with CEMSA	Transformer blocks at multiple resolutions	DSC, HD, and SDlogJ	OASIS DSC ≈ 0.83; strong improvement over VoxelMorph	Multi-resolution Transformer usage; diffeomorphic variant	Higher complexity than CNN
Sun et al. (2024) (NIR)	Optimization-based deformable registration	2 brain MRI datasets	Neural fields + Neural ODE	Continuous deformation optimized per image pair	NCC, Dice, and regularity	Superior cross-dataset accuracy vs. CNNs; better regularity than SyN	Excellent generalization; continuous formulation	Not feed-forward; slower inference
Yang X. et al. (2025) (TCDE-Net)	3D brain MRI registration	OASIS, IXI, Hammers-n30r95	Dual encoder: Swin Transformer + ConvNeXt	Global–local fusion with detail-enhanced attention	DSC, HD95, and TRE	Highest DSC across all datasets; outperformed TransMorph	Strong balance of global/local modeling	Heavier; brain-only
Zhao et al. (2023) (Patch-RegNet/ViT-Morph)	Multimodal CT–MR & MR–MR registration	242 CT–MR + 213 MR–MR H&N cases	CNN + Vision Transformer hierarchy	Whole-volume to patch-level rigid/deformable alignment	DSC (7 organs)	CT–MR +6% DSC; MR–MR +4% DSC	Transformer-based multimodal DIR; clinical relevance	Patch pipeline complexity
Zhou and Cao (2025) (H-SGANet)	Deformable brain MRI registration	OASIS, LPBA40	Hybrid ConvNet + Graph NN + SSA-Transformer	Anatomical connectivity modeling via sparse graph attention	DSC, runtime, parameters	+3.5% DSC on OASIS with only 0.38M params	Excellent accuracy–efficiency trade-off	Complex design; brain-only
Huang C. et al. (2025) (RS-MOCO)	Cardiac T1 motion correction	Clinical cardiac T1-mapping	CNN + dual-domain attention (non-Transformer)	Topology-preserving consistency with anti-folding constraints	Dice, Jacobian	Significant myocardium Dice gain; near-zero folding	Clinically validated; topology-aware	Task-specific; no global Transformer
Bai et al. (2024) (SparseMorph)	Mono- and multimodal deformable registration	IXI, OASIS, MMWHS	Sparse Transformer encoder + CNN decoder	Sparse multi-head attention with anatomical constraints	DSC, runtime, parameters	Up to +11.4% DSC on cardiac multimodal; ~33% params of TransMatch	Strong accuracy–efficiency trade-off; multimodal support	More complex training
Meng et al. (2025) (AutoFuse/AutoFuse-Trans)	General deformable registration	8 public datasets	CNN + Swin Transformer variant	Data-driven fusion gates for feature alignment	DSC, TRE, Jacobian	Outperformed SOTA across inter- and intra-patient tasks	Architecture-agnostic; strong generalization	More parameters; harder to interpret

**Table 17:** Quantitative benchmark of transformer-based medical image registration methods (metrics as reported).

References	Dataset/task	DSC ↑	MSD/ASD ↓	HD/HD95 ↓	NCC/MI ↑	NPJ ↓	Time (s) ↓
Chen Z. et al. (2024) (TransMatch)	Brain MRI registration (LPBA40, IXI, OASIS)	LPBA40: 0.697 ± 0.020; IXI: 0.764 ± 0.023; OASIS: 0.815 ± 0.023	NR	HD95–LPBA40: 6.404 ± 0.727; IXI: 3.002 ± 0.558; OASIS: 1.906 ± 0.486	NA^*a*^	NR	NR
Wang H. et al. (2024) (RDP)	IXI–3D brain MRI deformable registration (affine pre-aligned)	0.832 ± 0.015	ASSD: 0.73 ± 0.06 mm	HD95: 2.24 ± 0.16 mm	NA^*a*^	<0.01%	0.68
Chen et al. (2022) (TransMorph)	IXI–atlas-to-patient 3D brain MRI registration	0.754 ± 0.124	NR	NR	NA^*a*^	0.396 ± 0.240%	NR
Jiang et al. (2025) (TRACER)	Inter-patient thoracic CT registration (lung cancer)	Lung: ≈ 0.92 ± 0.03	NR	HD95 reported (lung/heart/spinal cord; second-best)	NR	NA^*b*^	NR
Hering et al. (2023) (Learn2Reg)	OASIS–inter-patient 3D brain MRI registration (Task 3)	0.82	NR	HD95: 1.67 mm	NR	SDlogJ: 0.07	NR
Jia et al. (2025) (LessNet)	IXI–atlas-to-subject 3D brain MRI registration	0.765 ± 0.128	NR	NR	NA^*a*^	0.148 ± 0.076%	0.385 (GPU)
Zhang X. et al. (2025) (WaveMorph)	IXI–atlas-to-patient 3D brain MRI registration	0.779 ± 0.015	NR	NR	NA^*a*^	FR: 1.310 ± 0.313%	0.072/image
Arora et al. (2024)	Brain CT/MR mono- and multi-modal registration	NR	MSD: 0.0047 (best case)	NR	MI up to 12.83; NCC up to 0.98	NR	NR
Choo et al. (2026) (M2M-Reg)	ADNI PET → MRI brain multimodal registration	0.676	NR	NR	NA^*a*^	0.000%	NR
Tian et al. (2024) (uniGradICON)	IXI–atlas-to-patient 3D brain MRI registration	0.706	NR	NR	NA^*a*^	7.4 × 10^−3^	NR
Shi et al. (2022) (XMorpher)	MM-WHS 2017 + ASOCA–whole-heart CT registration	0.830 ± 0.047	NR	NR	NR	3.15 ± 0.79%	NR
Chen et al. (2021) (ViT-V-Net)	Brain MRI atlas-to-patient deformable registration	0.726 ± 0.130	NR	NR	NA^*a*^	0.381 ± 0.102%	0.002
Liu et al. (2026) (MCANet)	OASIS–inter-patient 3D brain MRI registration	0.8012 ± 0.0296	NR	HD: 11.879 mm; HD95: 2.053 mm	NA^*a*^	0.30 ± 0.11%	NR
Li W. et al. (2024) (Wavelet + Linear Attention)	OASIS–inter-patient 3D brain MRI registration	0.896 ± 0.016	NR	HD: 1.380 ± 0.089 mm	NA^*a*^	1.292 ± 0.196%	0.43
Liu X. et al. (2025) (SCT-Net)	EBRT ↔ ICBT CT pelvic registration (80 cervical cancer patients)	Bladder: 0.76 ± 0.04; Rectum: 0.54 ± 0.12	NR	HD95–Bladder: 4.4 ± 1.1 mm; Rectum: 5.9 ± 2.4 mm	NR	NR	2.80/sample (GPU)
Sun et al. (2024) (NIR-H-Diff)	Brain MRI registration (Mindboggle101, OASIS)	0.9071 ± 0.034 (surface DSC @1 mm, OASIS)	NR	NR	SSIM: 0.7907 ± 0.0074	$J_{\leq 0}:4.55\times 10^{-6}$	~640/pair
Ma et al. (2022) (SymTrans)	OASIS–inter-patient 3D brain MRI registration	0.757 ± 0.025	NR	NR	NA^*a*^	1485.659 ± 587.560	NR
Zhou and Cao (2025) (H-SGANet)	OASIS–inter-patient 3D brain MRI registration	0.814 ± 0.005	NR	NR	NA^*a*^	0.20 ± 0.04%	0.26
Yang X. et al. (2025) (TCDE-Net)	OASIS–3D brain MRI registration	0.800	NR	NR	SSIM: 0.979	0.356% (|*J*_Φ_| ≤ 0)	0.89
Zhao et al. (2023) (Patch-RegNet)	Head-and-neck CT–MR deformable registration (MR-Linac ART)	0.76	MSD: 1.9 mm	NR	NR	NR	5.6
Meng et al. (2025) (AutoFuse)	OASIS–inter-patient brain MRI registration	0.808	NR	NR	NR	0.025% NJD	0.38 (GPU)
Huang C. et al. (2025) (RS-MOCO)	T1Dataset210–cardiac T1 MRI motion-correction registration	0.827 ± 0.093	NR	HD_endo_: 6.19 px; HD_epi_: 6.71 px	NR	0.921 ± 0.552%	8.1 ± 0.6

NR, Not Reported in the original study. NA, Not applicable as a separately benchmarked value.

^*a*^NCC/MI was used only as the internal training loss, not reported as an evaluation result.

^*b*^NPJ restricted to the local tumor region rather than the standard whole-image formulation.

**Table 18:** Same-study non-transformer baseline comparisons in medical image registration.

Transformer-based or related model	Dataset/task	Reported model performance	Non-transformer baseline(s)	Reported baseline performance	Interpretation for fair comparison
SCT-Net (Liu X. et al., 2025)	EBRT–ICBT pelvic CT deformable registration in 80 cervical cancer patients	Bladder DSC = 0.76; Rectum DSC = 0.54; Bladder HD95 = 4.4 mm; Rectum HD95 = 5.9 mm	VoxelMorph; no-registration baseline	VoxelMorph: Bladder DSC = 0.70; Rectum DSC = 0.49; Bladder HD95 = 5.2 mm; Rectum HD95 = 6.3 mm. No registration: Bladder DSC = 0.65; Rectum DSC = 0.43	The same-study comparison provides clinically specific evidence for deformable registration in pelvic radiotherapy planning. The improvement over both VoxelMorph and the no-registration baseline suggests that the self-cross transformer design can enhance anatomical alignment in this task, while the interpretation should remain confined to the EBRT–ICBT CT setting and the reported pelvic organs.
Patch-RegNet/ViT-Morph (Zhao et al., 2023)	Head-and-neck CT–MR and MR–MR deformable registration	CT–MR: +6% DSC over VoxelMorph. MR–MR: +4% DSC over VoxelMorph	VoxelMorph and traditional registration methods in Monaco TPS	Relative VoxelMorph improvements: +6% for CT–MR; +4% for MR–MR	The reported improvements over VoxelMorph and traditional registration methods indicate the potential benefit of combining global transformer-based representation learning with hierarchical registration stages. Because the framework includes both alignment and deformable components, its results should be interpreted within the corresponding multi-stage head-and-neck registration workflow.
TransMorph (Chen et al., 2022)	3D brain MRI and phantom-to-CT registration	Substantial improvement over baseline methods reported	Existing registration methods and CNN-based baselines	–	The reported comparison situates TransMorph within established learning-based and conventional registration baselines for volumetric medical image registration. Its results are most appropriately interpreted within deformable or diffeomorphic registration settings that use comparable datasets, transformation models, and evaluation metrics.
TransMatch (Chen Z. et al., 2024)	LPBA40, IXI, and OASIS deformable brain MRI registration	DSC = 0.697 on LPBA40; 0.761 on IXI; 0.794 on OASIS; folding rate about 0.1%	VoxelMorph, CycleMorph, ViT-V-Net, and TransMorph	–	The comparison is informative for deformable brain MRI registration because TransMatch is evaluated against both CNN-based and transformer-based registration baselines. The reported Dice scores and low folding rate should be interpreted jointly, as registration quality depends not only on anatomical overlap but also on deformation-field plausibility and topology preservation.
XMorpher (Shi et al., 2022)	General deformable medical image registration	+2.8% DSC over VoxelMorph	VoxelMorph	VoxelMorph value –; relative gain +2.8% DSC	The relative improvement over VoxelMorph provides task-relevant evidence that cross-attention mechanisms may improve correspondence learning in deformable registration. This comparison should be interpreted within the deformable-registration family, since its Dice-based improvement is not directly comparable with rigid or affine alignment metrics.
SymTrans (Ma et al., 2022)	LPBA40, IXI, and OASIS unsupervised deformable registration	OASIS DSC approximately 0.83 reported in the qualitative benchmark; Table 12 reports OASIS DSC = 75.70%	VoxelMorph and CNN-based registration methods	–	SymTrans provides a relevant comparison with CNN-based unsupervised registration methods by emphasizing symmetric transformer-based modeling of long-range cross-image dependencies. Its reported performance should be interpreted in the context of dense deformable registration and should not be directly compared with rigid or affine registration results.
H-SGANet (Zhou and Cao, 2025)	OASIS and LPBA40 volumetric brain MRI registration	OASIS DSC improvement = +3.5%; LPBA40 DSC improvement = +1.5%	VoxelMorph with similar parameter count	VoxelMorph values –; relative gains +3.5% and +1.5%	The comparison with VoxelMorph under a similar parameter scale provides a useful basis for assessing architectural efficiency as well as registration accuracy. The reported improvements suggest that graph-attention and transformer-enhanced modeling may improve volumetric brain MRI registration when evaluated within comparable deformation settings.
SparseMorph (Bai et al., 2024)	IXI, OASIS, and MMWHS mono-/multi-modal deformable registration	Compared with TransMatch: +3.2% DSC on IXI; +2.9% DSC on OASIS; +9.7% and +11.4% DSC on MMWHS MRI–CT/CT–MRI	Primarily compared with TransMatch; also contextualized against deformable baselines	–	The reported gains indicate that sparse attention can improve deformable registration accuracy while addressing computational efficiency. Because the primary quantitative comparison is against another transformer-based model, SparseMorph is best interpreted as evidence for the benefit of efficient attention design rather than as a direct transformer-versus-CNN comparison.
RDP-Net (Wang H. et al., 2024)	LPBA40, Mindboggle, and IXI deformable brain MRI registration	Highest Dice and lowest HD/ASSD reported across datasets; 33% lower inference time reported	VoxelMorph, SyN, TransMorph, PCNet, RDN, and other registration baselines	–	RDP-Net is an important non-transformer reference because it demonstrates that well-designed CNN-based and diffeomorphic registration frameworks can remain highly competitive against transformer-based methods. Its inclusion helps contextualize transformer performance against strong deformable-registration baselines rather than weak or outdated comparators.
NIR (Sun et al., 2024)	Mindboggle101 and OASIS neural-field registration	OASIS surface DSC@1 mm = 0.9071 ± 0.034; near-zero non-positive Jacobian reported	SyN and CNN-based learning methods	–	NIR provides a non-transformer deformable registration comparator that emphasizes neural-field optimization, surface alignment, and topology preservation. Its performance is particularly relevant for interpreting whether transformer-based methods offer advantages over alternative continuous deformation models under cross-dataset and topology-sensitive evaluation.
uniGradICON (Tian et al., 2024)	Twelve public datasets; zero-shot and fine-tuning registration	L2R-Abdomen: average Dice = 78.9% with IO	SyN, VoxelMorph, LapIRN, and dedicated registration methods	–	uniGradICON provides a broad non-transformer and foundation-style reference for evaluating generalization across multiple anatomies, modalities, and registration benchmarks. Its zero-shot and fine-tuning settings make it particularly useful for assessing whether task-specific transformer models generalize beyond single-dataset registration scenarios.

Registration comparisons are valid only within the same transformation family. Rigid, affine, deformable, and diffeomorphic methods should not be ranked together using the same metric. VoxelMorph, SyN, ANTs-type methods, and CNN-based deformable networks are retained as essential non-transformer baselines for interpreting transformer-based registration claims.

As shown in Table 11 through Table 18, the available evidence does not support an absolute superiority of transformers over nontransformer methods; rather, it suggests that the advantage of these models depends on the type of task, baseline strength, assessment protocol, and data conditions.

## 6 Analysis and discussion of transformer-based models in medical image analysis

The rapid adoption of Transformer-based architectures in medical image analysis has yielded a diverse body of research spanning multiple tasks and imaging modalities. Analysis of these studies reveals consistent advantages associated with self-attention mechanisms, including the ability to capture long-range spatial dependencies, integrate multi-scale contextual information, and improve feature representation quality. At the same time, the analysis highlights critical limitations related to data efficiency, model interpretability, and computational cost, which continue to shape the trajectory of research in this area. The discussion that follows synthesizes key findings across existing studies, outlining methodological trends, performance observations, and emerging directions for Transformer-driven medical imaging.

### 6.1 Critical synthesis of architectural trends and remaining challenges

Beyond reporting performance metrics, this review shows that the success of transformer-based methods in medical imaging depends largely on the type of architectural design, the nature of the task, the size and variety of the data, and the evaluation protocol. A recurring pattern in the reviewed studies is that hybrid architectures, which combine the local feature extraction capabilities of CNNs with the ability of transformers to model long-range dependencies, show more consistent performance than purely pure transformers on many tasks. This is particularly important in medical image segmentation and registration, as these tasks require both local detail of boundaries and textures and rely on understanding the large-scale anatomical context.

Among the architectural choices, a few trends have been repeated more than others across tasks. First, multiscale and U-shaped encoder–decoder designs continue to be very effective for medical image segmentation and registration, as they allow for the integration of low-level, boundary, and spatial information with high-level representations. Second, hierarchical, windowed, or local-global attention, as seen in the Swin family and similar models, provides a practical solution to reduce the computational cost of full attention while preserving a large spatial context. Third, cross-attention and feature matching are of particular importance in image pair tasks, such as registration and reconstruction, because these tasks directly depend on learning the relationship between two images, two modalities, or an incomplete image and a reference image. Fourth, pre-training strategies, foundational models, and self-supervised learning have gradually become key factors for improving generalizability, especially in situations where medical annotation is limited, expensive, or heterogeneous.

Despite these trends, the evidence for the superiority of transformers over CNNs should be interpreted with caution. Many of the reviewed studies show that transformer-based models can outperform CNN baselines; however, these comparisons are not always conducted under completely fair conditions. Differences in dataset, data split, model size, pre-training, augmentation, number of epochs, hyperparameter settings, and even the strength of the selected baselines can affect the outcome of the comparison. Thus, the evidence does not support an absolute and general superiority of transformers over CNNs; rather, it suggests that transformers have a significant advantage in tasks that require global context, long-range dependencies, multimodal integration, or modeling of volumetric structures. In contrast, CNNs remain highly competitive baselines in tasks with limited data, lower computational demands, dominant local contexts, and applications requiring fast inference.

From a task perspective, none of the major areas of medical imaging can be considered completely solved. In classification, challenges such as robustness against domain shift, class imbalance, imaging center bias, and clinical interpretability remain. In segmentation, although high Dice scores have been reported in some datasets, segmentation of small structures, unclear boundaries, rare lesions, low-quality images, and multicenter data is still challenging. In reconstruction, improving metrics such as PSNR or SSIM does not necessarily mean full preservation of diagnostic details, and the risk of hallucination or removal of fine structures must be independently assessed. In registration, especially in deformable registration, simultaneously achieving high overlap, smooth deformation field, topology preservation, and anatomical validity remains an open issue.

Therefore, the main message of this review is not that transformers have replaced CNNs in general, but rather that the most successful methods typically employ a clever combination of inductive convolutional biases, global context modeling, multiscale design, appropriate regularization, and contextual evaluation. Future progress in this field will likely come not from simply increasing model size, but rather from designing fair comparisons, fully reporting computational costs, publishing code and model weights, external validation, multicenter evaluation, calibration, uncertainty analysis, and testing the impact of the model on clinical decision making.

### 6.2 Statistical scope of descriptive quantitative synthesis figures

**Figure 9:**
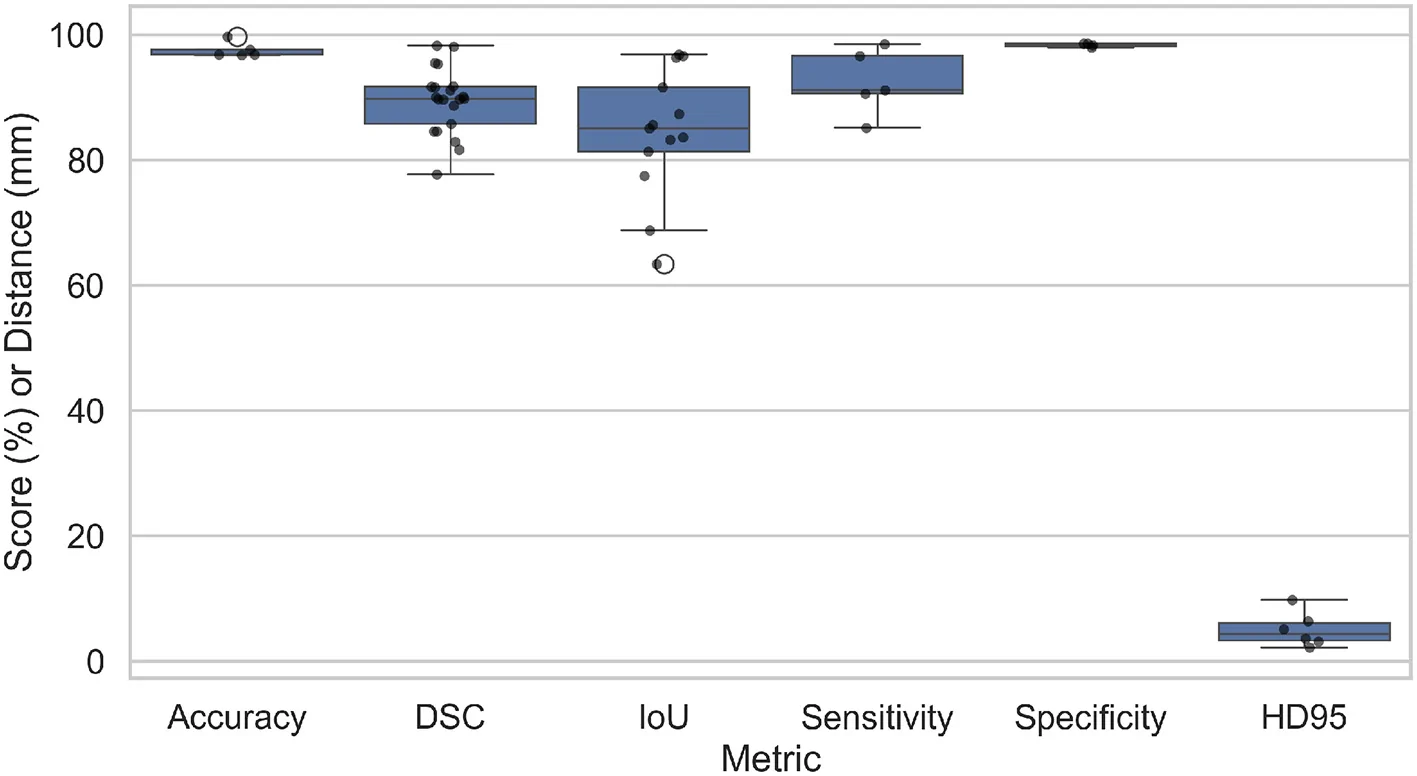
Distribution of performance metrics reported across transformer-based medical image segmentation studies. Boxplots summarize the variability of Dice score, IoU, sensitivity, specificity, and HD95, with individual study results overlaid.

**Figure 10:**
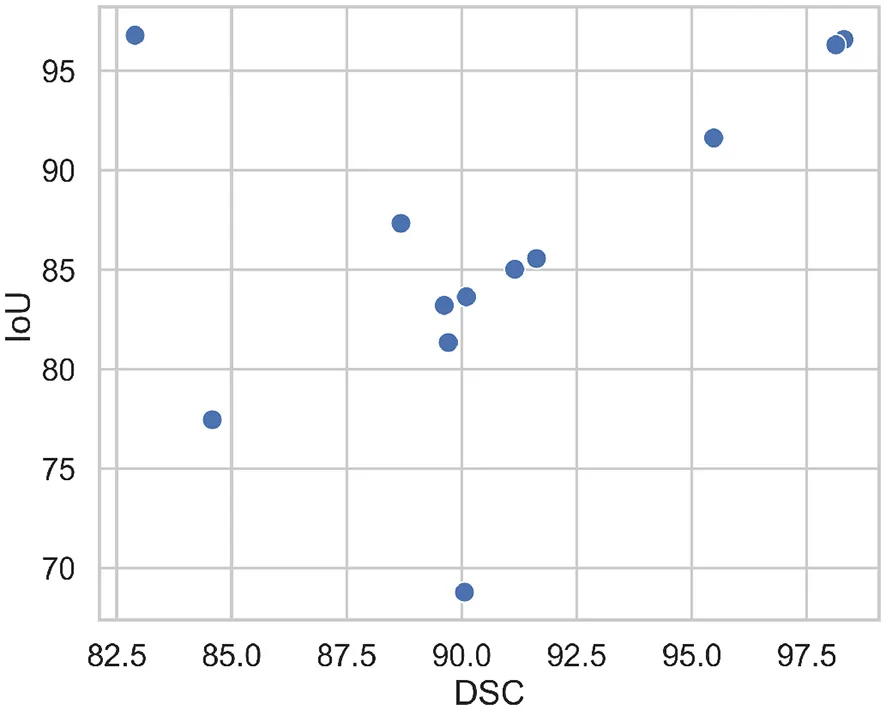
Relationship between Dice Similarity Coefficient (DSC) and Intersection over Union (IoU) across segmentation studies. Each point represents an individual study, illustrating the consistency between region overlap metrics.

**Figure 11:**
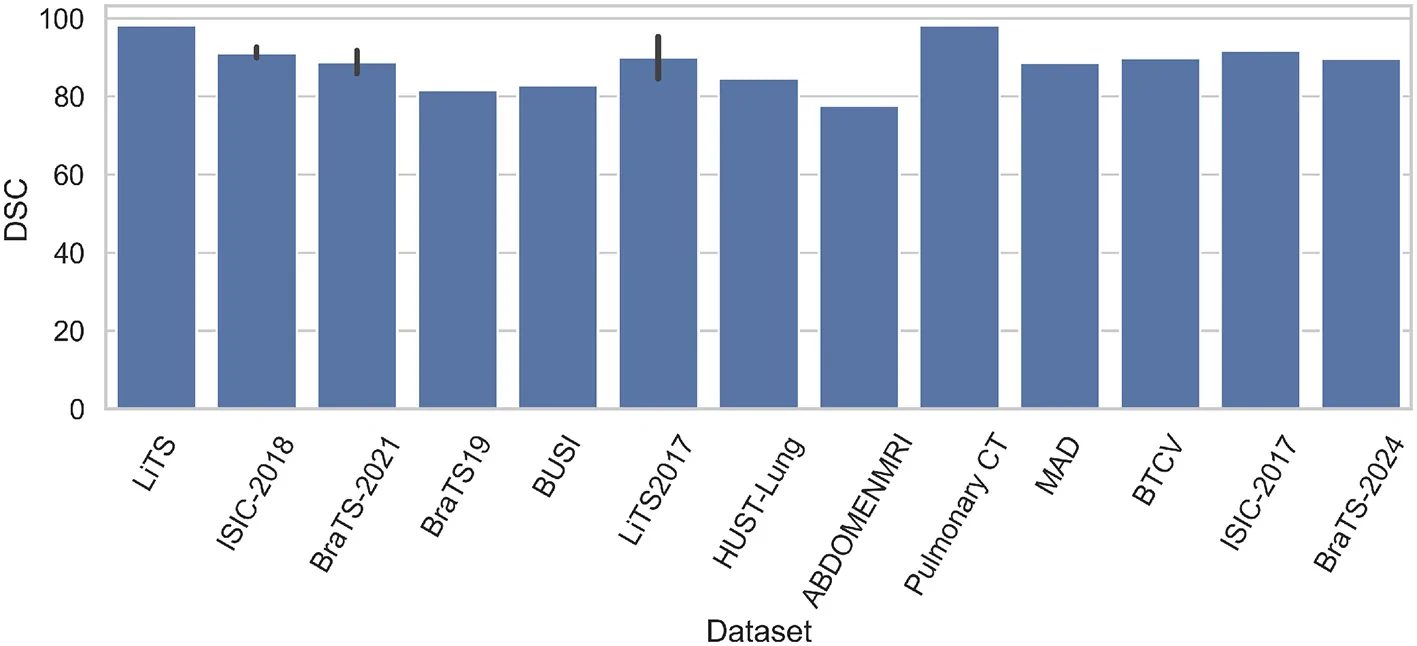
Comparison of Dice scores across validation datasets. The figure highlights variability in segmentation performance depending on dataset characteristics and imaging modalities.

**Figure 12:**
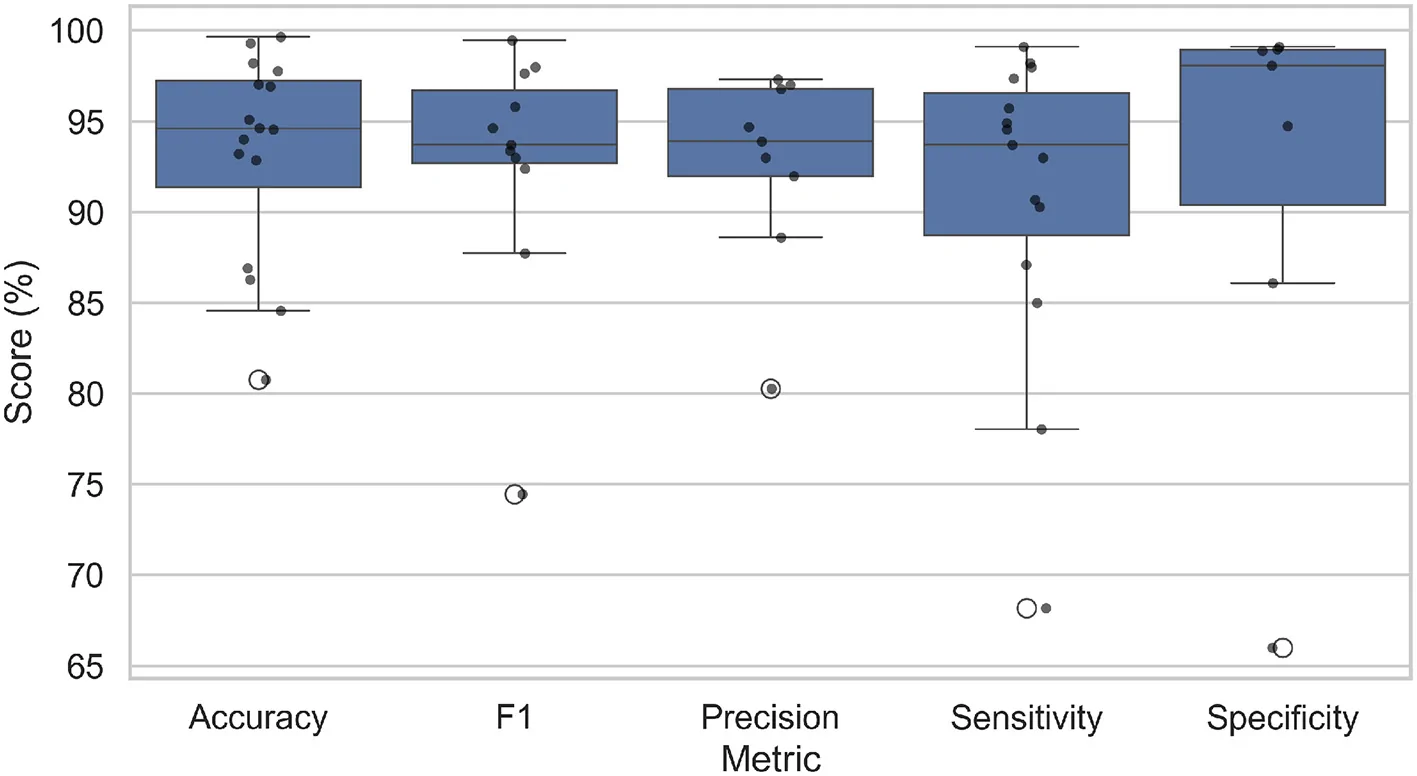
Distribution of performance metrics reported across transformer-based medical image classification studies. Boxplots summarize the central tendency and variability of accuracy, F1-score, precision, sensitivity, and specificity, with overlaid points indicating individual study results.

**Figure 13:**
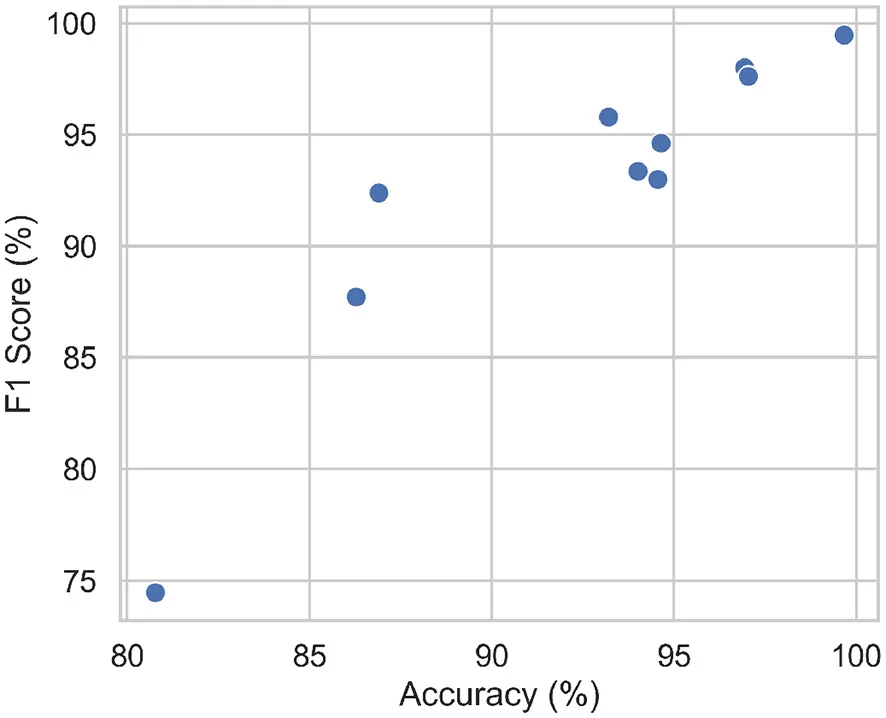
Relationship between accuracy and F1-score in transformer-based medical image classification studies. Each point represents an individual study, illustrating the trade-off between overall classification accuracy and the balance between precision and recall.

**Figure 14:**
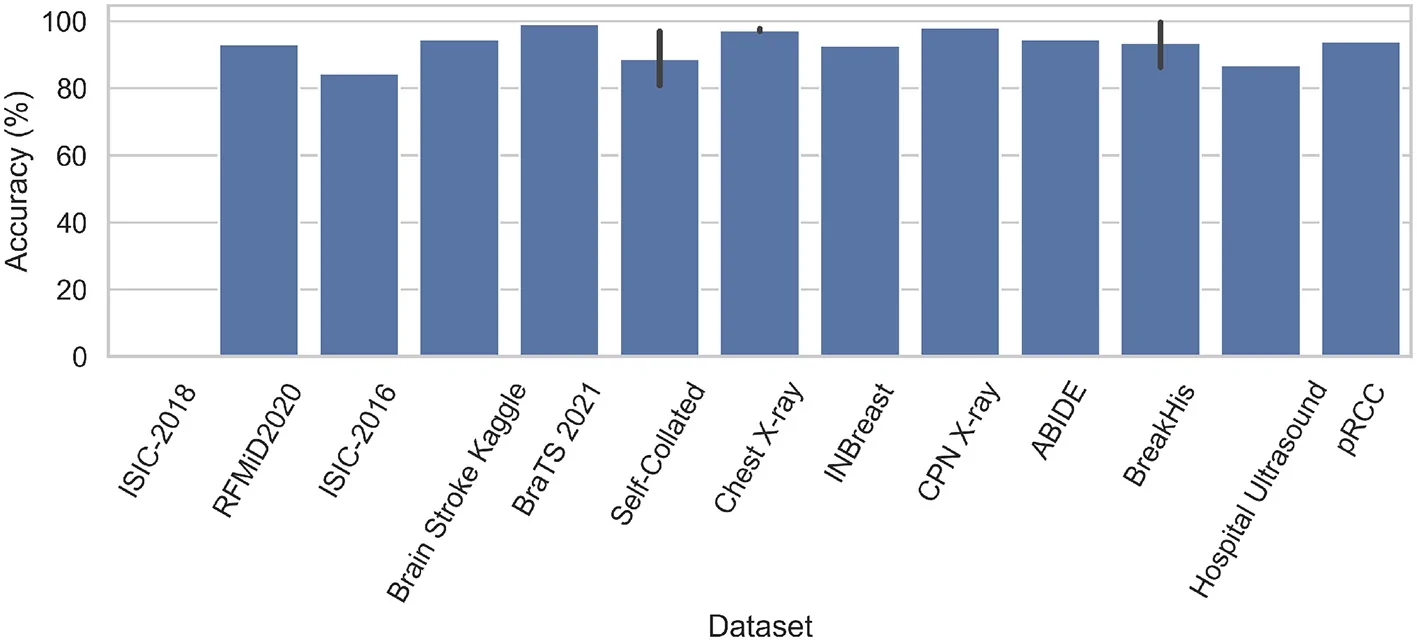
Comparison of classification accuracy across different validation datasets. The figure highlights variability in model performance across datasets, reflecting differences in task complexity, data quality, and class distribution.

**Figure 15:**
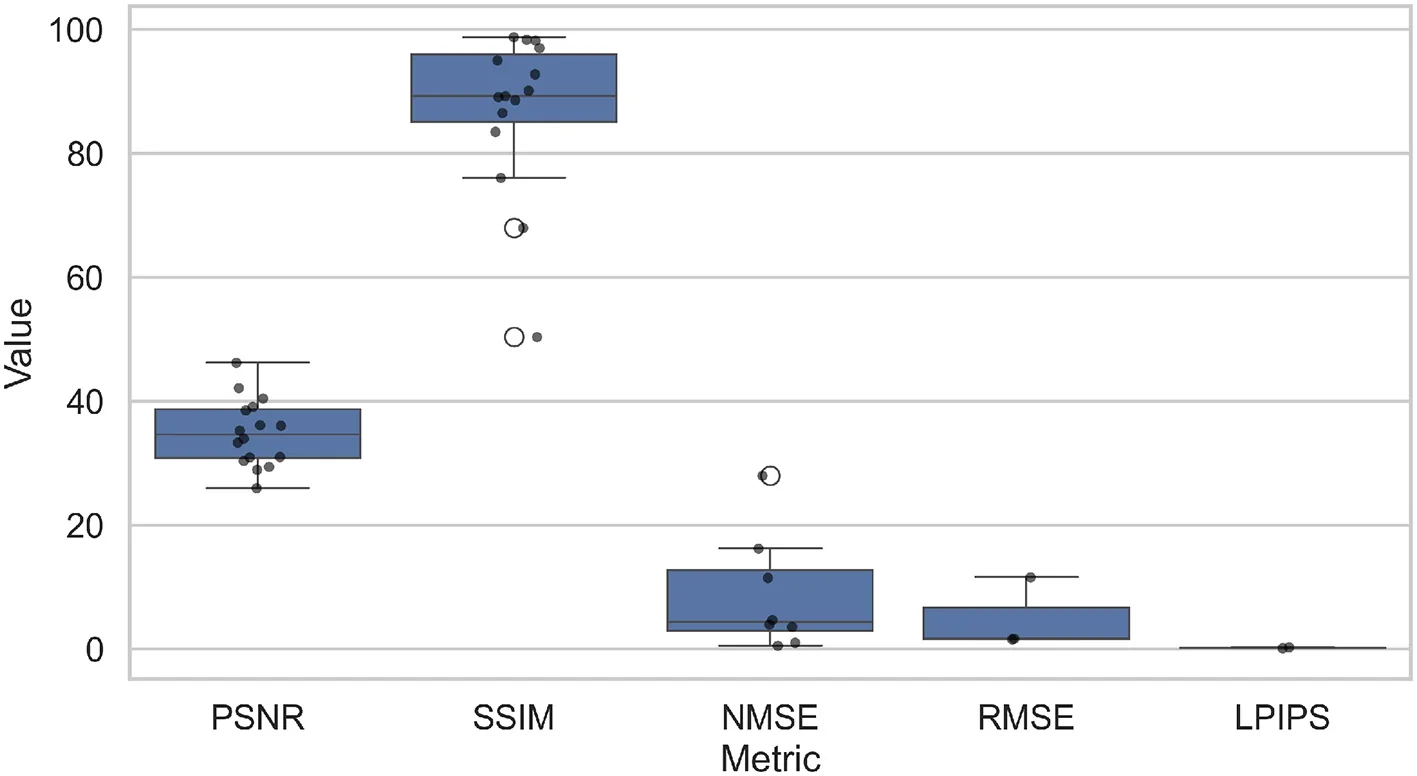
Distribution of performance metrics across transformer-based medical image reconstruction studies. Boxplots summarize variability in PSNR, SSIM, NMSE, RMSE, and LPIPS, with individual study values overlaid.

**Figure 16:**
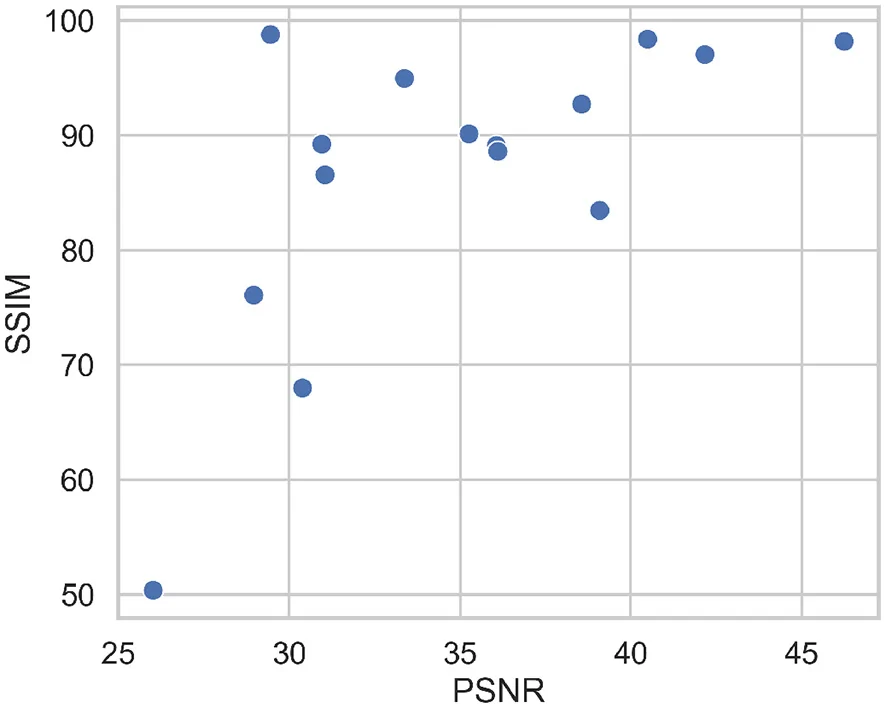
Relationship between PSNR and SSIM across reconstruction studies. Each point represents an individual study, illustrating the association between signal fidelity and structural similarity.

**Figure 17:**
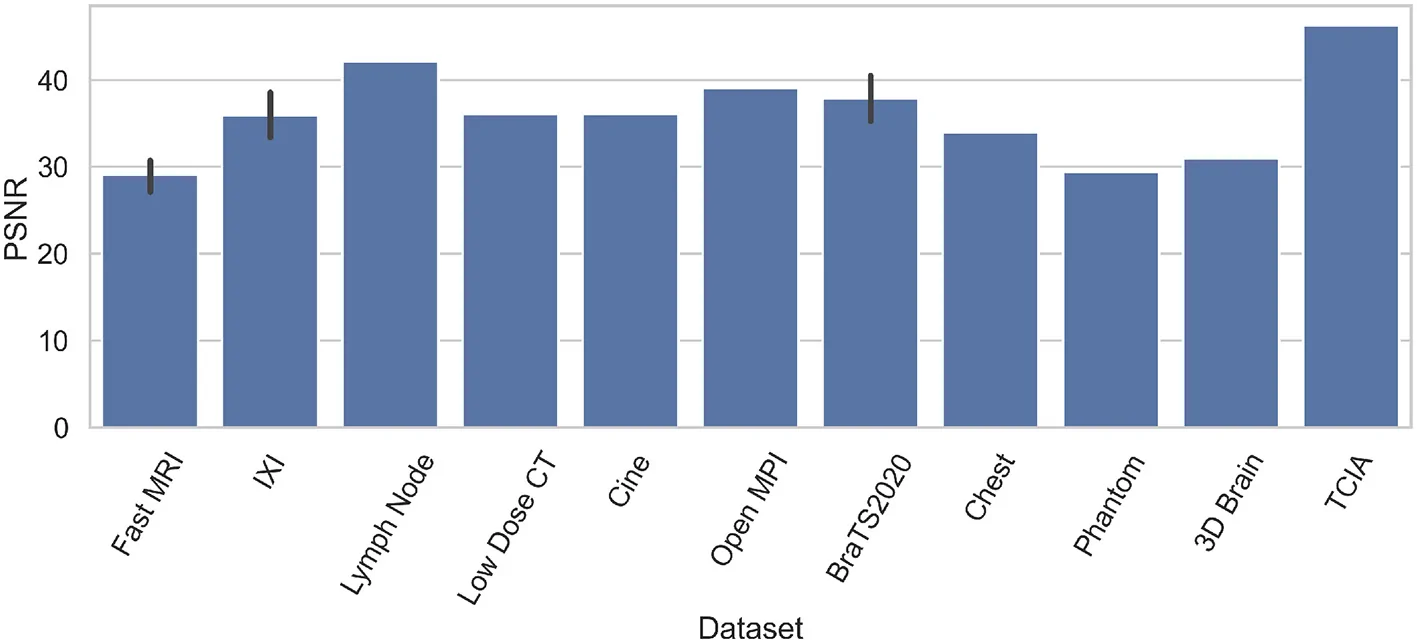
Comparison of PSNR values across different datasets. The variation highlights the influence of dataset characteristics and reconstruction difficulty on model performance.

**Figure 18:**
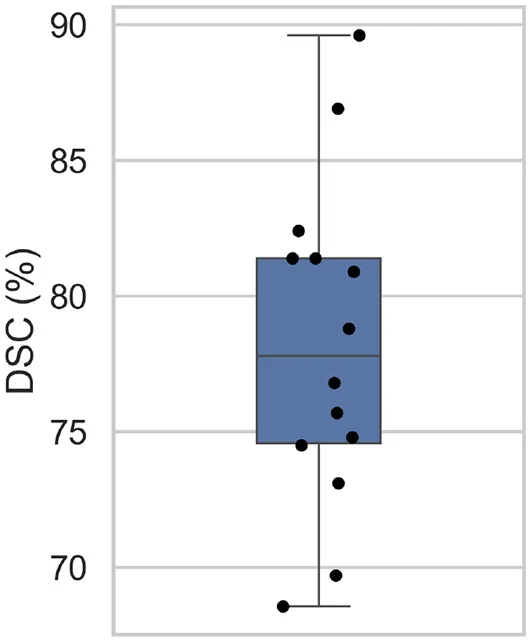
Distribution of Dice Similarity Coefficient (DSC) values across transformer-based medical image registration studies. The spread of values highlights variability in overlap performance, with most studies achieving DSC values between approximately 74% and 82%, and a smaller number reaching higher performance levels.

**Figure 19:**
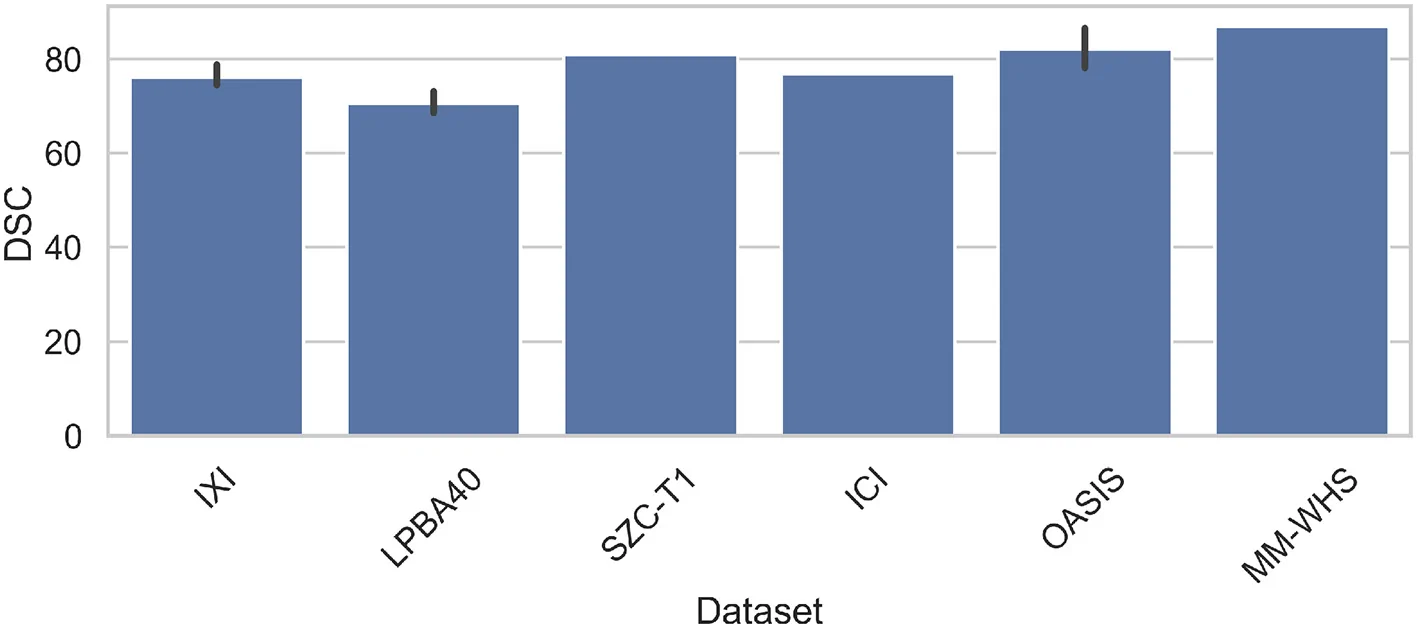
Comparison of DSC values across different validation datasets. Higher DSC values are more frequently observed for the OASIS dataset, while greater variability and lower performance are evident for datasets such as LPBA40, reflecting differences in registration complexity.

**Figure 20:**
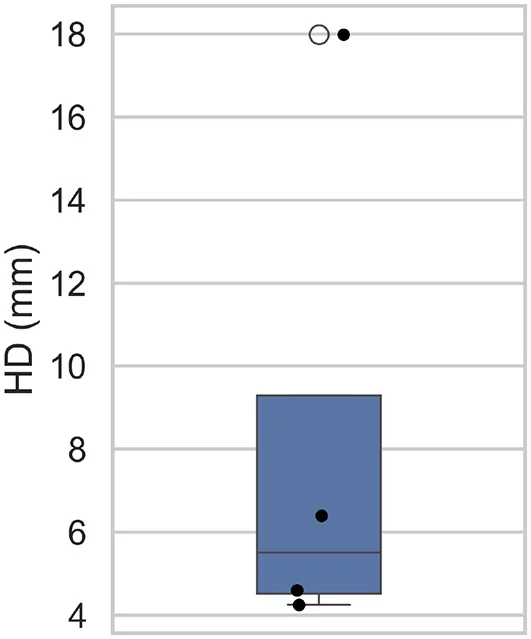
Distribution of Hausdorff Distance (HD) values reported in registration studies. The wide range of values indicates variability in boundary alignment accuracy, with lower values corresponding to more precise anatomical alignment.

**Figure 21:**
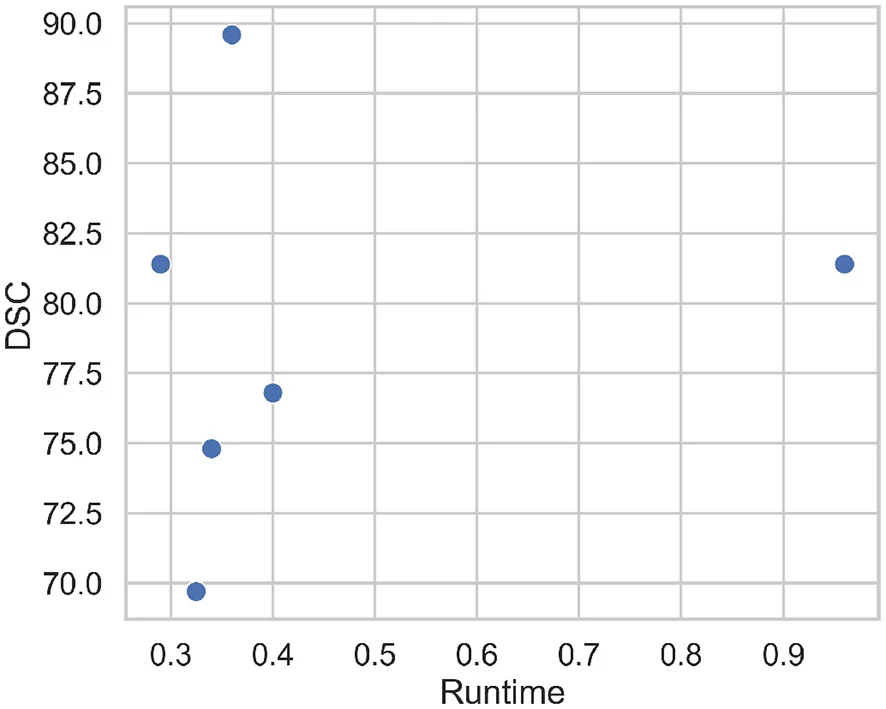
Relationship between DSC and runtime across registration studies. The absence of a clear trend suggests that higher registration accuracy is not necessarily associated with increased computational cost, indicating that efficient and accurate models can be achieved.

The figures presented in Section 6 are intended to be descriptive representations of the measures reported in the reviewed studies and should not be interpreted as formal statistical meta-analyses. The studies included in this review are heterogeneous in terms of task type, imaging modality, dataset, problem difficulty level, training and test data partitioning, validation strategy, baselines used, and assessment criteria. In addition, many studies did not report the information necessary to conduct a formal meta-analysis, such as pooled effect sizes, standard errors, confidence intervals, variances, exact sample sizes at the level of each metric, or sufficient information for statistical weighting. Therefore, Figures 9–21 are presented as “descriptive quantitative synthesis” and “benchmarking based on reported measures,” rather than as pooled estimates of effect or statistical tests of superiority of one model family over another.

In the boxplots, each point represents a reported value from a study or a specific combination of study, model, dataset, and metric. For clarity, the number of non-missing values contributing to the plot of each metric is reported in the figure caption. However, these points should not necessarily be interpreted as completely independent statistical observations, as some values may have been derived from the same paper, research group, model, or multiple related datasets. In cases where a study reported multiple results for multiple datasets or evaluation scenarios, these values were used to represent the reported range of performance, but were not used to perform inferential testing or draw conclusions about statistical superiority.

Due to the lack of consistent reporting of variance, standard error, confidence intervals, sample size, and risk of bias across most studies, we did not perform a publication bias test or construct a funnel plot in this review. Performing such tests in the absence of comparable effect sizes and valid criterion errors can be misleading, especially when studies have used different tasks, datasets, and assessment protocols. Therefore, rather than providing a test of publication bias, limitations due to study heterogeneity, incomplete reporting uncertainty, and the possibility of dependence among multiple outcomes are explicitly stated in the Discussion text.

Accordingly, the descriptive quantitative synthesis figures should be interpreted as tools for identifying general trends, dispersion of reported measures, reporting gaps, and limitations of comparability. They are not designed to directly rank models or to draw definitive conclusions about the statistical superiority of transformers over other architectures. Stronger conclusions about the superiority of one method are possible only when models have been tested on the same dataset, similar split, identical criteria, fair baselines, and comparable assessment protocols.

### 6.3 Cautious interpretation of dataset-based analyses

Analysis based on dataset names should be interpreted with caution, as a single dataset may have been used in different studies with different definitions of task, label, annotation level, data split, and evaluation protocol. For example, a skin dataset may be used in one study for binary lesion classification, in another study for multiclass classification, and in another study for 2D lesion segmentation. In such situations, aggregating results under a general dataset label can mask important differences such as task type, difficulty level, class imbalance, dimensionality, annotation quality, and differences in evaluation criteria.

Accordingly, the figures for dataset-specific performance in this review are intended as descriptive representations of the values reported in the original studies, not as direct comparisons or definitive rankings of datasets or models. Accuracy, Dice score, or other metrics can only be interpreted within the context of the same task, modality, annotation type, data split, comparative baselines, and validation protocol. Therefore, if a dataset such as ISIC-2018 has been used in multiple studies with different settings, the results of those studies should not be combined into a single group without distinguishing between the task and methodological context.

To reduce the risk of misinterpretation, dataset-specific analyses are presented in a contextualized manner. This means that the reported performance is interpreted not only by the dataset name, but also by the type of task, such as binary classification, multiclass classification, 2D segmentation, 3D segmentation, or analysis of different anatomical structures. This approach makes it easier to distinguish between seemingly similar but methodologically different datasets and avoids simplistic conclusions about the superiority of one dataset, model, or architectural family.

### 6.4 Task-specific performance patterns

#### 6.4.1 Segmentation

The quantitative evaluation of transformer-based models for medical image segmentation, summarized in Table 19, demonstrates strong and consistent performance across diverse datasets and imaging modalities. The DSC, the most widely reported metric, typically exceeds 85%, with several studies achieving values above 95%. Notably, Anwar et al. (2025) and Lv et al. (2025) report DSC values exceeding 98%, indicating highly accurate segmentation performance.

**Table 19:** Performance metrics of transformer-based models in medical image segmentation studies.

Study	Validation dataset	Accuracy (%)	DSC (%)	IoU (%)	Sensitivity (%)	Specificity (%)	HD95 (mm)
Anwar et al. (2025)	LiTS	99.70	98.30	96.60	98.50	NR	NR
Cai et al. (2025)	ISIC-2018	NR	91.62	85.58	NR	NR	NR
Chen J. et al. (2024)	BraTS-2021	NR	91.74	NR	NR	NR	NR
Chen T. et al. (2024)	BraTS-2021	NR	85.80	NR	NR	NR	3.14
Dai et al. (2024)	ISIC-2018	NR	90.10	83.66	NR	NR	NR
Guo et al. (2024)	ISIC-2018	96.75	91.15	85.03	91.13	97.96	NR
Hua et al. (2024)	BraTS19	NR	81.65	NR	NR	NR	5.08
Iqbal et al. (2025)	ISIC-2018	97.60	95.47	91.65	NR	98.55	NR
Li C. et al. (2025)	BUSI	NR	NR	63.38	NR	NR	NR
Li G. et al. (2025)	ISIC-2018	NR	90.06	68.80	NR	NR	NR
Liao et al. (2024)	LiTS2017	NR	84.58	77.48	NR	NR	NR
Liu H. et al. (2024)	HUST-Lung	NR	84.55	NR	85.11	NR	NR
Liu J. et al. (2025)	ABDOMENMRI	NR	77.67	NR	NR	NR	NR
Lv et al. (2025)	Pulmonary Chest CT	NR	98.13	96.33	NR	NR	6.34
Ou et al. (2024)	LiTS2017	NR	95.35	NR	96.61	NR	NR
Ruan et al. (2025)	ISIC-2018	NR	89.71	81.35	NR	NR	NR
Wang C. et al. (2024)	MAD	NR	88.67	87.36	NR	NR	NR
Wu J. et al. (2025)	BTCV	NR	89.80	NR	NR	NR	2.18
Wu R. et al. (2025)	ISIC-2017	96.80	91.72	NR	90.56	98.31	NR
Zhang et al. 2025b)	BUSI	96.80	82.90	74.7	NR	98.60	9.80
Zheng et al. (2025)	BraTS-2024	NR	89.70	NR	NR	NR	3.56
Zhu et al. (2024)	ISIC-2018	NR	89.62	83.23	NR	NR	NR

NR, Not Reported in the original study.

The distribution of segmentation metrics is illustrated in Figure 9. Dice scores and IoU values are generally concentrated at high levels, indicating strong overlap between predicted and ground-truth regions. However, some variability is observed, particularly in DSC values ranging from approximately 77% to over 98%, suggesting differences in task difficulty and dataset complexity. In contrast, HD95 values exhibit greater dispersion, indicating variability in boundary-level accuracy across studies.

The relationship between DSC and IoU, shown in Figure 10, reveals a strong positive correlation, as expected given that both metrics quantify region overlap. This consistency suggests that models achieving high Dice scores also tend to perform well on IoU, reinforcing the reliability of these metrics for evaluating segmentation quality.

Dataset-specific performance trends are presented in Figure 11. Considerable variation in Dice scores is observed across datasets, with certain benchmarks, such as LiTS and ISIC-2018, frequently associated with higher performance, whereas others, including ABDOMENMRI and BUSI, exhibit comparatively lower values. This variation reflects differences in anatomical complexity, imaging modality, and data quality. Importantly, it highlights the strong dependence of segmentation performance on the evaluation dataset, complicating direct comparisons between studies. In segmentation analysis, the Dice score reported for different datasets can only be interpreted in the context of the same task and annotation type. 2D skin lesion segmentation, brain tumor segmentation, abdominal multi-organ segmentation, and 3D volumetric structure segmentation are not identical in terms of difficulty, evaluation criteria, and clinical significance of the Dice score. Therefore, Figure 11 should not be interpreted as a direct ranking of datasets or segmentation models, but rather as a descriptive representation of the range of Dice values reported in different contexts.

Transformer-based segmentation models demonstrate high performance across a range of tasks, particularly in terms of region overlap metrics. However, variability across datasets and the limited reporting of complementary metrics, such as HD95, indicate that performance is not uniformly robust and that careful consideration is required when interpreting results across studies.

In medical image segmentation tasks, transformer-based models have received widespread attention due to their ability to model long-range spatial dependencies and integrate multiscale information. Unlike classification, segmentation requires accurate prediction at the pixel or voxel level, and therefore the model must simultaneously preserve two types of information: local details related to boundaries, textures, and small structures, and the overall context related to anatomical location and spatial relationships between organs or lesions. For this reason, in many studies, the most successful designs have used U-shaped, encoder–decoder, or CNN–Transformer hybrid architectures instead of using pure transformers.

The prevailing paradigm in segmentation studies is that CNNs are strong at preserving local and boundary details, while transformers can better model the larger spatial context and relationships between more distant regions of the image. This combination is particularly important in multi-organ segmentation, brain tumors, skin lesions, polyps, abdominal structures, 3D images, and multimodal data. In such tasks, local boundary recognition alone is not enough; the model must know in which anatomical region the target structure is located, what structures it borders, and how it relates to the overall image background.

Despite the high Dice scores reported in many studies, the interpretation of these results must be contextualized. A high Dice score for segmenting a large, well-defined structure, such as an organ with a well-defined border, is not directly comparable to a similar Dice score for segmenting a small, irregular, or low-contrast lesion. 2D and 3D segmentation also differ significantly in terms of difficulty, computational memory, annotation type, and prediction stability. In 3D segmentation, the model must maintain spatial continuity between slices, and small errors may have a larger effect on the final volume. Therefore, the reported performance for segmentation is only meaningful when the target structure type, data dimensions, annotation type, data split, and evaluation criteria are clearly specified.

Architecturally, several important directions in segmentation are visible. First, Swin Transformer and windowed attention-based models have shown that reducing attention cost through computational localization can be beneficial for high-resolution medical images. Second, hybrid U-Net and Transformer models are still very effective, as skip connections help preserve spatial details. Third, promptable and foundation models such as MedSAM represent a movement of the field toward more general models that can be used with prompts or adaptation for different segmentation tasks. Fourth, alternative architectures such as Mamba and state-space models have been proposed as a solution to reduce computational cost and model long-range dependencies in volumetric data.

However, medical segmentation is still far from solved. Small structures, rare lesions, fuzzy boundaries, noisy images, artifacts, discrepancies between expert annotations, and differences between imaging centers remain serious challenges. Furthermore, many studies only report Dice or IoU, while for clinical application, boundary measures such as Hausdorff distance, local error analysis, uncertainty, calibration, and assessment of the impact of error on clinical decision are also necessary. For example, a model may have a high Dice but be inaccurate in small, clinically important regions. Therefore, future progress in segmentation should move beyond a focus on Dice score alone and move toward assessing robustness, external validation, interpretability, computational cost, and real clinical impact.

#### 6.4.2 Classification

The quantitative analysis of transformer-based models for medical image classification, summarized in Table 20, indicates consistently strong performance across diverse datasets and clinical applications. Reported accuracy values are generally high, with most studies exceeding 90%. Notably, several works achieve near-perfect performance, such as Kari et al. (2026) and Yan et al. (2025), which report accuracies above 99%. Similarly, F1-scores, where available, are predominantly above 90%, indicating a strong balance between precision and recall.

**Table 20:** Performance metrics of transformer-based models in medical image classification studies.

Study	Validation dataset	Accuracy (%)	F1-score (%)	Precision (%)	Sensitivity (%)	Specificity (%)
Alqahtani et al. (2025)	ISIC-2018	NR	93.70	93.90	93.70	NR
Bi et al. (2023)	DR RFMiD2020	93.20	95.80	96.80	94.90	NR
Dahou et al. (2025)	ISIC-2016	84.56	NR	NR	90.30	66.00
Hossain et al. (2025)	Kaggle Brain Stroke Dataset	94.55	93.00	93.00	93.00	NR
Kari et al. (2026)	BraTS 2021	99.30	NR	NR	99.10	98.90
Khaliq et al. (2025)	Self-Collated	96.93	98.00	97.00	98.00	NR
Kumar et al. (2025)	Self-Collated	80.76	74.46	80.28	78.04	NR
Li J. et al. (2024)	Chest X-ray	97.76	NR	NR	95.73	98.97
Liu S. et al. (2024)	Chest X-ray	97.03	97.63	NR	NR	NR
Mohamed et al. (2024)	INBreast	92.86	NR	NR	68.18	98.08
Nejati Manzari et al. (2026)	CPN X-ray	98.20	NR	NR	98.20	99.10
Parvathy et al. (2025)	ABIDE	94.64	94.63	94.70	94.55	94.73
Song et al. (2024)	BreakHis	95.10	NR	NR	NR	NR
Sun et al. (2023)	Hospital ultrasound dataset	86.90	92.40	NR	87.10	86.10
Wang et al. (2026)	BreakHis	86.28	87.72	88.61	84.99	NR
Yan et al. (2025)	BreakHis	99.66	99.47	97.30	97.37	NR
Zhang et al. (2024)	pRCC	94.00	91.32	91.98	90.68	NR

NR, Not Reported in the original study.

The overall distribution of performance metrics is illustrated in Figure 12. Most metrics, including F1-score, precision, and sensitivity, are tightly clustered at high values, suggesting that transformer-based models consistently achieve strong classification performance. However, some variability is evident, particularly in sensitivity and specificity. For example, a notably lower sensitivity of 68.18% is reported in Mohamed et al. (2024), suggesting that certain models may struggle to correctly identify positive cases on more challenging datasets. This highlights that while overall performance is high, it is not uniformly robust across all evaluation scenarios.

The relationship between accuracy and F1-score, shown in Figure 13, demonstrates a generally strong positive correlation, indicating that models with high overall accuracy tend to also maintain balanced classification performance. However, some dispersion is present, suggesting that high accuracy does not always guarantee an optimal F1-score. This discrepancy may arise in class-imbalanced settings, where accuracy can remain high despite poorer performance on minority classes.

Dataset-specific performance trends are illustrated in Figure 14. Considerable variation in accuracy is observed across datasets, with certain datasets, such as BreakHis and BraTS 2021, exhibiting particularly high performance, whereas others, including ISIC-2016, show lower accuracy. This variation reflects differences in dataset characteristics, including image quality, class balance, and task complexity. Importantly, it suggests that model performance is highly dependent on the evaluation dataset, limiting the generalizability of results across different clinical contexts. In classification analysis, the variation in accuracy across datasets is only interpreted descriptively, as different datasets differ in terms of number of classes, level of imbalance, image quality, disease type, modality, and validation protocol. Therefore, higher or lower accuracy values in Figure 14 should not be interpreted as a direct comparison between datasets or models. The figure merely illustrates that the performance reported in classification studies is highly dependent on the data context, task definition, and evaluation conditions.

Transformer-based models demonstrate strong classification performance, with consistently high accuracy and F1 scores across a range of studies. This performance can be partly attributed to the ability of transformer architectures to capture both local and global contextual information. However, the observed variability across datasets and metrics indicates that performance is influenced by dataset characteristics and evaluation practices, necessitating cautious interpretation when comparing results across studies. Furthermore, the lack of variance reporting and the heterogeneity of evaluation protocols limit the robustness of quantitative comparisons. Despite these limitations, the consistently high performance reported provides strong evidence of the potential of transformer-based models to advance automated medical image classification across diverse clinical applications.

In medical image classification tasks, the general pattern of studies suggests that transformer-based models are most advantageous when the problem requires understanding broad contextual relationships, sparse patterns in the image, or distinguishing between subtle disease features. Unlike CNNs, which rely primarily on incremental extraction of local features, transformers can model the relationship between distant regions in the image. This feature is particularly important in the classification of pathology images, retina, skin lesions, chest images, MRI, and multi-disease images, because diagnostic features are not always located in a limited area and sometimes the spatial distribution of several small patterns throughout the image determines the final label.

However, the reviewed evidence suggests that the success of transformers in classification is not absolute and independent of the data conditions. In many studies, CNN-Transformer hybrid models have shown more consistent performance than pure transformers, as CNNs still have advantages in extracting edges, textures, local patterns, and small image structures. This is particularly important in medical images, which often contain limited data, noise, class imbalance, and subtle differences between classes. Therefore, in classification, the main advantage of transformers usually appears when the global context modeling capability is combined with the local inductive biases of CNNs.

In terms of performance, accuracy, sensitivity, specificity, precision, and F1-score have been widely reported in various studies, but these metrics should be interpreted with caution. High accuracy on a balanced, multiclass dataset is not the same as high accuracy on an unbalanced or binary dataset. Also, high performance on general datasets or small benchmarks does not necessarily indicate the readiness of a model for clinical use. For example, in some studies, the model has been evaluated on data with uniform image quality and limited split, while in the real environment, images may come from different devices, different centers, different populations, and heterogeneous imaging protocols.

From a comparison perspective with CNNs, the available evidence suggests that transformers can report better performance in some settings, but this conclusion is only defensible when the comparison is made under fair conditions. Differences in model size, use of pretraining, number of epochs, augmentation, optimization method, data split, and baseline strength can significantly change the comparison result. Therefore, the classification discussion should not lead to the general conclusion that transformers are better than CNNs in all conditions; Rather, the more precise conclusion is that transformers have a potential advantage in classifications that require global context, long-range dependencies, and multilevel feature combinations, while CNNs remain competitive in small data, local tasks, and low-cost applications.

Unsolved issues in medical classification are also significant. First, robustness against domain shift is still a major challenge; a model that performs well on one site or device type may show significant degradation on data from another site. Second, class imbalance, especially in rare diseases, can cause general measures such as image accuracy to not reflect the true performance. Third, clinical interpretability is still insufficient, because the clinician needs to know which image regions and which image patterns contributed to the model's decision. Fourth, many studies still do not report calibration, uncertainty estimation, and prospective evaluation. Therefore, although classification is one of the most mature areas of transformer application in medical imaging, it is still not fully resolved in terms of generalizability, clinical reliability, and fair comparison with robust baselines.

#### 6.4.3 Reconstruction

The quantitative evaluation of transformer-based models for medical image reconstruction, as summarized in Table 21, demonstrates strong performance across a range of datasets and imaging modalities. Peak signal-to-noise ratio (PSNR), the most commonly reported metric, varies considerably across studies, ranging from approximately 26 dB to over 46 dB. Notably, Yuan et al. (2025) reports the highest PSNR of 46.25 dB, indicating superior reconstruction fidelity, while lower values are generally associated with more challenging datasets or reconstruction settings.

**Table 21:** Performance metrics of transformer-based models in medical image reconstruction studies.

Study	Dataset	PSNR (dB)	SSIM (%)	NMSE (%)	RMSE (HU)	VIF (%)	LPIPS
Chen et al. (2025)	Fast MRI	26.01	50.38	NR	NR	NR	NR
Han and Du (2025)	Fast MRI	28.96	76.10	27.95	NR	NR	NR
Hou et al. (2025)	IXI	38.56	92.76	4.66	NR	NR	NR
Huang J. et al. (2025)	Fast MRI	30.38	68.00	NR	NR	NR	0.259
Li Y. et al. (2025)	Lymph Node	42.16	97.05	NR	NR	69.01	NR
Lin et al. (2024)	Low Dose CT Image	36.06	89.14	NR	1.63	NR	NR
Lyu et al. (2023)	Cine	36.10	88.61	NR	1.57	NR	NR
M.khair et al. (2026)	Open MPI	39.08	83.50	16.20	NR	NR	NR
Tu et al. (2025)	BraTS2020	35.25	90.17	NR	NR	NR	0.109
Wang et al. (2023)	IXI	33.37	95.00	1.00	NR	NR	NR
Wang W. et al. (2025)	Chest	33.98	NR	NR	NR	NR	NR
Wang Y. et al. (2024)	Phantom	29.45	98.80	4.00	NR	NR	NR
Yang T. et al. (2025)	3D Brain	30.95	89.27	3.56	NR	NR	NR
Yuan et al. (2025)	TCIA	46.25	98.20	NR	11.56	NR	NR
Zhang et al. 2025a)	Fast MRI	31.04	86.58	11.50	NR	NR	NR
Zou et al. (2025)	BraTS2020	40.49	98.40	0.51	NR	NR	NR

NR, Not Reported in the original study.

The distribution of reconstruction metrics is illustrated in Figure 15. PSNR and SSIM values are generally concentrated at relatively high levels, indicating that transformer-based models effectively preserve both signal fidelity and structural similarity. However, metrics such as NMSE exhibit greater variability, with values ranging from below 1% to over 25%, reflecting differences in reconstruction difficulty and noise levels across datasets.

The relationship between PSNR and SSIM, shown in Figure 16, reveals a strong positive association, indicating that improvements in signal fidelity are typically accompanied by better structural preservation. This consistency suggests that transformer-based models can jointly optimize multiple aspects of reconstruction quality. Nevertheless, some dispersion is observed, highlighting that high PSNR does not always guarantee optimal perceptual similarity.

Dataset-specific performance trends are presented in Figure 17. Considerable variation in PSNR is observed across datasets, with higher values typically reported for TCIA and BraTS2020, whereas lower values are associated with Fast MRI datasets. This variation reflects differences in imaging modality, acquisition protocols, and reconstruction complexity. As with other tasks, this indicates that performance is highly dependent on the evaluation dataset, limiting direct comparability across studies.

Transformer-based models demonstrate strong capability in medical image reconstruction, achieving high fidelity and structural similarity across diverse tasks. However, variability across datasets and reconstruction conditions suggests that performance remains context-dependent, and careful consideration is required when interpreting results across studies.

In medical image reconstruction, the role of transformers is different from segmentation and classification. The main goal in reconstruction is usually to produce or recover a higher quality image from incomplete, noisy, undersampled, or artifact-prone data. This task is of great importance in MRI, CT, CBCT, PET, super-resolution, denoising, artifact reduction and image-to-image translation. Here, the model must not only improve the appearance of the image, but also preserve the true diagnostic information; therefore, improving metrics such as PSNR and SSIM alone is not enough to prove clinical quality.

Transformers have an important advantage in reconstruction because they can model long-range dependencies, recurring structures, large spatial relationships, and connections between different image regions. In MRI reconstruction, for example, the information lost in undersampled regions may be related to larger spatial patterns. In CT or CBCT artifact reduction, artifacts can also be distributed throughout the image and their removal requires an understanding of the relationship between distant structures. Therefore, global context modeling can help reconstruct more consistent structures and reduce artifacts.

However, reconstruction is one area where the risk of misinterpreting numerical performance is very high. PSNR and SSIM metrics usually measure the overall similarity of the reconstructed image to the reference image, but do not necessarily guarantee the preservation of small diagnostic details, fine boundaries, small lesions, or important textural information. A model may have a high PSNR but oversmooth small structures, or in certain situations produce unrealistic details. This is especially important in generative, diffusion-based, or GAN-based models, because better visual appearance does not always mean higher diagnostic accuracy.

From an architectural perspective, reconstruction studies show that the combination of the image domain and the frequency domain or k-space can be very important. Models that work only in the image domain may ignore some physical limitations of acquisition, while models that incorporate frequency-domain information, physical constraints, or data consistency are more reliable in terms of reconstruction. Hybrid CNN-Transformer architectures are also important here, as CNNs are effective in reconstructing local details and texture, and transformers can better preserve global consistency and long-range dependencies.

Compared to CNNs, evidence shows that transformers and hybrid models can perform better in terms of PSNR, SSIM, or artifact reduction in some reconstruction scenarios. However, this comparison is only reliable when the acceleration factor, undersampling type, modality, noise level, data size, preprocessing, loss function, and evaluation protocol are the same. Otherwise, the numerical differences between the models may be due to differences in the problem conditions rather than the architecture.

Unresolved issues in reconstruction include maintaining diagnostic fidelity, avoiding hallucination, reader-based evaluation, testing on external data, and measuring the impact of reconstruction on the final diagnosis. Many studies have not yet demonstrated how the reconstructed image affects physician performance, small lesion detection, quantitative measurement, or treatment decision-making. In addition, the computational cost of bulky reconstruction models can hinder their practical use in clinical settings, especially in applications where reconstruction speed is important for workflow. Therefore, the future of reconstruction should focus on combining numerical performance, physical fidelity, clinical evaluation, and computational efficiency.

#### 6.4.4 Registration

Performance metrics for transformer-based medical image registration studies are summarized in Table 22, with corresponding visualizations illustrating metric distributions and relationships across studies. The reported DSC values range from 68.57% (Liu et al., 2026) to 89.60% (Li W. et al., 2024), with most studies achieving values between approximately 74% and 82%. This indicates generally strong overlap performance, although with noticeable variability across datasets and methods. As shown in Figure 18, the distribution of DSC values demonstrates a moderate spread, reflecting differences in task complexity and model effectiveness.

**Table 22:** Performance metrics of transformer-based models in medical image registration studies.

References	Dataset	DSC (%)	HD (mm)	NJD (%)	Runtime (s)
Bai et al. (2024)	IXI	78.80	4.25	NR	NR
Chen et al. (2022)	IXI	74.50	NR	NR	NR
Chen Z. et al. (2024)	LPBA40	69.70	6.40	NR	0.325
Huang C. et al. (2025)	SZC-T1	80.90	4.60	NR	NR
Jia et al. (2025)	IXI	76.5	NR	0.148	0.385
Li W. et al. (2024)	OASIS	89.60	NR	NR	0.36
Liu et al. (2026)	LPBA40	68.57	17.99	NR	NR
Ma et al. (2022)	OASIS	75.70	NR	NR	NR
Meng et al. (2025)	OASIS	81.40	NR	1.07	0.96
Shi et al. (2022)	MM-WHS	86.90	NR	NR	NR
Yang X. et al. (2025)	IXI	74.80	NR	NR	0.34
Zhang X. et al. (2025)	OASIS	82.40	NR	NR	NR
Zhou and Cao (2025)	OASIS	81.40	NR	0.20	0.29
Wang H. et al. (2024)	LPBA40	73.10	NR	NR	NR

NR, Not Reported in the original study.

Analysis by dataset, as shown in Figure 19, shows that studies conducted on the OASIS dataset tend to achieve higher DSC values, with several results exceeding 80%, including Li W. et al. (2024) and Zhang X. et al. (2025). In contrast, lower DSC values are observed on LPBA40, with reported values as low as 68.57% (Liu et al., 2026), suggesting greater difficulty or variability in registration performance on this dataset. HD values, reported in a limited number of studies, range from 4.25 mm (Bai et al., 2024) to 17.99 mm (Liu et al., 2026). As illustrated in Figure 20, this wide spread highlights substantial variation in boundary alignment accuracy across methods, with some approaches achieving relatively precise alignment while others exhibit significantly larger errors. Runtime performance, where reported, generally remains below 1 second, with values ranging from 0.29 s (Zhou and Cao, 2025) to 0.96 s (Meng et al., 2025). The results described in Table 22 suggest that most transformer-based approaches achieve relatively efficient inference, although some variability exists depending on model complexity. The relationship between DSC and runtime Figure 21 does not indicate a clear trade-off, as both high and moderate DSC values are observed across a range of runtimes. This suggests that improved registration accuracy is not necessarily associated with increased computational cost. Transformer-based models demonstrate strong and consistent performance in medical image registration, particularly in terms of overlap-based accuracy. However, variability across datasets and the limited reporting of complementary metrics such as HD and NJD restricts a comprehensive comparison between studies. These findings emphasize the importance of more standardized evaluation and reporting practices in future work.

In medical image registration, the performance analysis of transformer-based models should be done with more caution than in other tasks, because registration itself includes several different methodological families. Rigid registration, affine registration, and deformable registration differ not only in the type of transformation, but also in their purpose, difficulty level, evaluation criteria, and clinical application. Therefore, the results in this area should not be interpreted as a single group.

The main advantage of transformers in registration is related to their ability to model long-range spatial relationships between a fixed image and a moving image. In registration, the model must recognize the relationship between corresponding structures in two images; these images may be from two different times, two different patients, two different modalities, or two different treatment stages. Transformer-based cross-attention, multi-scale self-attention, and feature matching can help learn this correspondence, especially when there are complex anatomical changes or the initial distance between the two images is large.

However, registration is not just a matter of increasing Dice or reducing the overall error. In deformable registration, the model should produce a reasonable, smooth, and anatomically valid deformation field in addition to improving the overlap of structures. A method may report a high Dice but its deformation field may have folding, spurious changes, or topology violations. For this reason, metrics such as Jacobian determinant, folding rate, smoothness, inverse consistency, and landmark error should be evaluated alongside Dice or HD95. The absence of these metrics leaves the assessment of the true quality of registration incomplete.

In the reviewed studies, transformer-based deformable methods have been commonly used for brain MRI, multimodal registration, radiotherapy planning, cardiac imaging, and atlas-based registration. These methods show that attention can be useful in understanding large-scale, nonlocal relationships between images. However, comparing them with rigid or affine methods is not scientifically correct, because rigid and affine methods only model global changes, while deformable methods produce dense displacement fields. Therefore, the discussion of registration performance should always be transformation-aware.

Compared to CNNs, transformers show more advantages in registration when the problem requires long-range correspondence, complex transformations, or multiscale feature fusion. But powerful CNNs, especially U-Net, VoxelMorph, and diffeomorphic network-based models, are still very competitive baselines. In many cases, CNN–Transformer hybrid models are more practical than purely attention-based models, because they both preserve local and boundary information and learn large-scale relationships between images.

Unsolved problems in registration remain serious. First, the anatomical validity of the deformation field is still not fully guaranteed. Second, multimodal registration remains difficult due to the differences in intensity, contrast, and texture properties between modalities. Third, the actual evaluation of registration requires accurate ground truth, which is not available in many medical applications. Fourth, high performance on a particular dataset or anatomy is not necessarily generalizable to other anatomy. Fifth, for clinical use, the model must demonstrate not only accuracy, but also stability, speed, uncertainty, and predictable behavior in difficult cases. Thus, registration is one area that, although it has benefited from transformers, has not yet been fully resolved in terms of standardized evaluation, clinical validity, and assurance of valid physical deformation.

### 6.5 Reproducibility and open-source availability

Reproducibility is a fundamental criterion for evaluating the practical and research value of AI-based models in medical image analysis. In the reviewed studies, the performance of the models is usually affected by factors such as the type of dataset, the way the training and test data are split, the validation strategy, the preprocessing settings, the hyperparameters, the initial weights, and the hardware conditions. Therefore, simply reporting performance metrics without providing the code, the trained weights, the evaluation scripts, or sufficient implementation details is not sufficient for independent reproducibility of the results. This is even more important in the field of medical imaging, where limited access to clinical data, differences between imaging protocols, heterogeneity between medical centers, and the possibility of data leakage can make the performance reported in one study not easily replicated in another setting.

To address this issue, Table 23 summarizes the reproducibility status and availability of implementation resources for a selected set of reviewed methods. This table shows that the status of open-source access is highly heterogeneous across studies. Some models, such as those that provide public code and trained weights, are more robust in terms of their ability to be reimplemented and used in independent benchmarking. In contrast, in a significant proportion of studies, the code, model weights, or evaluation scripts are not explicitly reported, making it more difficult to interpret results, compare fairly with standard baselines, and reuse models. Therefore, in this review, access to implementation sources is considered as a complementary indicator for assessing the operational validity of studies, rather than simply a technical feature.

**Table 23:** Reproducibility and open-source availability of representative reviewed methods.

Model	Task	Domain/modality	Evaluation scope	Code	Weights	Eval.	Level	Reproducibility note
UCTNet (Guo et al., 2024)	Segmentation	Synapse, ACDC, ISIC2018	Multi-dataset segmentation	Yes	–	–	Medium	Public code improves reproducibility, but pre-trained weights and standardized evaluation scripts were not clearly reported.
Med-SA (Wu J. et al., 2025)	Segmentation	Multimodal medical images	Multimodal segmentation	Yes	–	–	Medium	Released code supports reproducibility of SAM adaptation, but weights and evaluation scripts require repository-level verification.
MedSAM (Ma et al., 2024a)	Promptable segmentation	Multimodal medical images	Large-scale medical segmentation	Yes	Yes	–	High	Foundation model with public implementation and released pre-trained weights; performance remains prompt- and domain-dependent.
U-Mamba (Ma et al., 2024b)	Segmentation	2D and 3D biomedical images	Multi-dataset segmentation	Yes	–	Yes	Medium	Official implementation includes evaluation resources, but pre-trained weights were not clearly identified as generally available.
PFormer (Gao et al., 2025)	Segmentation	3D medical images	3D segmentation benchmarks	Yes	Yes	–	High	Public code and model availability make it suitable for reproducible 3D segmentation benchmarking.
I2U-Net (Dai et al., 2024)	Segmentation	Skin lesion, polyp, brain tumor, and multi-organ images	Multiple segmentation datasets	Yes	–	–	Medium	Public code is useful for comparison, although pre-trained weights and evaluation scripts were not clearly reported.
SegVol (Du et al., 2024)	Segmentation	3D volumetric CT	Universal volumetric segmentation	–	–	–	Limited	Large-scale evaluation is informative, but open implementation resources were not clearly reported in the reviewed record.
TBConvL-Net (Iqbal et al., 2025)	Segmentation	MRI, X-ray, fundus, ultrasound, and microscopy	Ten public datasets	–	–	–	Limited	Broad multimodal evaluation is useful, but code and weights were not explicitly reported.
H-VMUNet (Wu R. et al., 2025)	Segmentation	ISIC2017, spleen, and colonoscopy	Three segmentation datasets	–	–	–	Limited	Efficiency gains are reported, but implementation resources were not clearly identified.
U-KAN (Li C. et al., 2025)	Segmentation/ generation	Medical image segmentation and generation	Task-specific benchmarks	–	–	–	Limited	KAN-based design is relevant as a transformer alternative, but open resources were not clearly reported.
SMM-UNet (Li G. et al., 2025)	Segmentation	Medical image segmentation	Reported segmentation benchmarks	–	–	–	Limited	Model contributes to segmentation comparison, but code, weights, and evaluation scripts were not clearly reported.
UNI (Chen R. J. et al., 2024)	Foundation model	Computational pathology/WSI	Large-scale pathology benchmarks	Yes	Req.	–	High	Public code and access-controlled model weights support downstream pathology evaluation and transfer learning.
CONCH (Lu et al., 2024)	Vision–language foundation model	Computational pathology	Image–text pathology benchmarks	Yes	Req.	–	High	Public implementation supports multimodal pathology analysis; model-weight access may require controlled download or request.
TC-ViT (Sun et al., 2023)	Classification	Thyroid ultrasound	Single-task dataset	Planned	–	–	Medium	Code availability was announced, but pre-trained weights and evaluation scripts were not clearly reported.
MIL-ViT (Bi et al., 2023)	Classification	Fundus imaging	Multiple public benchmarks	Yes	Yes	–	High	Public code and pre-trained models support independent reuse and comparison.
SI-ViT (Zhang et al., 2024)	Classification	Pancreatic cytopathology	ROSE and pathology datasets	–	–	–	Limited	Reproducibility depends mainly on the reported experimental protocol and dataset accessibility.
DWNAT-Net (Yan et al., 2025)	Classification	Breast histopathology	BreakHis and BACH	–	–	–	Limited	Frequency-domain transformer design is reported, but implementation resources were not explicitly identified.
MedViT (Nejati Manzari et al., 2023)	Classification	MedMNIST medical images	Large standardized benchmark suite	–	–	–	Limited	Benchmark diversity improves interpretability, but public implementation resources were not explicitly reported.
BOLT (Li Y. et al., 2024)	Classification	Skin lesion, fracture grading, diabetic retinopathy	Three medical classification tasks	–	–	–	Limited	Self-supervised protocol is described, but code, weights, and evaluation scripts were not clearly reported.
DBCvT (Li J. et al., 2024)	Classification	Medical image classification	Multiple medical datasets	–	–	–	Limited	Hybrid CNN–Transformer design is reported, but open implementation resources were not clearly identified.
Eff-CTM (Liu S. et al., 2024)	Classification	Chest X-ray, histopathology, and dermatology	Three public datasets	–	–	–	Limited	Efficiency is discussed, but implementation resources were not explicitly reported.
Multi-Scale ViT (Alqahtani et al., 2025)	Classification/retrieval	ISIC-2018 and ChestX-ray14	Two public benchmarks	–	–	–	Limited	Useful for diagnostic retrieval comparison, but reproducibility resources were not clearly reported.
MBWO-CrossViT (Dahou et al., 2025)	Classification	Multi-dataset medical classification	Benchmark datasets	–	–	–	Limited	Metaheuristic feature selection adds implementation complexity; code and weights were not clearly reported.
Robust Swin Transformer (Kumar et al., 2025)	Classification	High-dimensional medical images	Reported benchmark setting	–	–	–	Limited	Computational performance is discussed, but implementation resources were not explicitly reported.
MedViTV2 (Nejati Manzari et al., 2026)	Classification	Medical image corruption benchmarks	17 datasets and 12 corrupted datasets	–	–	–	Limited	Broad robustness evaluation is valuable, but public code and pre-trained weights were not clearly reported.
Paralleformer (Wang et al., 2026)	Classification	Histopathology	Public and self-built datasets	–	–	–	Limited	Use of mixed datasets improves evaluation breadth, but reproducibility resources were not explicitly reported.
ViT–Perceiver IO (Khaliq et al., 2025)	Classification	MRI, X-ray, dermoscopy	Multi-disease datasets	–	–	–	Limited	Clinical interface is described, but code, weights, and evaluation scripts were not clearly reported.
ViT-ARDNet-LSTM (Parvathy et al., 2025)	Classification	Brain MRI	ASD classification dataset	–	–	–	Limited	Optimization-based pipeline is reported, but reproducibility resources were not clearly identified.
MMR-Mamba (Zou et al., 2025)	Reconstruction	Multimodal MRI	BraTS and fastMRI knee	Yes	–	–	Medium	Public code supports MRI reconstruction reproducibility, but pre-trained weights and evaluation scripts were not clearly reported.
CS-SwinGAN (Zhang et al., 2025a)	Reconstruction	Multi-coil MRI	MRI reconstruction and noise settings	Yes	–	–	Medium	Source code is reported as publicly available, improving implementation-level reproducibility.
R3ST (Liu J. et al., 2024)	Spectral reconstruction	Fundus imaging	Spectral reconstruction benchmarks	Yes	–	–	Medium	Public code supports reproduction of spectral reconstruction experiments.
MHWT (Han and Du, 2025)	Reconstruction	Multimodal MRI	MRI reconstruction datasets	Yes	–	–	Medium	Reported code availability supports reproducibility, but weights and evaluation scripts were not clearly reported.
DiTMSR (Tu et al., 2025)	Super-resolution	MRI	Public and clinical datasets	–	–	–	Limited	Clinical and public evaluation is useful, but implementation resources were not clearly reported.
TIME-Net (Zhang et al., 2023)	Reconstruction	Dual-energy CBCT	Simulated and real rat data	–	–	–	Limited	Use of simulated and real data is informative, but open implementation resources were not explicitly reported.
Multi-view Transformer GAN (Lyu et al., 2023)	Reconstruction	Cardiac cine MRI	Cardiac cine reconstruction	–	–	–	Limited	Reconstruction protocol is described, but code, weights, and evaluation scripts were not clearly reported.
Transformer-GAN (Wang Y. et al., 2024)	Reconstruction	PET and MRI	Phantom and clinical datasets	–	–	–	Limited	Clinical evaluation improves relevance, but public implementation resources were not clearly identified.
DDFCA-Net (Chen et al., 2025)	Reconstruction	MRI	CC359-Brain and fastMRI knee	–	–	–	Limited	Dual-domain evaluation is reported, but open implementation resources were not clearly identified.
DdeNet (Lin et al., 2024)	Reconstruction	Sparse-view CT	Sparse-view CT reconstruction	–	–	–	Limited	Dual-domain reconstruction is described, but code and weights were not clearly reported.
WUTrans-MoCo (Yuan et al., 2025)	Reconstruction/registration	4D-CBCT	Two public lung CT/CBCT datasets	–	–	–	Limited	Use of public datasets improves comparability, but implementation resources were not clearly reported.
VRF-Net (M.khair et al., 2026)	Reconstruction	Magnetic particle imaging	Open MPI and simulated data	–	–	–	Limited	Dataset description supports interpretation, but code and pre-trained weights were not explicitly reported.
SVFR (Yang T. et al., 2025)	Reconstruction	3D MRI	Large 3D brain and knee datasets	–	–	–	Limited	Computational efficiency is reported, but open resources were not clearly identified.
SymTrans (Ma et al., 2022)	Registration	Medical image registration	Unsupervised registration benchmarks	Yes	–	–	Medium	Public code improves reproducibility of the registration pipeline.
uniGradICON (Tian et al., 2024)	Registration	Multi-dataset medical registration	Large multi-dataset evaluation	Yes	Yes	–	High	Public code and model weights provide strong support for independent benchmarking.
TransMatch (Chen Z. et al., 2024)	Registration	Deformable registration	Unsupervised registration benchmarks	–	–	–	Limited	Method is relevant for feature matching, but implementation resources were not clearly reported in the reviewed record.
SparseMorph (Bai et al., 2024)	Registration	Mono- and multi-modal registration	Deformable registration datasets	–	–	–	Limited	Sparse deformation modeling is reported, but public code and weights were not clearly identified.
SCT-Net (Liu X. et al., 2025)	Registration	Cervical cancer radiotherapy	Radiotherapy registration setting	–	–	–	Limited	Clinical task relevance is high, but implementation resources were not explicitly reported.
Wavelet-linear attention registration (Li W. et al., 2024)	Registration	Deformable medical image registration	Registration benchmarks	–	–	–	Limited	Efficient attention design is relevant, but code, weights, and evaluation scripts were not clearly reported.
AutoFuse (Meng et al., 2025)	Registration	Inter-patient brain MRI	Brain MRI registration	–	–	–	Limited	Registration method is task-specific; open resources were not clearly identified.
Decoder-only registration (Jia et al., 2025)	Registration	Medical image registration	Reported registration datasets	–	–	–	Limited	Decoder-only design is relevant for comparison, but reproducibility resources were not clearly reported.

“Yes” indicates that the corresponding resource is explicitly reported as publicly available in the original study or identified from an official project repository. “Planned” indicates that future release is stated but availability should be verified before use. “Req.” indicates that access to model weights may require registration, request, or controlled download. “–” indicates that the corresponding item is not explicitly reported or could not be identified from the reviewed record. Reproducibility level is assigned as follows: *High* = public code with pre-trained weights/models or strong reusable implementation resources; *Medium* = public code or partial implementation resources without clearly reported pre-trained weights; *Limited* = no public implementation resource explicitly reported.

According to this review, studies that provide publicly available code or trained weights provide stronger evidence for independent review, testing on external data, error analysis, and further development. However, even access to code and model weights alone is not sufficient for clinical readiness. To reliably transfer these models to real-world settings, open-source resources need to be accompanied by transparent data reporting, patient-level splitting, external validation, multicenter testing, uncertainty analysis, calibration checks, and assessment of the impact of the model on clinical workflow. Hence, numerical results from studies lacking public implementation resources should be interpreted with more caution, even if their reported performance on the original dataset is high.

In this review, the reproducibility status of studies is assessed from several perspectives: public access to implementation code, publication of trained weights, provision of evaluation or inference scripts, adequate explanation of the dataset and data partitioning, reporting of hyperparameters, existence of comparable baselines, and transparency in preprocessing and post-processing procedures. Studies that, in addition to reporting results, made the model code or weights available are of greater practical value in terms of reviewability, reimplementation, and research use. In contrast, studies that only reported the final results but did not provide implementation sources or sufficient details for reproducibility should be interpreted with more caution.

A review of the literature shows that the status of open-source availability in this area is heterogeneous. Some models have published public code, trained weights, or usable implementations and can therefore be considered as more suitable options for re-comparison, benchmarking, and further development. In contrast, a significant proportion of studies, especially some newer models or applications based on proprietary data, lack public code or weights. This makes it more difficult to replicate results, independently assess, analyze errors, and compare them fairly with standard baselines.

From a clinical application perspective, reproducibility is not simply a technical characteristic but is part of the validity of the model's translatability. A model for which code, weights, evaluation protocol, and implementation details are available allows for independent testing on external data, robustness checks, failure mode analysis, calibration, and comparisons with standard methods. Therefore, in interpreting the results of this review, studies with public sources and transparent protocols provide stronger evidence for research use and further development than studies without such information. However, access to code or model weights alone is not sufficient for clinical readiness and should be considered in conjunction with external validation, multicenter testing, review of heterogeneous data, and assessment of the model's impact on clinical workflow.

### 6.6 Computational cost and efficiency considerations

Computational efficiency is a critical factor in evaluating transformer-based methods for medical image analysis, particularly because many medical imaging tasks involve high-resolution 2D images, volumetric 3D scans, multimodal inputs, or dense pixel-/voxel-level predictions. Although transformer-based architectures can improve global context modeling, they may also introduce substantial computational overhead due to self-attention, large parameter counts, high memory consumption, and expensive inference on volumetric data. Therefore, performance metrics such as accuracy, Dice score, PSNR, SSIM, or registration error should be interpreted together with computational indicators such as the number of parameters, FLOPs or GFLOPs, GPU memory usage, inference time, input resolution, batch size, and hardware configuration.

The reviewed studies show heterogeneous reporting of computational cost. Some works explicitly report parameters, FLOPs, inference speed, or memory usage, especially lightweight or efficiency-oriented models such as PFormer, lightweight segmentation networks, SVFR, and several registration methods. However, many studies report only task-specific performance metrics and omit key efficiency indicators, making systematic cross-method comparison difficult. This limitation is particularly important for clinical translation, where model deployment may be constrained by GPU memory, inference latency, image size, integration with PACS or radiotherapy planning systems, and the need for real-time or near-real-time processing.

To improve transparency, Table 24 summarizes the computational-cost reporting status of representative reviewed methods across segmentation, classification, reconstruction, and registration. Values are reported only when explicitly provided in the original studies or source records; otherwise, missing items are indicated by “–.” Importantly, runtime values should not be directly compared unless they are measured under comparable conditions, including the same hardware, input size, batch size, preprocessing pipeline, dimensionality, and inference protocol. For example, inference time reported for a 2D image classification model is not directly comparable with runtime for 3D deformable registration or volumetric MRI reconstruction. Furthermore, in order to address the heterogeneity in computational-cost reporting, Table 24 summarizes the availability of parameter counts, FLOPs or computational-complexity indicators, memory usage, standardized inference time, hardware/input context, and reporting completeness across representative reviewed methods.

**Table 24:** Computational cost and efficiency reporting across representative reviewed methods.

Model	Task	Domain/setting	Parameters	FLOPs/ computational cost	Memory usage	Runtime/ inference	Hardware/input context	Reporting level
LMIS-xxs (Zhu et al., 2024)	Segmentation	Skin lesion, intestinal lesion, and cell nuclei segmentation	0.524M	0.197 GFLOPs	–	0.014 s/image	RTX 3090; ISIC2017/ISIC2018; 71.26 FPS reported	Strong
UCTNet (Guo et al., 2024)	Segmentation	Synapse, ACDC, ISIC2018	–	–	–	–	Multi-dataset segmentation	Limited
PFormer (Gao et al., 2025)	Segmentation	3D medical image segmentation	Reported lower; exact value–	Reported lower; exact value–	–	–	Synapse and ACDC datasets	Partial quantitative
LightM-UNet (Liao et al., 2024)	Segmentation	2D and 3D organ/lesion segmentation	1–1.87M	267–457 GFLOPs	–	–	LiTS and lung X-ray datasets	Strong
SMM-UNet (Li G. et al., 2025)	Segmentation	ISIC2017, ISIC2018, BUSI	0.038M	Linear-complexity design; exact GFLOPs–	–	–	Lesion segmentation datasets	Partial quantitative
TBConvL-Net (Iqbal et al., 2025)	Segmentation	MRI, X-ray, fundus, ultrasound, and microscopy	9.6M	15.5 GFLOPs	–	0.0191 s/image	Ten public datasets; original runtime 19.1 ms	Strong
H-VMUNet (Wu R. et al., 2025)	Segmentation	ISIC2017, spleen, CVC-ClinicDB	67% lower than VM-UNet	82% lower computational complexity than VM-UNet	–	–	Three segmentation datasets	Relative quantitative
Lightweight 3D MMI model (Hua et al., 2024)	Segmentation	ACDC, BraTS2018, temporal bone CT	9.63M	–	–	–	3D multi-target semantic segmentation	Partial quantitative
BGHNet (Liu H. et al., 2024)	Segmentation	Lung tumor CT segmentation	–	–	–	–	HUST-Lung and MSD-Lung datasets	Limited
HiDiff (Chen T. et al., 2024)	Segmentation	Organs, tumors, vessels, polyps	–	Iterative refinement increases inference cost; exact value–	–	–	Synapse, BraTS2021, Kvasir-SEG, CVC-ClinicDB, DRIVE, and CHASE DB1	Partial qualitative
Swin-UMamba† (Liu J. et al., 2025)	Segmentation	AbdomenMRI, EndoVis, and microscopy	28M	18.9 GFLOPs	–	100 training epochs reported	AMOS 2022, EndoVis 2017, and NeurIPS 2022 Cell Segmentation	Strong
SegVol (Du et al., 2024)	Segmentation	Universal 3D volumetric CT segmentation	–	Efficiency evaluated; exact value–	–	–	25 CT datasets; 90k unlabeled CT; 22 tasks	Partial qualitative
U-KAN (Li C. et al., 2025)	Segmentation/ generation	Medical image segmentation and generation	–	Lower computation cost reported; exact value–	–	–	Medical segmentation benchmarks	Partial qualitative
Trans-VNet (Wang C. et al., 2024)	Segmentation	Tooth semantic segmentation in CBCT	–	–	–	–	CBCT tooth segmentation	Limited
Med-SA (Wu J. et al., 2025)	Segmentation	Multimodal medical image segmentation	–	–	–	–	SAM adaptation for medical segmentation	Limited
MedSAM (Ma et al., 2024a)	Promptable segmentation	Multimodal medical image segmentation	–	–	–	–	Medical foundation segmentation model	Limited
U-Mamba (Ma et al., 2024b)	Segmentation	2D and 3D biomedical segmentation	–	Linear-complexity SSM design; exact value–	–	–	Biomedical segmentation datasets	Partial qualitative
UNI (Chen R. J. et al., 2024)	Foundation model	Computational pathology/WSI	–	–	–	–	Large-scale pathology representation learning	Limited
CONCH (Lu et al., 2024)	Vision–language foundation model	Computational pathology	–	–	–	–	Image–text pathology benchmarks	Limited
TC-ViT (Sun et al., 2023)	Classification	Thyroid ultrasound	–	–	–	–	ROI-based ultrasound images	Limited
MIL-ViT (Bi et al., 2023)	Classification	Fundus image classification	–	–	–	–	Fundus image benchmarks	Limited
SI-ViT (Zhang et al., 2024)	Classification	Pancreatic cytopathology/ROSE images	–	–	–	–	Pathology image classification	Limited
DWNAT-Net (Yan et al., 2025)	Classification	Breast histopathology images	–	Efficient neighborhood attention discussed; exact value–	–	–	BreakHis and BACH datasets	Partial qualitative
MedViT (Nejati Manzari et al., 2023)	Classification	MedMNIST-based medical image classification	–	–	–	–	Standardized MedMNIST benchmarks	Limited
BOLT (Li Y. et al., 2024)	Classification	Skin lesion, fracture grading, and diabetic retinopathy	–	–	–	–	Three medical classification tasks	Limited
DBCvT (Li J. et al., 2024)	Classification	Medical image classification	–	–	–	–	Multiple medical datasets	Limited
Eff-CTM (Liu S. et al., 2024)	Classification	Chest X-ray, histopathology, and dermatology	–	Efficiency-oriented design; exact value–	–	–	Three public datasets	Partial qualitative
Multi-Scale ViT (Alqahtani et al., 2025)	Classification/retrieval	ISIC-2018 and ChestX-ray14	–	–	–	–	Two public benchmarks	Limited
MBWO-CrossViT (Dahou et al., 2025)	Classification	Multi-dataset medical image classification	–	–	–	–	Benchmark medical datasets	Limited
Robust Swin Transformer (Kumar et al., 2025)	Classification	High-dimensional medical images	–	Computational performance discussed; exact value–	–	–	Reported benchmark setting	Partial qualitative
MedViTV2 (Nejati Manzari et al., 2026)	Classification	Medical image corruption and robustness benchmarks	–	–	–	–	17 datasets and 12 corrupted datasets	Limited
Paralleformer (Wang et al., 2026)	Classification	Histopathology	–	Focus linear attention for efficiency; exact value–	–	–	Public and self-built pathology datasets	Partial qualitative
ViT–Perceiver IO (Khaliq et al., 2025)	Classification	MRI, X-ray, dermoscopy, and multi-disease diagnosis	–	Computational efficiency discussed; exact value–	–	–	Multi-disease imaging datasets	Partial qualitative
YOLOv4+ ASFF Swin Transformer (Pan et al., 2024)	Detection/classification	Renal incidentaloma CT images	–	–	–	Competitive inference speed; exact value–	CT image detection setting	Partial qualitative
MMR-Mamba (Zou et al., 2025)	Reconstruction	Multimodal MRI reconstruction	–	Linear-complexity Mamba design; exact value–	–	–	BraTS and fastMRI knee datasets	Partial qualitative
CS-SwinGAN (Zhang et al., 2025a)	Reconstruction	Multi-coil MRI reconstruction	High parameter burden reported; exact value–	High computational demand reported; exact value–	–	–	MRI compressed-sensing reconstruction	Partial qualitative
SVFR (Yang T. et al., 2025)	Reconstruction	3D MRI reconstruction	–	Slice-to-volume strategy reduces 3D computational burden	About 40% lower than 3D U-Net	–	3D brain and Stanford 3D FSE knee datasets	Partial quantitative
DiTMSR (Tu et al., 2025)	Super-resolution	MRI super-resolution	–	Efficient hybrid Mamba decoder discussed; exact value–	–	–	Public and clinical MRI datasets	Partial qualitative
TIME-Net (Zhang et al., 2023)	Reconstruction	Dual-energy CBCT artifact removal	–	–	–	–	Simulated and real rat DE-CBCT data	Limited
Multi-view Transformer GAN (Lyu et al., 2023)	Reconstruction	Cardiac cine MRI reconstruction	–	–	–	–	Cardiac cine MRI reconstruction	Limited
Transformer-GAN (Wang Y. et al., 2024)	Reconstruction	PET and MRI reconstruction	–	–	–	–	Phantom and clinical datasets	Limited
DDFCA-Net (Chen et al., 2025)	Reconstruction	MRI reconstruction	–	–	–	–	CC359-Brain and fastMRI knee	Limited
DdeNet (Lin et al., 2024)	Reconstruction	Sparse-view CT reconstruction	–	–	–	–	Sparse-view CT reconstruction	Limited
WUTrans-MoCo (Yuan et al., 2025)	Reconstruction/ motion compensation	4D-CBCT reconstruction	–	–	–	–	Public lung CT/CBCT datasets	Limited
R3ST (Liu J. et al., 2024)	Spectral reconstruction	Fundus spectral reconstruction	–	–	–	–	Fundus image spectral reconstruction	Limited
MHWT (Han and Du, 2025)	Reconstruction	Multimodal MRI reconstruction	–	–	–	–	MRI reconstruction datasets	Limited
TLBO–Affine–SURF–Projective (Arora et al., 2024)	Registration	Rigid/affine multimodal registration	Not applicable	Optimization-based; no DL FLOPs reported	–	–	Whole Brain Atlas and Kaggle datasets	Limited
SparseMorph (Bai et al., 2024)	Registration	single-modality and multimodal deformable registration	–	Sparse attention design; exact value–	–	–	IXI, OASIS, MMWHS	Partial qualitative
TransMorph (Chen et al., 2022)	Registration	3D deformable MRI/CT registration	–	Heavy architecture/high GPU demand reported; exact value–	High GPU demand; exact value–	–	IXI, OASIS, brain atlas, XCAT-CT	Partial qualitative
TransMatch (Chen Z. et al., 2024)	Registration	Unsupervised deformable registration	–	–	Memory reported in study; exact value–	0.325 s/volume	LPBA40, IXI, OASIS	Partial quantitative
LessNet (Jia et al., 2025)	Registration	Multi-organ unsupervised deformable registration	Minimal parameters reported; exact value–	Lightweight decoder-only design; exact value–	–	0.385 s/volume	13 public datasets across brain, cardiac, abdomen	Partial quantitative
LWSA registration (Li W. et al., 2024)	Registration	Deformable brain MRI registration	–	Reduced attention cost reported; exact value–	–	0.360 s/volume	LPBA40, IXI, OASIS	Partial quantitative
SymTrans (Ma et al., 2022)	Registration	Unsupervised medical image registration	–	CEMSA reduces attention overhead; exact value–	–	–	Unsupervised registration setting	Partial qualitative
AutoFuse/AutoFuse-Trans (Meng et al., 2025)	Registration	Inter-patient brain MRI and cardiac MRI registration	–	–	–	0.960 s/volume	Eight public datasets	Partial quantitative
TCDE-Net (Yang X. et al., 2025)	Registration	3D brain MRI deformable registration	–	–	–	0.340 s/volume	OASIS, IXI, Hammers-n30r95	Partial quantitative
WaveMorph (Zhang X. et al., 2025)	Registration	Unsupervised brain MRI registration	–	Lightweight wavelet-guided ConvNeXt design; exact value–	–	0.072 s/image	IXI and OASIS brain MRI	Partial quantitative
H-SGANet (Zhou and Cao, 2025)	Registration	Volumetric brain MRI registration	–	–	–	0.290 s/volume	OASIS and LPBA40	Partial quantitative
MCANet (Liu et al., 2026)	Registration	3D brain MRI deformable registration	–	–	–	–	OASIS, LPBA40, IXI	Limited
SCT-Net (Liu X. et al., 2025)	Registration	Cervical cancer radiotherapy image registration	–	–	–	–	EBRT–ICBT deformable registration	Limited
RDP-Net (Wang H. et al., 2024)	Registration	Large-deformation brain MRI registration	–	Lightweight CNN pyramid design; exact value–	–	–	LPBA40, Mindboggle, IXI	Partial qualitative
NIR (Sun et al., 2024)	Registration	Neural-field deformable registration	–	Optimization-based neural field; exact value–	–	–	Mindboggle101 and OASIS	Partial qualitative
uniGradICON (Tian et al., 2024)	Registration	General medical image registration	–	Very large training cost reported; exact value–	–	–	12 public datasets; zero-shot and fine-tuning settings	Partial qualitative
M2M-Reg (Choo et al., 2026)	Registration	Extremely heterogeneous multimodal registration	–	Complex pipeline reported; exact value–	–	–	ADNI PET–MRI and FA–MRI	Partial qualitative

Runtime values were standardized to seconds whenever the original unit allowed conversion. For example, 19.1 ms was reported as 0.0191 s, and 71.26 FPS was converted to approximately 0.014 s/image. “–” indicates that the corresponding computational-cost item was not explicitly reported or could not be extracted from the reviewed record. Relative entries, such as percentage reductions in parameters, FLOPs, memory, or computational complexity, were retained only when exact absolute values were not available. Reporting levels were assigned as follows: *Strong* = at least two quantitative efficiency indicators were reported; *Relative quantitative* = efficiency was reported as a relative reduction against a baseline; *Partial quantitative* = one quantitative efficiency indicator was reported; *Partial qualitative* = computational efficiency was discussed without exact values; *Limited* = no explicit computational-cost information was identified. Direct runtime comparisons should be avoided unless hardware, input size, batch size, dimensionality, preprocessing, and inference protocol are comparable across methods.

Accordingly, computational cost in this review is interpreted descriptively rather than as a formal efficiency ranking. Models that combine strong performance with low parameters, reduced FLOPs, or lower memory usage may be more suitable for resource-constrained clinical environments, but such conclusions require standardized reporting and external benchmarking. Future studies should routinely report parameter count, FLOPs/GFLOPs, peak GPU memory, inference time per image or per volume, hardware configuration, input resolution, batch size, and code availability to enable fair and reproducible efficiency comparison.

### 6.7 Limitations and future directions

Despite the rapid growth of transformer-based models in medical image analysis, the present review shows that the available evidence still has important methodological, computational, and clinical limitations. These limitations should be considered in the interpretation of study results as well as in the design of future research. First, a major limitation of the existing literature is the considerable heterogeneity in datasets, tasks, evaluation criteria, data splits, validation methods, and comparative baselines. Many studies have reported the performance of models on different datasets and protocols; therefore, direct comparisons of accuracy, Dice score, PSNR, SSIM, or registration error across studies can be misleading. This issue is particularly serious when a single dataset is used in different studies for different tasks, such as binary classification, multiclass classification, 2D segmentation, or 3D segmentation. As a result, high numerical performance should always be interpreted in the context of the task, modality, annotation type, class structure, evaluation criteria, and validation protocol of the same study.

Second, the available evidence is not yet sufficient to draw firm conclusions about the general superiority of transformers over convolutional networks. Although many studies have reported that transformer-based or hybrid models can outperform convolutional baselines on some tasks, these comparisons have not always been conducted under completely fair conditions. Differences in model size, pre-training, number of training epochs, augmentation, hyperparameter settings, data split, and the strength of the selected baselines can affect the results. Therefore, the future direction should move toward controlled comparisons; that is, transformer, convolutional, hybrid models, and alternative architectures should be evaluated on the same data, similar split, common criteria, comparable training settings, and robust baselines. Without such conditions, any general claim about the superiority of one architectural family over another should be made with caution.

Third, the present review shows that many studies still lack sufficient reproducibility. In a significant proportion of the reviewed methods, the code, trained weights, evaluation scripts, detailed preprocessing details, training settings, and inference protocol are not fully reported. This makes it difficult to reimplement the results, independently test them on external data, analyze errors, and perform fair benchmarking. As shown in Table 23, only a fraction of the studies reported public implementation sources or usable weights. Therefore, one important future direction is to require transparent reporting of the code, model weights, training settings, evaluation scripts, random seeds, data splitting method, and inference details. Such transparency is essential not only for academic research, but also for assessing the transferability of models to clinical settings.

Fourth, the computational cost of models remains a serious limitation. Many transformer-based models, especially in 3D tasks, volume reconstruction, multi-organ segmentation, and deformable registration, require high GPU memory, a large number of parameters, and significant inference time. However, as summarized in Table 24, not all studies have reported the number of parameters, FLOPs, memory consumption, inference time, input size, batch size, and hardware used. This deficiency makes it difficult to assess the deployability of models in real-world environments. Future research should report computational efficiency indicators in a standardized manner in addition to performance metrics. Also, the development of lightweight architectures, efficient attention, low-cost multiscale models, state-space-based methods, pruning, distillation, and memory-efficient strategies for processing large 3D images will be increasingly important.

Fifth, generalizability, external validation, and clinical readiness remain major unresolved challenges. Many studies have evaluated models on limited, single-center datasets or public benchmarks, while relatively few have reported performance on external datasets, multicenter cohorts, diverse patient populations, or different imaging devices and acquisition protocols. In medical imaging, differences in scanners, imaging protocols, healthcare institutions, patient demographics, disease severity, and annotation quality can substantially affect model performance in real-world settings. Although transformer-based models have demonstrated impressive results across benchmark datasets, technical performance should not be interpreted as evidence of clinical readiness. Clinical deployment requires robust validation across heterogeneous populations and healthcare environments, together with prospective clinical evaluation, workflow integration, reader studies, model calibration, uncertainty estimation, deployment assessment, and appropriate patient-level rather than image-level data splitting. Therefore, future research should place greater emphasis on comprehensive clinical validation alongside technical evaluation to ensure that transformer-based models are reliable, generalizable, interpretable, and safe for routine clinical decision support.

Sixth, interpretability, calibration, and uncertainty are not yet adequately covered in the reviewed studies. In many studies, models are mainly evaluated based on numerical metrics such as accuracy, F1-score, Dice score, PSNR, SSIM, or TRE, but it is less clear under what conditions the model makes errors, how confident it is in its prediction, and whether its decision is clinically explainable. This is particularly important in disease classification, small lesion segmentation, diagnostic image reconstruction, and deformable registration. In the future, model evaluation should include reliable attention maps, error analysis, uncertainty estimation, calibration curves, decision-confidence analysis, and reader-based evaluation to determine whether the models are not only numerically accurate but also reliable, interpretable, and clinically applicable.

Seventh, each medical imaging task has its own limitations that have not yet been fully resolved. In classification, important issues such as class imbalance, small data, imaging center bias, domain variation, interpretability, and performance in rare diseases still remain. In segmentation, challenges such as fuzzy boundaries, small structures, low-contrast lesions, inter-labeler disagreement, 3D segmentation, and preserving spatial continuity still hinder stable performance. In reconstruction, improving image similarity measures does not necessarily mean preserving full diagnostic information, and the risk of removing fine details or generating unrealistic structures must be independently assessed. In image registration, simultaneously achieving high overlap, smooth deformation field, topology preservation, invertibility, and anatomical validity still remains an open issue. These task-oriented limitations suggest that future progress should not only focus on increasing numerical performance, but also evaluate the error quality, stability, interpretability, and clinical impact of the models.

Eighth, foundation models and promptable models such as MedSAM, along with pathology foundation models and alternative architectures such as Mamba and state-space models, offer promising avenues for the future, but their application in medical imaging still requires careful evaluation. These models can improve generalizability, adaptability to different tasks, and reduce the need for extensive annotation; however, their performance may depend on prompt type, data quality, modality differences, training domain, and adaptation settings. Therefore, future research should evaluate these models not only based on average performance, but also on robustness to outliers, prompt sensitivity, computational cost, explainability, clinical safety, and behavior in difficult cases.

The future development of transformer-based models in medical image analysis should move from introducing increasingly complex architectures toward fairer evaluation protocols, stronger reproducibility, comprehensive computational-cost reporting, external validation, failure analysis, interpretability, calibration, and clinical-impact assessment. The real success of these models in the medical environment will not be achieved by improving performance metrics alone, but will require evidence that the models are reliable, efficient, and safe across heterogeneous data, different medical centers, different imaging conditions, and realistic clinical scenarios.

## 7 Conclusion

This review provides a comprehensive and structured picture of the role of transformer-based models and related architectures in medical image analysis, examining their applications in four main areas: segmentation, classification, reconstruction, and image registration. The reviewed evidence suggests that transformers have significant potential to enhance AI methods in medical imaging due to their ability to model long-range dependencies, integrate contextual information, learn multiscale representations, and support multimodal data. However, the findings of this paper indicate that the most successful approaches are often not purely transformer-based models, but rather hybrid and task-oriented architectures that combine the advantages of convolutional networks in extracting local features with the ability of transformers in modeling global context. Therefore, the role of transformers should not be interpreted as an absolute replacement for CNNs, but as a complementary and powerful framework for designing more accurate, context-aware, and flexible models in medical imaging.

A critical analysis of the studies showed that the reported performance of the models strongly depends on the type of task, imaging modality, data quality and size, validation protocol, evaluation criteria, annotation level, and strength of the comparative baseline. In classification, transformers have proven particularly useful in problems that require understanding of sparse and contextual patterns; in segmentation, multiscale and encoder-decoder architectures are important for simultaneously preserving boundary details and anatomical context; in reconstruction, combining global modeling with physical constraints and diagnostic fidelity is essential; and in image registration, a precise distinction between rigid, affine, and deformable methods is essential for valid interpretation of the results. However, the wide heterogeneity among studies, the lack of uniform reporting of code, trained weights, computational cost, data split, uncertainty, calibration, and external validation, prevent definitive conclusions about the overall superiority of one architectural family over another.

Accordingly, the main message of this review is that future progress in the application of transformers to medical imaging should not focus solely on increasing architectural complexity or reporting higher performance values, but should emphasize fair evaluation, reproducibility, computational transparency, generalizability, interpretability, multicenter validation, and real relevance to clinical needs. foundation models, promptable methods, efficient attention- or state-space-based architectures, and multimodal frameworks can provide promising avenues for the future; but their practical value can only be proven when evaluated in real-world scenarios, heterogeneous data, multicenter environments, and against meaningful clinical criteria. Transformers have opened an important horizon for the development of a new generation of intelligent medical imaging systems, but their reliable translation into clinical use requires a transition from descriptive and dataset-driven comparisons toward standardized, transparent, reproducible, and clinically-oriented assessments.
